# Prevalence of attention deficit hyperactivity disorder/hyperkinetic disorder of pediatric and adult populations in clinical settings: a systematic review, meta-analysis and meta-regression

**DOI:** 10.1038/s41380-025-03178-8

**Published:** 2025-08-28

**Authors:** Simon Johnson, Eric Lim, Peter Jacoby, Stephen V. Faraone, Benjamin Minche Su, Marco Solmi, Benjamin Forrest, Bethany Furfaro, Kiri von Klier, Jenny Downs, Wai Chen

**Affiliations:** 1 North Metropolitan Health Service, Perth, WA Australia; 2 School of Nursing, Murdoch University, Murdoch,, Perth, WA Australia; 3 Mental Health Service 5, Fiona Stanley Hospital, Murdoch, Perth, WA Australia; 4 Telethon Kids Institute, Perth, WA Australia; 5 Department of Psychiatry, Norton College of Medicine at SUNY Upstate Medical University, Syracuse, NY USA; 6 East Metropolitan Health Service, Perth, WA Australia; 7 SCIENCES lab, Department of Psychiatry, University of Ottawa, Ottawa, ON Canada; 8 Regional Centre for the Treatment of Eating Disorders and On Track: The Champlain First Episode Psychosis Program, Department of Mental Health, The Ottawa Hospital, Ottawa, ON Canada; 9 Ottawa Hospital Research Institute (OHRI) Clinical Epidemiology Program University of Ottawa, Ottawa, ON Canada; 10 Department of Child and Adolescent Psychiatry, Charité Universitätsmedizin, Berlin, Germany; 11 Armadale Mental Health Services, Perth, WA Australia; 12 Royal Perth Hospital, Perth, WA Australia; 13 Youth Mental Health, Fiona Stanley Hospital, Murdoch, Perth, WA Australia; 14 School of Allied Health, Curtin University, Perth, WA Australia; 15 Curtin Medical School, Curtin University, Bentley, Perth, WA Australia; 16 The enAble Institute, Curtin University, Bentley, Perth, WA Australia; 17 Postgraduate School of Education, University of Western Australia, Crawley, Perth, WA Australia; 18 Centre for Healthy Ageing, Murdoch University, Murdoch, Perth, WA Australia; 19 Centre of Excellence in Medical Biotechnology, Faculty of Medical Science, Naresuan University, Phitsanulok, Thailand

**Keywords:** ADHD, Psychology

## Abstract

**Background:**

Attention-Deficit/Hyperactivity Disorder (ADHD)/Hyperkinetic Disorder (HD) is linked to increased risks of morbidity, comorbidity and mortality, with higher prevalence in clinical populations. The differential prevalence of ADHD/HD across adult and pediatric clinical populations, influenced by factors such as time trends, sex, age, geographic regions, and comorbidities, has not been systematically assessed.

**Methods:**

MEDLINE, CINAHL, Embase and PsycINFO databases were searched from inception to 1st August 2023 for eligible full-text papers published in English, and reviewing reference lists of identified studies and review papers. Studies reporting ADHD/HD prevalence in adult and pediatric clinical populations were included. Meta-regression evaluated the effects of geographic region, year of publication and sample size.

**Results:**

From 30,740 citations, we reviewed 521 full-text articles, yielding 311 studies for inclusion (including 653,558 pediatric and 43,311 adult participants). Overall, worldwide pooled prevalence of ADHD/HD in clinical settings for pediatrics was 32.4% (95% CI 31–34%), and in adults 21.4% (95% CI 20–23%). Prevalence was higher in outpatient settings than inpatient settings. Prevalence based on rating scales was higher than studies using diagnostic interviews or clinical record review. Prevalence varied significantly across subspecialist settings for children and adults. No significant time trend was detected between 1981–2023. Pediatric prevalence appears influenced by geographic region but not year of publication or sample size. For adults, larger sample sizes were associated with lower prevalence estimates.

**Conclusions:**

ADHD/HD prevalence in clinical populations is 8-9-fold higher than community estimates. With these patients at risk for many adverse outcomes, our findings underscore the critical importance of resource allocation for screening, diagnosing and treatment.

## Introduction

Attention-deficit/hyperactivity disorder (ADHD) is a neurodevelopmental disorder characterized by age-inappropriate levels of inattention, hyperactivity and impulsivity, associated with persistent and pervasive impairments across the lifespan (DSM-5-TR) [1]. The World Health Organization (WHO) [2] conceptualizes a similar condition as hyperkinetic disorder (HD) in ICD-9 and ICD-10; however, in 2022, ICD-11 revised and renamed HD as “attention deficit hyperactivity disorder” (without the slash between “attention deficit” and “hyperactivity”) [2]. While there are nuanced differences between ICD-11 and DSM-5-TR versions of “ADHD” as reviewed elsewhere [3], these different versions can be collectively captured by the term “ADHD/HD” [4].

ADHD/HD is a common condition, with a worldwide pooled prevalence estimated at 3.4% in child populations [5]. The population prevalence of persistent adult ADHD/HD is lower, around 2.6%, though the prevalence of symptomatic adult ADHD/HD syndrome can be higher [6].

ADHD/HD is associated with various psychiatric comorbidities, psychosocial maladjustments and increased risk of suicidality [7–9]. Around 65–89% of people with ADHD/HD have a comorbid psychiatric disorder [7, 10]. The prevalence of ADHD/HD in clinical settings is, therefore, higher than community samples, as confirmed by two recent systematic reviews reporting ADHD/HD prevalence in adult clinical settings [9, 11]. Adamis et al. [9] reported pooled prevalence of 14.6 and 26.7% in adult psychiatric outpatient clinics based on 14 studies, with the lower estimate derived from studies using a 2-stage diagnostic evaluation design and the higher estimate from rating scales. Gerhand and Saville [11] identified prevalence ranging between 6.9 and 38.75% in adult psychiatric settings, based on 15 studies. Large discrepancies in reported estimates from both reviews could arise from the small number of studies examined.

Providing more accurate estimates of ADHD prevalence in clinical settings is essential for three reasons. First, ADHD/HD causes functional, emotional, social, academic and occupational impairments, and increases the risks of other psychiatric conditions, including depression, anxiety, conduct problems, personality difficulties, substance use, behavioral addiction and gaming disorder [7, 10]. Second, due to these associations, individuals are more likely to attend mental health services for treatment of their comorbid conditions rather than their ADHD, whilst ADHD symptoms are often misattributed to other conditions [12]. Importantly, treatment of comorbid conditions is less effective when the primary ADHD remains untreated [12]. Conversely, co-treatment of both ADHD and comorbid conditions significantly improves outcomes versus treating comorbid conditions alone [13]. Third, there is increasing interest in the role of ADHD/HD in the risks of suicide attempts and completed suicides, and how treating ADHD lowers these risks [7, 14, 15]. ADHD/HD elevates suicide risk by at least two pathways. Firstly, by inherently elevated impulsivity: ADHD/HD by itself increases the risk of suicide attempts three-fold, and completed suicide five-fold, after adjusting for comorbid psychiatric disorders [16]. Secondly, by comorbidity association, i.e. depression, bipolar disorder, and personality disorders, all of which are independent risk factors for suicidality [17–19]. Recent studies [20, 21] reported a risk reduction in suicide attempts by 24–31% from ADHD medication intervention, rendering it critical to assess ADHD in clinical populations for suicide prevention.

Therefore, accurate ADHD/HD prevalence estimation within psychiatric settings can potentially improve clinical outcomes. Accurate prevalence data facilitate service planning and resource allocation, especially within acute and subacute clinical settings.

In this study, we aim to conduct a comprehensive systematic review and meta-analysis to estimate the pooled prevalence of ADHD/HD in various psychiatric settings based on the extensive literature of ADHD/HD prevalence in clinical settings. Additionally, this study is the first to systematically evaluate the effects of potential modifiers, including age, sex, time trends, geographic regions, assessment methods, and co-occurring psychiatric disorders.

## Methods

This systematic review and meta-analysis were conducted according to the Preferred Reporting Items for Systematic Reviews and Meta-Analyses (PRISMA) checklist [22], as registered with PROSPERO (CRD42021270540).

### Search strategy and selection criteria

Studies reporting the prevalence of ADHD or HD within clinical settings managing mental health conditions were included. A “clinical population” was defined as patients (i) admitted to psychiatric inpatient wards or (ii) attending general psychiatric outpatient clinics, including pediatric clinics for children, addiction facilities and subspecialist mental health clinics. We included general pediatric clinics given the frequent management of childhood ADHD/HD by pediatricians; and “Tourette’s disorder” (and “tic disorders”) given their listing under the neurodevelopmental disorders section in ICD and DSM systems. We excluded neurological or specialist clinics providing services for individuals with epilepsy, encephalitis, genetic disorders, intellectual disabilities, metabolic disorders and general medical conditions. The full list of the search words/terms and exclusion criteria are included in Supplementary Fig. 1 and Supplementary Table 1 respectively.

Prevalence was derived from prospective assessments for ADHD/HD using a validated rating scale or clinical interview or retrospectively from clinical record chart review. We excluded four broad groups of studies due to biases or incomplete information: (1) publications using methodologies including case reports, case-control studies, poster abstracts, dissertations and systematic reviews; (2) studies reporting ADHD/HD symptoms as dimensional scores where prevalence could not be determined; (3) studies describing patients recruited for clinical trials; and (4) studies with inappropriate samples, such as family studies where the relatives of those with ADHD/HD were evaluated.

Search strategies included (1) database searches, (2) reviewing reference lists from articles retrieved and from published narrative or systematic reviews, and (3) correspondence with authors of identified studies. The electronic databases CINAHL, Embase, Medline, and PsycINFO were searched for eligible full-text papers published in English since inception until 1st July 2021 (“Phase 1”) and then between 1st July 2021 and 1st August 2023 (“Phase 2”).

In Phase 1, abstract screening was aided by the machine learning-assisted tool Research Screener (*n* = 22,162 articles) for efficiency (Supplementary Fig. 2) [23]. Screening for Phase 2 was conducted manually. Duplicate records were excluded. Phase 1 titles and abstracts were screened in Research Screener by two investigators (S.J., J.D.). For Phase 2 abstracts, three investigators (E.L, B.S., W.C.) screened each title and abstract manually.

Six investigators (S.J., E.L., K.V.K., B.F., B.S., B.A.F.) worked in pairs to extract and cross-check data from full-text reviews. The data extracted included: author, publication year, study design, country, clinical setting, sub-specialty setting, age demographic, diagnostic assessment tool, diagnostic nosology (i.e., DSM or ICD), age (range and mean), and ADHD/HD prevalence data stratified by age and sex whenever available. Difficulties identifying relevant data, or any conflicts, were discussed and resolved in meetings with two senior investigators (J.D., W.C.). Prevalence data reported for different study populations (e.g., for separate countries) in the same article were reported for each study population.

Studies were classified as either pediatric or adult. “Pediatric” was defined as “up to 18 years old”. To avoid unnecessarily excluding studies with a small number of older participants, studies were included if the age spread (i.e., one standard deviation (SD) above the mean age of the study sample) was within the pediatric age range. Likewise for adults, studies were included if the age spread (i.e., one SD below the mean age of the study sample) was within the adult age range, i.e., 18 years or older.

Clinical settings were categorized as “inpatient”, “outpatient” or both combined. Pediatric subspecialty settings were categorized under: “anxiety disorders”, “autism spectrum disorders”, “bipolar disorders”, “CAMHS” (child and adolescent mental health services), “deliberate self-harm”, “eating disorders”, “first episode psychosis”, “functional disorders”, “mood disorders”, “pediatrics”, “substance use disorders”, “conduct disorder”, “internet/gaming addiction”, and “tic disorders”. Adult subspecialty settings were categorized under “adult general”, “anxiety disorders”, “autism spectrum disorders”, “bipolar disorders”, “eating disorders”, “first episode psychosis”, “gambling disorders”, “mood disorders”, “personality disorders”, “schizophrenia”, “substance use disorders” and “tic disorders”. Geographic regions were categorized as “Africa”, “Middle East”, “Europe”, “North America”, “Oceania”, “Asia” and “South America”.

### Critical appraisal

The Joanna Briggs Institute Prevalence Critical Appraisal Checklist guided assessment of the quality of included studies [24]. The quality criteria for prevalence studies corresponded with the inclusion criteria except for the sample size criterion. Assuming each pooled prevalence statistic was a true estimate of prevalence, a sample size calculation formula [25] was applied, and the proportion of studies with adequate precision was identified. Precision was defined as a 95% confidence interval within 0.05 of the estimated true prevalence.

### Data synthesis and analysis

Pooled estimates of ADHD/HD prevalence were calculated separately for pediatric and adult populations. Prevalence was estimated using the following subgroupings: sex, clinical setting, sub-specialty setting and geographic region, providing nested subgroup analyses. Due to the heterogeneity of study settings, random effects models were used to calculate the pooled estimates and their confidence intervals using the method of DerSimonian and Laird [26] implemented in the STATA function *metaprop*. To identify whether any studies had a disproportional influence on the total pooled prevalence, the leave-one-out analysis was used. Forest plots were assembled showing the pooled prevalence for each subgrouping of studies. Overall time trends in ADHD/HD prevalence were investigated using random effects meta-regression (STATA version 18 function *metareg*) with study year as the independent variable. Meta-regression analyses were performed with geographic region, sample size and year of publication as the independent variables. Heterogeneity was evaluated using the Cochran’s Q statistic. In the meta-regression, variance was evaluated using the R-squared statistic, indicating the percentage of total heterogeneity explained by the independent variable.

## Results

**Fig. 1:**
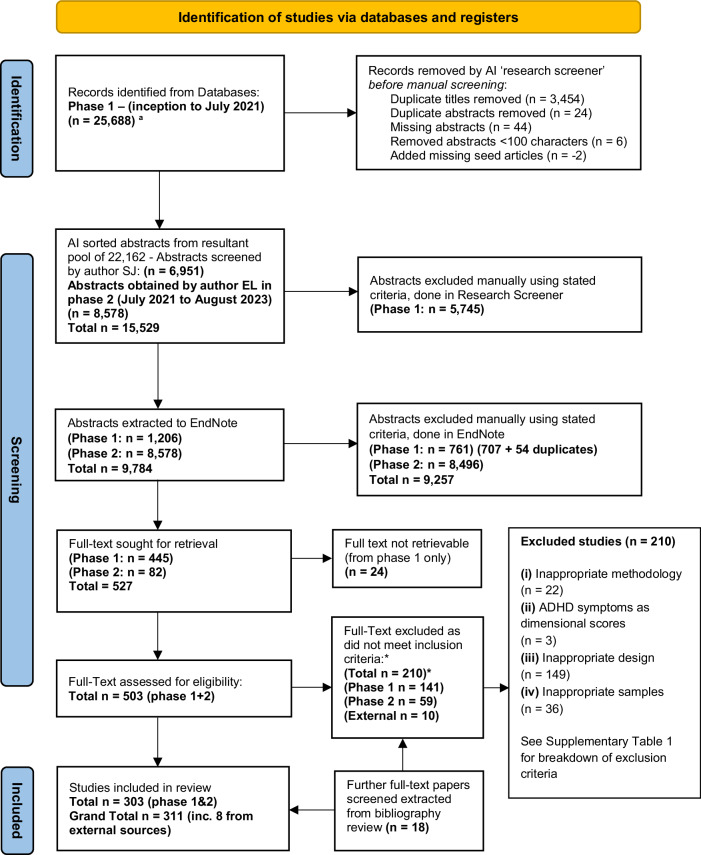
PRISMA flow diagram of study selection.

The search yielded 30,740 (Phase 1: *n* = 22,162, Phase 2: *n* = 8578) abstracts of which 527 (Phase 1: *n* = 445, Phase 2: *n* = 82) met the criteria for full-text review. Twenty-four full-text articles were not retrievable (Supplementary Fig. 3). An additional 18 articles were identified from the bibliography review. See Supplementary Fig. 4 for the full inclusion list. Where there was missing prevalence data, the corresponding author for two studies provided additional information; 210 studies were excluded and are listed in Supplementary Table 2 with exclusion criteria. In total, 311 studies were suitable for inclusion (Supplementary Fig. 5), including 351 study populations (162 adult, 189 pediatric) evaluating 653,558 pediatric and 43,311 adult participants. The study selection is presented in Fig. 1.

The median (range) sample size was 169 (7–306,861) in the pediatric studies and 162.5 (12–2163) in the adult studies. The mean (SD) of mean ages reported for pediatric studies was 11.67 (3.25) years (*n* = 132 studies); and the mean (SD) of mean ages for adult studies was 36.3 (7.6) years (*n* = 135 studies). Eighteen pediatric studies (9.5%) included a minority of participants aged over 18, but complied with our age inclusion criterion; likewise, eleven adult studies (6.8%) included a minority of participants aged under 18. Eighty percent of studies included data on sex distribution, yielding 43.6% males in pediatric samples, 58.6% males in adult samples.

The articles included were published between 1981 and 2023, spanning four decades in which two ICD classifications and six DSM editions were used to define ADHD/HD (for respective distribution see Supplementary Table 3).

### Time trend effect

**Fig. 2:**
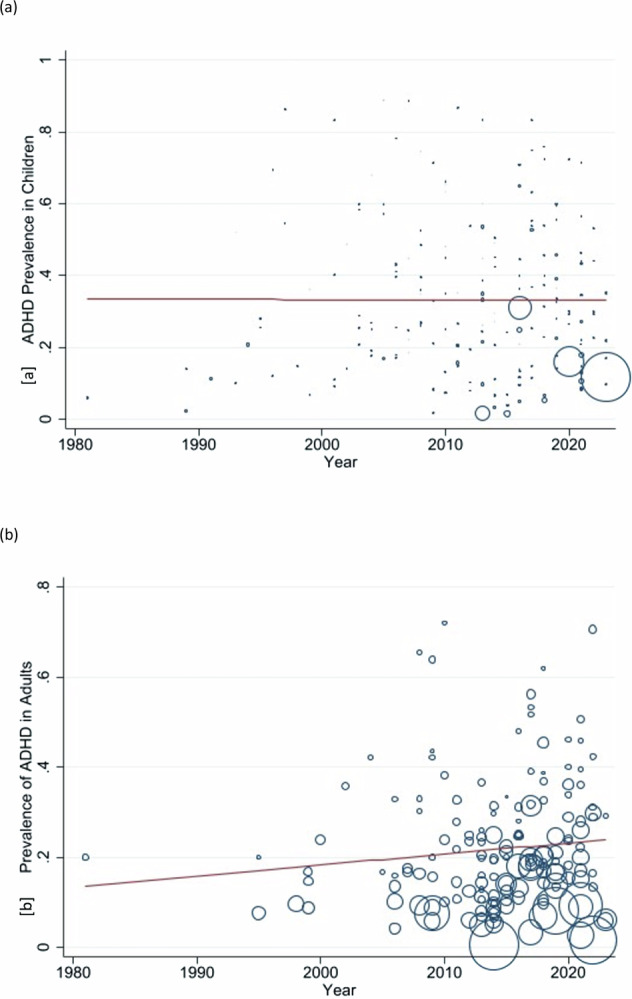
Time-trends of prevalence of ADHD/HD for pediatric and adult studies. **a** Time-trend of Clinical Prevalence of attention deficit hyperactivity disorder/hyperkinetic disorder (ADHD/HD) in pediatric studies. The size of circles represents the sample size with larger circles for larger sample sizes. **b** Time-trend of Clinical Prevalence of attention deficit hyperactivity disorder/hyperkinetic disorder (ADHD/HD) in adult studies. The size of circles represents the sample size with larger circles for larger sample sizes.

Prevalence changed by −0.00009 per year (95% CI −0.0039–0.0037; *P* = 0.97) in the pediatric studies and increased by 0.0024 per year (95% CI −0.0010–0.0058: *P* = 0.16) in adult studies, indicating that the time-trend was stable (Fig. 2a, b, Table 1). Given these small and non-significant changes in prevalence estimates over time, the prevalence studies across the whole study period could be combined. As such, these time trend tests provided the basis for prevalence estimates to be pooled across all pediatric and all adult studies.

**Table 1:** Univariate meta-regression models for pediatric and adult studies.

Pediatric Studies			
Geographic Region			
Region	Prevalence Difference	95% CI	*P* value
North America	Ref		
Africa	−0.211	−0.352, −0.070	0.003
Europe	−0.119	−0.193, −0.044	0.002
Asia	−0.136	−0.232, −0.040	0.006
Middle East	0.073	−0.021, 0.167	0.13
Oceania	−0.056	−0.225, 0.113	0.51
South America	−0.009	−0.199, 0.181	0.92
	Variance Explained	F-statistic (6182)	*P* value
Overall	14.32%	4.85	<0.001
Year			
Prevalence Change (95% CI)		t-statistic	*P* value
−.0000858 (−0.00391, 0.00374)^a^		−0.04	0.97
Sample Size			
−7.44e–7 (−1.90e–6, 4.16e–7)^a^		−1.27	0.21
Adult Studies			
Geographic Region			
Region	Prevalence Difference	95% CI	*P* value
North America	Ref		
Africa	0.047	−0.087, 0.180	0.49
Europe	0.022	−0.036, 0.081	0.45
Asia	0.030	−0.058, 0.117	0.50
Middle East	0.044	−0.035, 0.123	0.27
Oceania	−0.034	−0.236, 0.167	0.74
South America	0.035	−0.112, 0.183	0.64
	Variance Explained	F-statistic (6155)	*P* value
Overall	0.94%	0.31	0.93
Year			
Prevalence Change (95% CI)		t-statistic	*P* value
0.00266 (−0.00069, 0.00602)^a^		1.57	0.12
Sample Size			
−0.000081 (−0.000146, −0.000016)^a^		−2.46	0.015

^a^Prevalence change per unit increase in predictor variable.

### Pooled prevalence

The overall pooled prevalence of ADHD/HD in clinical settings was 32.4% (95% CI 30.7–34.0%) for pediatrics and 21.4% (95% CI 20–23%) for adults (see Tables 2 and 3). Excluding the 10 pediatric studies where the age overlapped into adulthood, the prevalence of pediatrics changed from 32.4% (95% CI 30.7–34.0) to 32.1% (95% CI 30.4–33.9). Excluding the 10 adult studies where the age overlapped into childhood, the prevalence of adults changed from 21.4% (95% CI 19.7–23.0) to 21.7% (95% CI 20.0–23.4). For these twenty studies, each was allocated to pediatric or adult categories, depending on the age range. These were two different sets of 10 studies. One pediatric study reported 100% ADHD/HD prevalence in a sample with tic disorders [27] and was excluded during meta-analysis because of computational impracticality. Using the leave-one-out sensitivity analysis for both pediatric and adult studies, no study in either dataset was identified as an outlier that would influence the reported estimates. Our analyses revealed significant heterogeneity across groups of studies (*P* < 0.001), consistent with the variation in clinical groups and settings.

**Table 2:** Pooled prevalence, confidence intervals and proportion of studies meeting the target sample size for pediatric studies.

Criteria	Number of studies	Pooled prevalence	Pooled prevalence – 95% CI	Cochran’s Q (df)	Heterogeneity *P* value	Target Sample Size^a^	No. studies meeting target sample size (%)
**SECTION 1 STUDIES**							
**All pediatric studies**	189	0.32	0.31–0.34	41878.7 (188)	<0.001	334	55
**Pediatrics – male**	44	0.38	0.34–0.42	15516.3 (43)	<0.001	362	10
Rating scales	9	0.51	0.40–0.62	260.2 (8)	<0.001		
Diagnostic interviews	22	0.37	0.27–0.46	820.5 (21)	<0.001		
Clinical records	13	0.32	0.25–0.39	13748.2 (12)	<0.001		
**Pediatrics – female**	41	0.18	0.16–0.20	3499.6 (40)	<0.001	227	12
Rating scales	9	0.42	0.27–0.57	397.4 (8)	<0.001		
Diagnostic interviews	20	0.18	0.13–0.23	264.1 (19)	<0.001		
Clinical records	12	0.16	0.14–0.19	2798.7 (11)	<0.001		
**SECTION 2 SETTINGS**							
**OUTPATIENT SETTINGS**	124	0.37	0.33–0.40	18281.8 (123)	<0.001	358	34
**Pediatrics – male**	27	0.45	0.36–0.54	2024.4 (26)	<0.001	380	4
Rating scales	9	0.51	0.40–0.62	260.2 (8)	<0.001		
Diagnostic interviews	14	0.40	0.28–0.52	663.4 (13)	<0.001		
Clinical records	4	0.46	0.20–0.72	584.2 (3)	<0.001		
**Pediatrics – female**	25	0.30	0.24–0.36	1062.6 (24)	<0.001	327	5
Rating scales	9	0.42	0.27–0.57	397.4 (8)	<0.001		
Diagnostic interviews	12	0.20	0.13–0.27	174.9 (11)	<0.001		
Clinical records	4	0.33	0.11–0.54	456.3 (3)	<0.001		
**INPATIENT SETTINGS**	44	0.20	0.18–0.22	2964.9 (43)	<0.001	246	12
**Pediatrics – male**	12	0.23	0.19–0.26	1190.3 (11)	<0.001	272	3
Rating scales	–	–	–	–	–		
Diagnostic interviews	6	0.24	0.18–0.30	11.7 (5)	0.04		
Clinical records	6	0.22	0.17–0.27	1172.8 (5)	<0.001		
**Pediatrics – female**	12	0.09	0.07–0.11	824.5 (11)	<0.001	126	7
Rating scales	–	–	–	–	–		
Diagnostic interviews	6	0.09	0.05–0.13	10.04 (5)	0.07		
Clinical records	6	0.10	0.07–0.12	802.2 (5)	<0.001		
**SECTION 3 - CONDITION**							
Anxiety disorders	9	0.25	0.17–0.33	129.2 (8)	<0.001	288	2
ASD	25	0.47	0.41–0.54	810.7 (24)	<0.001	383	3
Bipolar disorders	14	0.38	0.21–0.56	1235.1 (13)	<0.001	362	0
CAMHS	78	0.31	0.28–0.35	29638.2 (77)	<0.001	329	37
Deliberate self-harm	3	0.40	0.05–0.75	126.6 (2)	<0.001	369	1
Eating disorders	2	0.02	0.01–0.03	–	–	30	2
First episode psychosis	3	0.42	0.13–0.71	83.3 (2)	<0.001	374	1
Functional disorders	5	0.17	0.08–0.27	27.0 (4)	<0.001	217	0
Mood disorders	6	0.28	0.17–0.40	71.3 (5)	<0.001	310	1
Pediatrics	11	0.13	0.05–0.21	2209.6 (10)	<0.001	174	10
Substance use disorders	15	0.23	0.18–0.28	246.3 (14)	<0.001	272	4
Tic disorders	11	0.42	0.33–0.51	94.9 (10)	<0.001	374	0
Conduct Disorder	4	0.58	0.26–0.90	55.3 (3)	<0.001	374	0
Internet/Gaming Addiction	2	0.67	0.61–0.73	–	–	340	0
**SECTION 4 – GEO-REGION**							
Africa	9	0.17	0.10–0.24	289.1 (8)	<0.001		
Middle East	26	0.46	0.37–0.54	1267.8 (25)	<0.001		
Europe	49	0.26	0.22–0.31	5111.5 (48)	<0.001		
North America	70	0.38	0.35–0.40	27979.9 (69)	<0.001		
Oceania	6	0.33	0.18–0.47	1038.9 (5)	<0.001		
Asia	24	0.24	0.18–0.30	2352.4 (23)	<0.001		
South America	5	0.37	0.21–0.52	64.7 (4)	<0.001		

We grouped the estimates under 4 sections. The first section includes the overall estimates, and those stratified by sex and three evaluation/diagnostic methods. The second section includes different clinical settings. The third section includes different comorbidity condition specialist clinics. And the fourth second includes different geo-regions.

^a^We calculated the target sample size post-hoc based on the empirical data ascertained in our study. The target sample size was estimated, and we then provided the number of studies in that group that met the estimated target sample size in the final column. Target sample size estimated with the formula Z^2^P(1-P)/d^2^, where Z = 1.96, P is the pooled prevalence which was assumed to be the closest to the expected prevalence, and d was 5% equating to a CI width of 10% which is a stringent estimate of precision or sampling error [24].

**Table 3:** Pooled prevalence, confidence intervals and proportion of studies meeting the target sample size for adult studies.

Criteria	Number of studies	Pooled prevalence	Pooled prevalence – 95% CI	Cochran’s Q (df)	Heterogeneity *P* value	Target Sample Size^a^	No. studies meeting target sample size (%)
**SECTION 1 STUDIES**							
**All adult studies**	162	0.21	0.20–0.23	4491.9 (161)	<0.001	255	41 (25.3)
**Adults – male**	61	0.25	0.22–0.28	1014.7 (60)	<0.001	288	10 (16.3)
Rating scales	24	0.30	0.25–0.35	233.5 (23)	<0.001		
Diagnostic interviews	36	0.22	0.18–0.25	413.7 (35)	<0.001		
Clinical records	1	0.09	0.07–0.10	–	–		
**Adults – female**	58	0.21	0.18–0.24	678.0 (57)	<0.001	255	8 (13.8)
Rating scales	22	0.27	0.22–0.32	189.0 (21)	<0.001		
Diagnostic interviews	35	0.17	0.14–0.20	234.6 (34)	<0.001		
Clinical records	1	0.07	0.05–0.09	–	–		
**SECTION 2 SETTINGS**							
**OUTPATIENT SETTINGS**	92	0.21	0.19–0.23	2731.1 (91)	<0.001	255	24 (26.1)
**Adults – male**	37	0.24	0.20–0.28	617.0 (36)	<0.001	280	6 (16.2)
Rating scales	12	0.30	0.21–0.38	112.9 (11)	<0.001		
Diagnostic interviews	25	0.21	0.17–0.25	293.0 (24)	<0.001		
Clinical records	–	–	–	–	–		
**Adults – female**	37	0.21	0.18–0.25	498.2 (36)	<0.001	255	7 (18.9)
Rating scales	14	0.26	0.20–0.33	170.0 (13)	<0.001		
Diagnostic interviews	23	0.18	0.14–0.22	196.2 (22)	<0.001		
Clinical records	–	–	–	–	–		
**INPATIENT SETTINGS**	55	0.23	0.20–0.26	1304.2 (54)	<0.001	272	15 (27.3)
**Adults – male**	19	0.28	0.22–0.34	370.3 (18)	<0.001	310	3 (15.8)
Rating scales	11	0.30	0.24–0.37	102.6 (10)	<0.001		
Diagnostic interviews	7	0.26	0.16–0.37	58.5 (6)	<0.001		
Clinical records	1	0.09	0.07–0.10	–	–		
**Adults – female**	15	0.22	0.16–0.27	133.7 (14)	<0.001	264	1 (6.7)
Rating scales	8	0.28	0.22–0.35	16.5 (7)	0.02		
Diagnostic interviews	6	0.16	0.09–0.24	33.0 (5)	<0.001		
Clinical records	1	0.07	0.05–0.09	–	–		
**SECTION 3 - CONDITION**							
Adult general	33	0.17	0.14–0.20	1147.8 (32)	<0.001	217	19 (57.8)
Anxiety disorders	6	0.19	0.11–0.26	50.9 (5)	<0.001	236	1 (16.7)
ASD	5	0.36	0.30–0.41	4.87 (4)	0.30	354	0
Bipolar disorder	17	0.23	0.16–0.30	354.8 (16)	<0.001	272	2 (11.8)
Eating disorders	7	0.24	0.12–0.36	186.7 (6)	<0.001	280	1 (14.3)
First episode psychosis	2	0.07	0.06–0.08	–	–	100	2 (100)
Gambling disorders	7	0.22	0.12–0.31	92.8 (6)	<0.001	264	1 (14.3)
Mood disorders	7	0.14	0.08–0.21	72.6 (6)	<0.001	185	2 (28.6)
Personality disorders	3	0.39	0.26–0.52	20.1 (2)	<0.001	366	0
Schizophrenia	1	0.10	0.03–0.23	–	–	138	0
Substance use disorders	69	0.22	0.20–0.25	1489.3 (68)	<0.001	264	16 (23.2)
Tic disorders	2	0.34	0.28–0.40	–	–	345	0
**SECTION 4 – GEO-REGION**							
Africa	5	0.24	0.13–0.36	109.4 (4)	<0.001		
Middle East	21	0.24	0.18–0.30	379.6 (20)	<0.001		
Europe	83	0.21	0.19–0.24	2230.2 (82)	<0.001		
North America	32	0.19	0.16–0.21	535.7 (31)	<0.001		
Oceania	2	0.14	0.10–0.17	–	–		
Asia	15	0.22	0.15–0.29	839.9 (14)	<0.001		
South America	4	0.23	0.07–0.39	138.4 (3)	<0.001		

We grouped the estimates under 4 sections. The first section includes the overall estimates, and those stratified by sex and three evaluation/diagnostic methods. The second section includes different clinical settings. The third section includes different comorbidity condition specialist clinics. And the fourth second includes different geo-regions.

^a^We calculated the target sample size post-hoc based on the empirical data ascertained in our study. The target sample size was estimated and we then provided the number of studies in that group that met the estimated target sample size in the final column. Target sample size estimated with the formula Z^2^P(1-P)/d^2^, where Z = 1.96, P is the pooled prevalence which was assumed to be the closest to the expected prevalence, and d was 5% equating to a CI width of 10% which is a stringent estimate of precision or sampling error [24].

### Analysis for subgroups

#### Sex

Nearly a quarter of pediatric studies (male = 25% (*n* = 45); females = 22% (*n* = 42)) and just over a third of adult studies (male = 38% (*n* = 61); female = 38% (*n* = 61)) provided sex-specific prevalence data. Forty-one pediatric studies reported both male and female data, while three reported male-only data and none reported female only data.

**Fig. 3:**
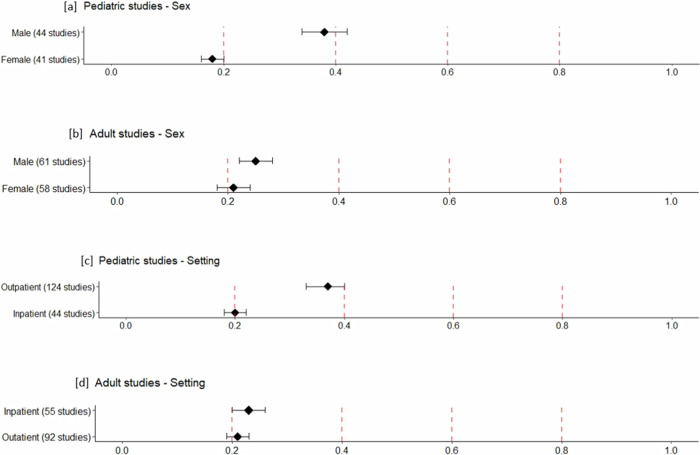
Pooled prevalence of ADHD/HD by sex and clinical setting, in pediatric and adult studies. **a** Pooled prevalence of ADHD/HD by sex in pediatric studies (*P* < 0.001). **b** Pooled prevalence of ADHD/HD by sex in adult studies (*P* = 0.072). **c** Pooled prevalence of ADHD/HD by clinical settings in pediatric studies (*P* < 0.001). **d** Pooled prevalence of ADHD/HD by clinical settings in adult studies (*P* = 0.279).

For pediatrics, prevalence was higher for males than females (38%, 95% CI 34–42% vs 18%, 95% CI 16–20%, *P* < 0.001). For adults, the prevalence was not different for males and females (25%, 95% CI 22–28% vs 21%, 95% CI 18–24%, *P* = 0.072). Figure 3a and b illustrate the pooled prevalence for sex.

#### Diagnostic methods and clinical settings

Overall, rating scales yielded a higher prevalence, followed by diagnostic interviews, with clinical record reviews identifying similar or lower prevalence (Table 2, Table 3).

Pooled prevalence based on rating scales (26% adult, 40% pediatric) were higher than validated diagnostic interviews (19% adult, 35% pediatric) or clinical record review (16% adult, 26% pediatric). The differences were observed in both males and females.

For pediatric studies, the prevalence was higher in outpatient settings compared to inpatient settings (37%, 95% CI 33–40% vs 20%, 95% CI 18–22%, *P* < 0.001) (Table 2). In adults, prevalence in outpatient settings resembled inpatient settings (21%, 95% CI 19–23% vs 23%, 95% CI 20–26%, *P* = 0.279) (Table 3). Figure 3c and d illustrate the different pooled prevalence (Fig. 3).

#### Comorbidities

ADHD/HD prevalence varied markedly across different subspecialty settings for comorbid psychiatric disorders (Tables 2 and 3 and Supplementary Fig. 6). Marked differences in prevalence across subspecialties were observed in both pediatric and adult clinical settings.

#### Geographic regions

Prevalence across different geographic regions is presented in Supplementary Fig. 7 and detailed in Tables 2 and 3. High pooled prevalence of ADHD/HD in pediatrics was found in studies from the Middle East (46%), North America (38%), South America (37%) and Oceania (33%); high pooled prevalence of ADHD/HD in adults was found in studies from Africa (24%), the Middle East (24%) and Asia (22%).

#### Meta-regression

Meta-regression was conducted with geographic region, sample size and year of publication, because these variables were fully represented in the dataset.

In pediatric studies, geographic region influenced ADHD/HD prevalence, with lower prevalence in Africa and Europe compared with North America (*P* = 0.0001), where year of publication and sample size had non-significant effects (Table 1).

In adult studies, there was little influence of geographic region and year of publication, but larger sample sizes were significantly associated with lower prevalence estimates (*P* = 0.015).

#### Heterogeneity evaluation and publication bias

First, large Cochrane’s Q values are reported in Tables 2 and 3, indicating substantial heterogeneity of studies. Second, a systematic effect of sample size on the pooled prevalences by geographic region, year of publication and sample size was identified in the meta-regression (Table 1). Funnel plots for pediatric and adult studies are presented in the Supplementary Materials, which indicated higher standard errors in studies with smaller sample sizes reporting higher prevalence in both pediatric and adult studies (Supplementary Fig. 8).

## Discussion

Our findings provide the most robust evidence-based estimates of the prevalence of ADHD/HD in clinical settings, based on a comprehensive systematic review of published literature. Moreover, we dissected differential prevalence estimates based on potential modifiers. We also conducted meta-regression to evaluate the effects of geographic regions, year of publication and sample size.

There are six key findings. First, no time-trend effects were detected within our study period. Second, the pooled prevalence of ADHD/HD in clinical settings was 32% for pediatrics and 21% for adults. For pediatrics, the male to female ratio for ADHD/HD was 1.9:1. For adults, the ratio is smaller at 1.2:1. Third, prevalence was higher in the outpatient setting (37% pediatric, 21% adult) than in the inpatient setting (20% pediatric, 23% adult). Fourth, studies based only on rating scales reported higher prevalence than those using research diagnostic interviews or clinical diagnoses derived from records. Notably, rating scales likely over-diagnose ADHD because they do not fully attend to DSM criteria for age at onset, impairment and pervasiveness across settings. Our findings provide further evidence that rating scales should not be used as the sole tool for ADHD diagnosis in routine clinical practice. Moreover, conflating ADHD with other psychiatric conditions associated with inattention, hyperactivity and impulsivity symptoms can potentially lead to diagnostic misclassification, inflating ADHD prevalence in clinical settings, although some works show that much of ADHD’s comorbidity with other disorders cannot be explained by overlapping symptoms [28]. This is most likely to arise if only rating scales were used for assessment. Therefore, diagnostic interviews by experienced clinicians are critical and can differentiate ADHD from mimicking conditions, by (i) applying operationalized authoritative diagnostic criteria, (ii) taking a full developmental history, (iii) adhering to DSM-5-TR diagnostic criterion E, and (iv) conducting therapeutic trials of medications, if still in doubt. For our findings, we suggest that the pooled prevalence estimates derived from diagnostic interviews to be likely most representative of the true estimates of ADHD prevalence in clinical settings (i.e., 0.35 (95% CI 0.31–0.39) for pediatrics; and 0.19 (95% CI 0.17–0.21) for adults).

Fifth, ADHD/HD prevalence varied across subspecialist settings defined by comorbid conditions. Notably for all ages, the highest prevalence of ADHD/HD was reported from subspecialist clinics focused on ASD, bipolar disorder and tic disorders. In children, high prevalence was also detected for internet/gaming addiction (67%), conduct disorder (58%), first-episode psychosis (42%) and DSH (40%) conditions. In adults, high prevalence was also detected for personality disorder (39%) and eating disorder (24%). Finally, geographic regions showed greater prevalence differences in children than adults, with Africa, Europe and Asia reporting a lower prevalence in children versus other regions.

Our findings reflect comorbidity data derived from cross-sectional clinical surveys and population studies. Kessler et al. [29] conducted a survey on the correlates of adult ADHD using a nationally representative sample and identified similar (or lower) prevalence of adult ADHD amongst different disorders: 13% ADHD in any mood disorder (cf. our finding of 14%), 9.5% in any anxiety disorder (cf. our finding of 19%), and 11% in any substance use disorder (cf. our finding of 22%). They also found a high prevalence of comorbidities in those with adult ADHD: 38% of individuals with ADHD had any mood disorder, 47% had any anxiety disorder, and 15% had any substance use disorder. A meta-analysis estimated the pooled ADHD prevalence in substance use disorder at 23%, mirroring our finding of 22% in adults [30]. Sobanski et al. [31] reported high prevalence of comorbidities in children with ADHD, especially those with emotional lability: 22% had depression, 44% had anxiety disorder, and 4% had substance use disorder. A recent meta-analysis on ADHD prevalence in ASD populations provided pooled estimates of 48% in pediatric and 22% in adult populations [32]. Our pooled estimates were 47% in pediatric and 36% in adult ASD populations. Overall, there are high levels of comorbidities in ADHD participants of both adult and pediatric studies, in line with our findings.

There were three unexpected findings. First, a six-fold discrepancy for “first episode psychosis” between pediatric (42%) and adult (7%) populations. We postulate that this discrepancy may arise from either (i) under-detection of ADHD in adult populations, or (ii) ADHD-related drug induced psychosis being more common in the youth populations thereby inflating ADHD prevalence due to multimorbidity, or (iii) both. Our findings highlight the detected discrepancy, and future studies can specifically test these hypotheses with suitable designs. Second, ADHD/HD prevalence in eating disorder populations also differed widely between children (2%) and adults (24%). ADHD/HD is associated with binge-eating disorder (BED) - relatively uncommon in children, with a pooled prevalence around 1.3% [33], and relatively more common in adults, around 6.6% [34]. The ten-fold discrepancy could arise from greater diagnostic overshadowing and information ascertainment bias in pediatric eating disorder clinics. Third, we identified greater geographic variation in the pediatric populations (range 15–41%) than adult populations (range 14–24%). This may arise from greater differences in clinical referral bias or in diagnostic practices of ADHD/HD in children across regions, with global resource shortages of child mental health services [35] especially in low and middle-income countries [36]. In contrast, the prevalence in adult clinical settings varied less, more in line with the variation in population prevalence identified by Polanczyk et al. [4], who concluded their detected variation arose from methodological differences rather than true geographic variations. Overall, our findings confirm previous research that ADHD/HD is not just a largely American disorder [37].

A strength of our study is its comprehensive range and number of studies examined, with the evaluation of potential modifiers. Several limitations of our study should be considered. First, where diagnosis was ascertained from clinical records, diagnostic methods were not detailed and are therefore unknown; hence, unmeasured variations and confounders may distort our estimates. The relatively lower prevalence in adult studies based on clinical records may also reflect the underdiagnosis of adults with ADHD in clinical settings reported in the literature [13], and our pooled estimate of this (7% for females and 9% for males) should not be taken as the true prevalence. Second, some studies relied on self-report or carer-report screening tools (based on responders’ subjective impression), which were then dichotomized to yield prevalence estimates, rather than based on clinical or validated research diagnostic interviews. Overall pooled prevalence could be inflated in both pediatrics and adults by studies based on rating scales. Third, some studies examined a sub-population within psychiatric settings, e.g., inpatients with depression, amplifying the confounding effects of referral bias inherent in clinical samples. Fourth, publication bias may be introduced by our exclusion of conference abstracts and posters, which may contain more recent prevalence data. Fifth, additional bias could be introduced into our geographic analyses by excluding 361 non-English-language papers. Yet, comprehensive translation of studies published in local languages may introduce unwanted variation in the quality of studies, and this undertaking is also outside the resources available for this study. Sixth, large Cochrane’s Q values were found in our pool estimates suggesting substantial heterogeneity across the samples in the meta-analyses. Regarding the seventh limitation, we excluded neurological or specialist clinics providing services for individuals with epilepsy, encephalitis, genetic disorders, intellectual disabilities, metabolic disorders and general medical conditions for two reasons. First is the issue of phenocopy conditions mimicking ADHD, as some genetic and neurological disorders (such as head injuries or hypoxia arising from epileptic seizures) can give rise to inattention, hyperactivity and impulsivity due to brain changes, mimicking ADHD and potentially leading to misdiagnosis. Second, the diagnosing of ADHD in the populations with intellectual disabilities is potentially complex. Intellectual disabilities can lead to diagnostic inaccuracy due to (i) diagnostic overshadowing (i.e., misattributing ADHD symptoms as a part of ID), or (ii) misclassification by inexperienced clinicians or researchers, as the inattention, hyperactivity or impulsivity could arise from lower mental age (i.e., symptoms in keeping with lower mental age, but not due to ADHD per se), and such a diagnosis requires highly specialized clinicians who can adjust symptom levels according to mental age. Intellectual disability cases are also more common in clinics with epilepsy, encephalitis, genetic disorders, metabolic disorders and general medical conditions. For these two reasons, we have excluded these clinics, to minimize the risk of biased estimates. Eighth, one pediatric study reported 100% ADHD/HD prevalence in a sample with tic disorders and was excluded during meta-analysis, not because of our subjective judgment that it is an outlier; but because it is mathematically impossible to include an estimate of 100% in pooled prevalence calculation.

Finally, we estimated the roles of sample size, year of publication and geographic region in meta-regression. Relatively few studies met our criterion of “adequate” sample size, noting that we adopted a very stringent requirement for this evaluation. The significant relationship between sample size and prevalence in the adult studies (where larger sample sizes yielded lower prevalence estimates) calls for caution when interpreting higher prevalence estimates of ADHD reported in small studies. We chose a random effects meta-analysis because of the variation in settings, as the smaller studies would have contributed higher weightings than those with a fixed effects meta-analysis.

### Conclusion and clinical implications

This review underscores the high prevalence of ADHD/HD across various clinical settings in both pediatric and adult populations. Our estimates of pooled prevalence (32.4% pediatrics, 21.4% adults) are 8 to 9-fold higher than the population prevalence of ADHD/HD. Given that ADHD/HD places patients at risk for many adverse outcomes, our findings underline the critical importance of resource allocation for screening, diagnosing and treating these patients in acute and subacute clinical settings. Our findings provide important prevalence data that can inform clinicians and policy makers to plan for adequate service provision.

The study by Kessler et al. [29] highlighted the under-detection and under-treatment of ADHD experienced by their study participants, despite many receiving treatments for other comorbid disorders.

Our findings can raise awareness amongst mental health clinicians regarding underlying, untreated ADHD/HD, which can drive the acute risks and more florid psychiatric symptoms. Different subtypes of clinical services can provide more rigorous screening for ADHD/HD.

## Supplementary information

Supplementary Material: Johnson et al. 2025
