# Supplementary material for: Prevalence of attention deficit hyperactivity disorder/hyperkinetic disorder of pediatric and adult populations in clinical settings: a systematic review, meta-analysis and meta-regression
Source: Mol Psychiatry. 2025 Aug 28;31(1):576–86. doi: 10.1038/s41380-025-03178-8 (PMC12700782; doi:10.1038/s41380-025-03178-8)

**Supplementary Material: Johnson et al. 2025 - Prevalence of Attention Deficit Hyperactivity Disorder/Hyperkinetic Disorder of Pediatric and Adult Populations in Clinical Settings: A Systematic Review, Meta-Analysis and Meta-Regression**

**Supplementary Figure 1:** Search terms used in the CINAHL, Embase, Medline, and PsycINFO databases to identify relevant prevalence papers.

| **1. Mental health terms**  attention deficit hyperactivity disorder or ADHD or AD/HD or attention deficit/hyperactivity disorder or attention-deficit/hyperactivity disorder or attention deficit disorder or attention-deficit disorder or ADD or attention deficit disorder with hyperactivity or ADDH or ADD/HD or ADD-HD or attention deficit or hyperactiv* or hyperactivity disorder or hyperactive child syndrome or overactive* or inattent* or impulsiv* or inattent* or sluggish cognitive tempo or externalising disorder or externalizing disorder or externali* or hyperkinetic disorder or hyperkinetic syndrome or hyperkinesis or hyperkin* or minimal brain dysfunction or MBD or mental health or mental ill* or mental disorder* or psychiatric disorder* or challenging behavio* |
| --- |
| **2. Prevalence terms**  Prevalence or rate or diagno* or systematic review or frequen* or ratio or inciden* or epidemiolog* |
| **3. Settings**  Inpatient* or outpatient* or patient or clinic* or psychiatri*or case |

Other inclusions: English language, peer reviewed (including books and book sections).

**Supplementary Table 1: Exclusion Criteria**

**Categories: (i) Inappropriate Methodology, (ii) ADHD symptoms as dimensional scores, (iii) Inappropriate design, (iv) Inappropriate samples**

| 1. **Inappropriate Methodology** |
| --- |
| 1.1 - Narrative reviews |
| 1.2 - Meta-analyses & systematic reviews |
| 1.3 - Dissertations |
| 1.4 - Letters to the editor |
| 1.5 - Abstract Only Texts |
| 1.6 - Conference Poster Presentations |
| 1.7 - Textbook articles |
| 1.8 – Foreign language only |
|  |
| **(ii) ADHD symptoms as dimensional scores** |
| 2.1 - Studies reporting dimensional scores / symptoms relating to ADHD, but without providing prevalence data |
|  |
| **(iii) Inappropriate design** |
| 3.1 - Patients as part of a clinical-trial or intervention trial study |
| 3.2 - Case report series & single case studies |
| 3.3 – Epidemiological studies / participants recruited from the community or population samples |
| 3.4 - Data based on prescriptions of medications/stimulants, rather than ADHD diagnosis |
| 3.5 - Surveys of clinicians asking them for estimates of how often they encountered ADHD |
| 3.6 - Cohort studies evaluating change in ADHD prevalence over time |
| 3.7 - Studies that evaluated lifetime/childhood prevalence, rather than current prevalence of ADHD |
| 3.8 - Studies with samples consisting of those with ADHD or a specific “assessment for ADHD clinic” – due to referral and ascertainment bias |
| 3.9 - Studies that evaluated % of attendances being due to ADHD, rather than true prevalence |
| 3.10 - Studies reporting patient populations on medication X, where Y% have ADHD |
| 3.11 – Studies excluded those with a pre-existing ADHD diagnosis |
| 3.12 - Studies obtaining kappa coefficients by comparing diagnostic tools – lacking clarity which was the gold-standard diagnostic tool |
| 3.13 – Utilised previously used sample in another study, i.e., “duplicate sample” |
| 3.14 – Study did not provide any (or sufficiently specific) ADHD prevalence data, e.g. combining ADHD and conduct disorder or no data available for individual countries in multi-centre studies |
| 3.15 – Data sourced from health fund / insurance claims, not clinical settings |
| 3.16 – Data based on sample prescribed medication X, rather than a clinical inpatient/outpatient sample |
| **(iv) Inappropriate samples** |
| 4.1 - School and university populations |
| 4.2 - General/Specialist medical or surgical wards or clinics / emergency departments - with exception of neurology clinics which included and evaluated F-code diagnoses, and general paediatric clinics where ADHD is managed |
| 4.3 - Forensic unit, prison & juvenile detention settings |
| 4.4 - General practice / primary care |
| 4.5 - Military hospitals and military service medicals |
| 4.6 - Populations of intellectual disability & fetal alcohol spectrum disorder (FASD) |
| 4.7 - Populations of offspring to parent with a mental health diagnosis |
| 4.8 - Populations with congenital malformations |
| 4.9 - Populations of low birthweight / pre-term babies |
| 4.10 - Populations who were family members of those with existing ADHD |
| 4.11 – Emergency Departments |
| 4.12 – Did not differentiate adult and paediatric samples, i.e., “all ages” articles |
| 4.13 – Head injury populations |
| 4.14 – Foster home / child welfare services populations |
| 4.15 – Samples sourced from hospital genetic databases |

**Supplementary Figure 2: Research Screener Info**

| (https://researchscreener.com) |
| --- |
| *Research Screener* learns the abstract content from a set of seed articles and generates an algorithm, ranking articles by relevance. Groups of 50 articles are first presented to the researcher for screening; papers selected by the researcher are used for ongoing machine training of the algorithm and continual re-ranking of papers by relevance. In total, 139 rounds of 50 articles were screened in Phase 1. Screening ceased when the investigators found no papers judged to be relevant in the final five rounds., i.e., no articles were deemed relevant in the last five rounds. |

**Supplementary Figure 3: 24 Papers not findable**

| Aksoy UM, Aksoy SG, Akpinar A, Maner F. Attention deficit hyperactivity disorder (ADHD) symptoms and adult ADHD diagnosis in adult men with cannabis dependence. HealthMED. 2012;6(6):1925-9. |
| --- |
| Behdani F, Hebrani P, Toufani H, Akhavan Rezayat A, Akhavan Rezayat A. Frequency of attention-deficit hyperactivity disorder in substance-dependent patients hospitalised in Mashhad Ebn-e-Sina hospital. Mental Health and Substance Use. 2011;4(4):293-301. |
| Cantwell DP, Baker L. Psychiatric and learning disorders in children with speech and language disorders: A descriptive analysis. Advances in Learning & Behavioral Disabilities. 1985;4:29-47. |
| Ceraudo G, Toni C, Vannucchi G, Rizzato S, Casalini F, Dell'Osso L, et al. Is substance use disorder with comorbid adult attention deficit hyperactivity disorder and bipolar disorder a distinct clinical phenotype? Heroin Addiction and Related Clinical Problems. 2012;14(3):71-6. |
| Clark DB, Pollock N, Bukstein OG, Mezzich AC, Bromberger JT, Donovan JE. Gender and comorbid psychopathology in adolescents with alcohol dependence. Journal of the American Academy of Child and Adolescent Psychiatry. 1997;36(9):1195-203. |
| Gordon-Lipkin E, Marvin AR, Law J, Lipkin PH. Anxiety and mood disorder in children with autism spectrum disorder and ADHD. Pediatrics. 2018;141(4):1-8. |
| Horton AM, Fiscella R, Schwartz N, Anilane J, et al. Diagnosis of attention deficit disorder in adult alcoholics: The case of DSM III-R. Psychotherapy in Private Practice. 1990;8(3):123-7. |
| Icick R, Moggi F, Slobodin O, Dom G, Mathys F, Van Den Brink W, et al. Attention Deficit/Hyperactivity Disorder and Global Severity Profiles in Treatment-Seeking Patients with Substance Use Disorders. European Addiction Research. 2020;26(4-5):201-10. |
| James PD, Smyth BP, Apantaku-Olajide T. Substance use and psychiatric disorders in Irish adolescents: A cross-sectional study of patients attending substance abuse treatment service. Mental Health and Substance Use: Dual Diagnosis. 2013;6(2):124-32. |
| Lencz T, Smith CW, Auther A, Correll CU, Cornblatt B. Nonspecific and attenuated negative symptoms in patients at clinical high-risk for schizophrenia. Schizophrenia Research. 2004;68(1):37-48. |
| Lumley VA, McNeil CB, Herschell AD, Bahl AB. An examination of gender differences among young children with disruptive behavior disorders. Child Study Journal. 2002;32(2):89-100. |
| McAweeney M, Rogers NL, Huddleston C, Moore D, Gentile JP. Symptom prevalence of ADHD in a community residential substance abuse treatment program. Journal of Attention Disorders. 2010;13(6):601-8. |
| McIntyre RS, Kennedy SH, Soczynska JK, Nguyen HTT, Bilkey TS, Woldeyohannes HO, et al. Attention-deficit/hyperactivity disorder in adults with bipolar disorder or major depressive disorder: Results from the international mood disorders collaborative project. Primary Care Companion to the Journal of Clinical Psychiatry. 2010;12(3). |
| Mol Debes NMM, Hjalgrim H, Skov L. Validation of the presence of comorbidities in a danish clinical cohort of children with Tourette syndrome. Journal of Child Neurology. 2008;23(9):1017-27. |
| Moller LR, Sorensen MJ, Thomsen PH. ICD-10 classification in Danish child and adolescent psychiatry--Have diagnoses changed after the introduction of ICD-10? Nordic Journal of Psychiatry. 2007;61(1):71-8. |
| Mortberg E, Tilfors K, Bejerot S. Screening for ADHD in an adult social phobia sample. Journal of Attention Disorders. 2012;16(8):645-9. |
| Moura HF, Faller S, Benzano D, Szobot C, Von Diemen L, Stolf AR, et al. The effects of ADHD in adult substance abusers. Journal of Addictive Diseases. 2013;32(3):252-62. |
| Peter SC, Whelan JP, Ginley MK, Pfund RA, Wilson KK, Meyers AW. Disordered gamblers with and without ADHD: The role of coping in elevated psychological distress. International Gambling Studies. 2016;16(3):455-69. |
| Petersen MC, Kube DA, Whitaker TM, Graff JC, Palmer FB. Prevalence of developmental and behavioral disorders in a pediatric hospital. Pediatrics. 2009;123(3):e490-e5. |
| Poyraz Findik OT, Ceri V, Unver H, Perdahli Fis N, Rodopman Arman A, Beser C, et al. Mental health need and psychiatric service utilization patterns of refugee children in Turkey: A comparative study. Children and Youth Services Review Vol 124 2021, ArtID 105970. 2021;124. |
| Pulsifer BH, Evans CL, Capel L, Lyons-Hunter M, Grieco JA. Cross-sectional assessment of mental health and service disparities in a high-risk community. Translational Issues in Psychological Science. 2019;5(4):365-73. |
| Sidana A, Bhatia MS, Choudhary S. Prevalence and pattern of psychiatric morbidity in children. Indian Journal of Medical Sciences. 1998;52(12):556-8. |
| Speltz ML, McClellan J, DeKlyen M, Jones K. Preschool boys with oppositional defiant disorder: clinical presentation and diagnostic change. Journal of the American Academy of Child & Adolescent Psychiatry. 1999;38(7):838-45. |
| Wright HH, Batey SR, Buttefield PT, Harris EC. The changing spectrum of children and adolescents seen in a child and adolescent psychiatric clinic during the 1970's. Psychiatric Forum. 1989;15(1):11-8. |

**Supplementary Figure 4: List of sought papers for full-text retrieval**

| **Phase 1: (n= 445)** |
| --- |
| Abel KF, Ravndal E, Clausen T, Bramness JG. Attention deficit hyperactivity disorder symptoms are common in patients in opioid maintenance treatment. European Addiction Research. 2018;23(6):298-305. |
| Abiodun OA, Tunde-Ayinmode MF, Adegunloye OA, Ayinmode BA, Sulyman D, Unaogu NN, et al. Psychiatric morbidity in paediatric primary care clinic in Ilorin, Nigeria. Journal of Tropical Pediatrics. 2011;57(3):173-8. |
| Adamis D, Graffeo I, Kumar R, Meagher D, O'Neill D, Mulligan O, et al. Screening for attention deficit-hyperactivity disorder (ADHD) symptomatology in adult mental health clinics. Irish Journal of Psychological Medicine. 2018;35(3):193-201. |
| Adler LA, Guida F, Irons S, Rotrosen J, O'Donnell K. Screening and imputed prevalence of ADHD in adult patients with comorbid substance use disorder at a residential treatment facility. Postgraduate Medicine. 2009;121(5):7-10. |
| Aksoy UM, Aksoy SG, Akpinar A, Maner F. Attention deficit hyperactivity disorder (ADHD) symptoms and adult ADHD diagnosis in adult men with cannabis dependence. HealthMED. 2012;6(6):1925-9. |
| Albanese MJ, Clodfelter RC, Jr., Pardo TB, Ghaemi S. Underdiagnosis of bipolar disorder in men with substance use disorder. Journal of Psychiatric Practice. 2006;12(2):124-7. |
| Al-Haidar FA. Co-morbidity and treatment of attention deficit hyperactivity disorder in Saudi Arabia. Eastern Mediterranean Health Journal. 2003;9(5-6):988-95. |
| Alikhani R, Tehrani-Doost M, Shahrivar Z. A five-year report on clinical and demographic characteristics of children and adolescents admitted to a major psychiatric hospital. Iranian Journal of Rehabilitation Research. 2019;17(3):253-62. |
| Almeida-Montes. ADHD Prevalence in Adult Outpatients With Nonpsychotic Psychiatric Illnesses. Journal of Attention Disorders. 2007. |
| Alpert JE, Maddocks A, Nierenberg AA, O'Sullivan R, Pava JA, Worthington JJ, 3rd, et al. Attention deficit hyperactivity disorder in childhood among adults with major depression. Psychiatry Research. 1996;62(3):213-9. |
| Al-Sharbati MM, Al-Farsi YM, Al-Sharbati ZM, Al-Sulaimani F, Ouhtit A, Al-Adawi S. Profile of Mental and Behavioral Disorders Among Preschoolers in a Tertiary Care Hospital in Oman: A Retrospective Study. Oman Medical Journal. 2016;31(5):357-64. |
| Al-Sharbati MM, Al-Hussaini AA, Antony SX. Profile of child and adolescent psychiatry in Oman. Saudi Medical Journal. 2003;24(4):391-5. |
| Al-Sharbati MM, Zaidan ZAJ, Dorvlo ASS, Al-Adawi S. Characteristics of ADHD among omani schoolchildren using DSM-IV: Descriptive study. Journal of Attention Disorders. 2011;15(2):139-46. |
| Anckarsater H, Nilsson T, Stahlberg O, Gustafson M, Saury J-M, Rastam M, Gillberg C. Prevalences and configurations of mental disorders among institutionalized adolescents. Developmental Neurorehabilitation. 2007;10(1):57-65. |
| Andersson HW, Lauvsnes ADF, Nordfjaern T. Emerging Adults in Inpatient Substance Use Treatment: A Prospective Cohort Study of Patient Characteristics and Treatment Outcomes. European Addiction Research. 2021;27(3):206-15. |
| Anholt GE, Aderka IM, van Balkom AJLM, Smit JH, Schruers K, van der Wee NJA, et al. Age of onset in obsessive–compulsive disorder: admixture analysis with a large sample. Psychological Medicine. 2014;44(1):185-94. |
| Araz Altay M, Bozatli L, Demirci Sipka B, Gorker I. Current Pattern of Psychiatric Comorbidity and Psychotropic Drug Prescription in Child and Adolescent Patients. Medicina. 2019;55(5):17. |
| Arcelus J, Vostanis P. Child psychiatric disorders among primary mental health service attenders. British Journal of General Practice. 2003;53(488):214-6. |
| Arias AJ, Gelernter J, Chan G, Weiss RD, Brady KT, Farrer L, et al. Correlates of co-occurring ADHD in drug-dependent subjects: prevalence and features of substance dependence and psychiatric disorders. Addictive Behaviors. 2008;33(9):1199-207. |
| Arican I, Bass N, Neelam K, Wolfe K, McQuillin A, Giaroli G. Prevalence of attention deficit hyperactivity disorder symptoms in patients with schizophrenia. Acta Psychiatrica Scandinavica. 2019;139(1):89-96. |
| Axelson D, Findling RL, Fristad MA, Kowatch RA, Youngstrom EA, Horwitz SM, et al. Examining the proposed disruptive mood dysregulation disorder diagnosis in children in the Longitudinal Assessment of Manic Symptoms Study. The Journal of Clinical Psychiatry. 2012;73(10):1342-50. |
| Baeza I, de la Serna E, Amoretti S, Cuesta MJ, Diaz-Caneja CM, Mezquida G, et al. Premorbid Characteristics as Predictors of Early Onset Versus Adult Onset in Patients With a First Episode of Psychosis. Journal of Clinical Psychiatry. 2021;82(6):14. |
| Balazs J, Gyori D, Horvath LO, Meszaros G, Szentivanyi D. Attention-deficit hyperactivity disorder and nonsuicidal self-injury in a clinical sample of adolescents: the role of comorbidities and gender. BMC Psychiatry. 2018;18(1):34. |
| Baruch G, Vrouva I, Fearon P. A follow-up study of characteristics of young people that dropout and continue psychotherapy: Service implications for a clinic in the community. Child and Adolescent Mental Health. 2009;14(2):69-75. |
| Bassiony MM, Salah El-Deen GM, Ameen N, Mahdy RS. Prevalence, correlates, and consequences of attention-deficit/hyperactivity disorder in a clinical sample of adults with tramadol use in Egypt. American Journal on Addictions. 2021;04:04. |
| Basu S, Isaacs AN. Profile of transcultural patients in a regional Child and Adolescent Mental Health Service in Gippsland, Australia: The need for a multidimensional understanding of the complexities. International Journal of Social Psychiatry. 2019;65(3):217-24. |
| Behdani F, Hebrani P, Toufani H, Akhavan Rezayat A, Akhavan Rezayat A. Frequency of attention-deficit hyperactivity disorder in substance-dependent patients hospitalised in Mashhad Ebn-e-Sina hospital. Mental Health and Substance Use. 2011;4(4):293-301. |
| Belirgan S, Ersoy MA, Ersoy HT. Prevalence of adult attention deficit hyperactivity disorder and comorbid axis-I disorders among first time applied cases of a general psychiatry outpatient clinic and a private psychotherapy centre. Psychiatry and Clinical Psychopharmacology. 2018;28(1):25-35. |
| Bell CC, Jackson WM, Bell BH. Misdiagnosis of African-Americans with psychiatric issues - Part i. Journal of the National Medical Association. 2015;107(3):25-34. |
| Benarous X, Renaud J, Breton JJ, Cohen D, Labelle R, Guile J-M. Are youths with disruptive mood dysregulation disorder different from youths with major depressive disorder or persistent depressive disorder? Journal of Affective Disorders. 2020;265:207-15. |
| Bener A, Dafeeah EE, Abou-Saleh MT, Bhugra D, Ventriglio A. Co-Morbidity between Major Depression and Schizophrenia: Prevalence and Clinical Characteristics. Psychiatria Danubina. 2020;32(1):78-83. |
| Benti M, Bayeta AB, Abu H. Attention Deficit/Hyperactivity Disorder and Associated Factors Among Children Attending Pediatric Outpatient Departments of West Shewa Zone Public Hospitals, Central Ethiopia. Psychology Research & Behavior Management. 2021;14:1077-90. |
| Ben-Yehuda A, Aviram S, Govezensky J, Nitzan U, Levkovitz Y, Bloch Y. Suicidal behavior in minors-Diagnostic differences between children and adolescents. Journal of Developmental and Behavioral Pediatrics. 2012;33(7):542-7. |
| Bergly TH, Somhovd MJ. The relation between ADHD medication and mild cognitive impairment, as assessed by the Montreal Cognitive Assessment (MoCA), in patients entering substance use disorder inpatient treatment. Journal of Dual Diagnosis. 2018;14(4):228-36. |
| Bernardi S, Cortesse S, Solanto M, Hollander E, Pallanti S. Bipolar disorder and comorbid attention deficit hyperactivity disorder. A distinct clinical phenotype? Clinical characteristics and temperamental traits. The World Journal of Biological Psychiatry. 2010;11(3-4):656-66. |
| Bhat BA, Hussain A, Dar MA, Dar SA, Jabeen N, Rasool S, Shafi S. The Pattern of Psychiatric Morbidity in an Outpatient Child Psychiatry Clinic: A Cross-sectional, Descriptive Study from a Tertiary Care Hospital in Kashmir, North India. Indian Journal of Psychological Medicine. 2018;40(4):349-55. |
| Bhatia MS, Nigam VR, Bohra N, Malik SC. Attention deficit disorder with hyperactivity among paediatric outpatients. Journal of Child Psychology & Psychiatry & Allied Disciplines. 1991;32(2):297-306. |
| Biederman J, Faraone SV, Wozniak J, Mick E, Kwon A, Cayton GA, Clark SV. Clinical correlates of bipolar disorder in a large, referred sample of children and adolescents. Journal of Psychiatric Research. 2005;39(6):611-22. |
| Biederman J, Petty C, Faraone SV, Seidman L. Phenomenology of childhood psychosis: findings from a large sample of psychiatrically referred youth. Journal of Nervous & Mental Disease. 2004;192(9):607-14. |
| Bitter I, Mohr P, Balogh L, Latalova K, Kakuszi B, Stopkova P, et al. ADHD: a hidden comorbidity in adult psychiatric patients. Attention Deficit and Hyperactivity Disorders. 2019;11(1):83-9. |
| Blanco-Vieira T, Santos M, Ferrao YA, Torres AR, Miguel EC, Bloch MH, et al. The impact of attention deficit hyperactivity disorder in obsessive-compulsive disorder subjects. Depression & Anxiety. 2019;36(6):533-42. |
| Bolstad I, Lien L, Bramness JG. ADHD symptoms as risk factor for PTSD in inpatients treated for alcohol use disorder. Psychiatry Research. 2021;300:113904. |
| Borchardt CM, Bernstein GA. Comorbid disorders in hospitalized bipolar adolescents compared with unipolar depressed adolescents. Child Psychiatry and Human Development. 1995;26(1):11-8. |
| Bosgelmez S, Tufan AE. The prevalence of symptoms and diagnosis of attention-deficit-hyperactivity disorder among adult female patients with mild / moderate major depressive disorder who were referred to the psychiatric outpatient department of a training hospital: A preliminary study. Dusunen Adam. 2014;27(1):27-33. |
| Bozkurt H, Coskun M, Ayaydin H, Adak I, Zoroglu SS. Prevalence and patterns of psychiatric disorders in referred adolescents with Internet addiction. Psychiatry & Clinical Neurosciences. 2013;67(5):352-9. |
| Bozkurt M, Evren C, Umut G, Evren B. Relationship of attention-deficit/hyperactivity disorder symptom severity with severity of alcohol-related problems in a sample of inpatients with alcohol use disorder. Neuropsychiatric Disease & Treatment. 2016;12:1661-7. |
| Bryson SA, Corrigan SK, McDonald TP, Holmes C. Characteristics of children with autism spectrum disorders who received services through community mental health centers. Autism. 2008;12(1):65-82. |
| Budman CL, Bruun RD, Park KS, Olson ME. Rage attacks in children and adolescents with Tourette's disorder: a pilot study. Journal of Clinical Psychiatry. 1998;59(11):576-80. |
| Burket RC, Myers WC. Axis I and personality comorbidity in adolescents with conduct disorder. Bulletin of the American Academy of Psychiatry & the Law. 1995;23(1):73-82. |
| Butler SF, Arredondo DE, McCloskey V. Affective comorbidity in children and adolescents with attention deficit hyperactivity disorder. Annals of Clinical Psychiatry. 1995;7(2):51-5. |
| Cantwell DP, Baker L. Psychiatric and learning disorders in children with speech and language disorders: A descriptive analysis. Advances in Learning & Behavioral Disabilities. 1985;4:29-47. |
| Carballal Marino M, Gago Ageitos A, Ares Alvarez J, del Rio Garma M, Garcia Cendon C, Goicoechea Castano A, Pena Nieto J. Prevalence of neurodevelopmental, behavioural and learning disorders in Pediatric Primary Care. Anales de Pediatria. 2018;89(3):153-61. |
| Carballo JJ, Rodriguez-Blanco L, Garcia-Nieto R, Baca-Garcia E. Screening for the ADHD Phenotype Using the Strengths and Difficulties Questionnaire in a Clinical Sample of Newly Referred Children and Adolescents. Journal of Attention Disorders. 2018;22(11):1032-9. |
| Carlsson LH, Norrelgen F, Kjellmer L, Westerlund J, Gillberg C, Fernell E. Coexisting disorders and problems in preschool children with autism spectrum disorders. Thescientificworldjournal. 2013;2013:213979. |
| Carpentier P-J, Knapen LJ, van Gogh MT, Buitelaar JK, De Jong CA. Addiction in developmental perspective: Influence of conduct disorder severity, subtype, and attention-deficit hyperactivity disorder on problem severity and comorbidity in adults with opioid dependence. Journal of Addictive Diseases. 2012;31(1):45-59. |
| Carvalho MC, Lima RFD, Souza GGBD, Pires TC, Pierini R, Rodrigues SDD, et al. Characterization of school-related problems and diagnoses in a Neuro-Learning Disorder Clinic. Estudos de Psicologia. 2016;33(1):161-71. |
| Celebi F, Koyuncu A, Ertekin E, Alyanak B, Tukel R. The Features of Comorbidity of Childhood ADHD in Patients With Obsessive Compulsive Disorder. Journal of Attention Disorders. 2020;24(7):973-80. |
| Cellucci T, Remsperger P, McGlade E. Psycho-educational evaluations for university students in one clinic. Psychological Reports. 2007;101(2):501-11. |
| Cengel-Kultur E, Cuhadaroglu-Cetin F, Gokler B. Demographic and clinical features of child abuse and neglect cases. Turkish Journal of Pediatrics. 2007;49(3):256-62. |
| Ceraudo G, Toni C, Vannucchi G, Rizzato S, Casalini F, Dell'Osso L, et al. Is substance use disorder with comorbid adult attention deficit hyperactivity disorder and bipolar disorder a distinct clinical phenotype? Heroin Addiction and Related Clinical Problems. 2012;14(3):71-6. |
| Chavira DA, Stein MB, Bailey K, Stein MT. Child anxiety in primary care: prevalent but untreated. Depression & Anxiety. 2004;20(4):155-64. |
| Chee KY, Sachdev P. The clinical features of Tourette's disorder: an Australian study using a structured interview schedule. Australian & New Zealand Journal of Psychiatry. 1994;28(2):313-8. |
| Cheung AS, Ooi O, Leemaqz S, Cundill P, Silberstein N, Bretherton I, et al. Sociodemographic and Clinical Characteristics of Transgender Adults in Australia. Transgender Health. 2018;3(1):229-38. |
| Chiasson J-P, Stavro K, Rizkallah E, Lapierre L, Dussault M, Legault L, Potvin S. Questioning the specificity of ASRS-v1.1 to accurately detect ADHD in substance abusing populations. Journal of Attention Disorders. 2012;16(8):661-3. |
| Chinawa JM, Odetunde OI, Obu HA, Chinawa AT, Bakare MO, Ujunwa FA. Attention deficit hyperactivity disorder: a neglected issue in the developing world. Behavioural Neurology. 2014;2014:694764. |
| Chung T. Does adolescents' readiness to change substance use behavior differ depending on profile of psychiatric comorbidity? Journal of Dual Diagnosis. 2006;2(1):73-88. |
| Clark DB, Pollock N, Bukstein OG, Mezzich AC, Bromberger JT, Donovan JE. Gender and comorbid psychopathology in adolescents with alcohol dependence. Journal of the American Academy of Child and Adolescent Psychiatry. 1997;36(9):1195-203. |
| Cleland C, Magura S, Foote J, Rosenblum A, Kosanke N. Factor structure of the Conners Adult ADHD Rating Scale (CAARS) for substance users. Addictive Behaviors. 2006;31(7):1277-82. |
| Clure C, Brady KT, Saladin ME, Johnson D, Waid R, Rittenbury M. Attention-deficit/hyperactivity disorder and substance use: symptom pattern and drug choice. American Journal of Drug & Alcohol Abuse. 1999;25(3):441-8. |
| Coetzee C, Truter I, Meyer A. Prevalence and characteristics of South African treatment-seeking patients with substance use disorder and co-occurring attention-deficit/hyperactivity disorder. Expert Review of Clinical Pharmacology. 2020;13(11):1271-80. |
| Coffey BJ, Miguel EC, Biederman J, Baer L, Rauch SL, O'Sullivan RL, et al. Tourette's disorder with and without obsessive-compulsive disorder in adults: Are they different? Journal of Nervous and Mental Disease. 1998;186(4):201-6. |
| Comin M, Braquehais MD, Valero S, Bel MJ, Navarro MC, Diaz A, et al. Inpatient physicians and nurses with dual diagnosis: An exploratory study. Journal of Dual Diagnosis. 2014;10(3):156-61. |
| Conejero I, Jaussent I, Lopez R, Guillaume S, Olie E, Hebbache C, et al. Association of symptoms of attention deficit-hyperactivity disorder and impulsive-aggression with severity of suicidal behavior in adult attempters. Scientific Reports. 2019;9(1):4593. |
| Connor DF, Ford JD, Albert DB, Doerfler LA. Conduct disorder subtype and comorbidity. Annals of Clinical Psychiatry. 2007;19(3):161-8. |
| Conway F, Oster M, Szymanski K. ADHD and complex trauma: A descriptive study of hospitalized children in an urban psychiatric hospital. Journal of Infant, Child & Adolescent Psychotherapy. 2011;10(1):60-72. |
| Coppola M, Sacchetto G, Mondola R. Craving for heroin: difference between methadone maintenance therapy patients with and without ADHD. Trends in Psychiatry & Psychotherapy. 2019;41(1):83-6. |
| Corbisiero S, Riecher-Rossler A, Buchli-Kammermann J, Stieglitz R-D. Symptom overlap and screening for symptoms of attention-deficit/hyperactivity disorder and psychosis risk in help-seeking psychiatric patients. Frontiers in Psychiatry Vol 8 2017, ArtID 206. 2017;8. |
| Crowley TJ, Macdonald MJ, Whitmore EA, Mikulich SK. Cannabis dependence, withdrawal, and reinforcing effects among adolescents with conduct symptoms and substance use disorders. Drug & Alcohol Dependence. 1998;50(1):27-37. |
| Crowley TJ, Mikulich SK, Ehlers KM, Whitmore EA, MacDonald MJ. Validity of structured clinical evaluations in adolescents with conduct and substance problems. Journal of the American Academy of Child & Adolescent Psychiatry. 2001;40(3):265-73. |
| Cunha PJ, Goncalves PD, Ometto M, Dos Santos B, Nicastri S, Busatto GF, de Andrade AG. Executive cognitive dysfunction and ADHD in cocaine dependence: searching for a common cognitive endophenotype for addictive disorders. Frontiers in psychiatry Frontiers Research Foundation. 2013;4:126. |
| Daigre C, Grau-Lopez L, Rodriguez-Cintas L, Egido A, Casas M, Roncero C. Administrative prevalence of insomnia and associated clinical features in patients with addiction during active substance use. Actas espanolas de psiquiatria. 2016;44(2):64-71. |
| Daigre C, Grau-Lopez L, Rodriguez-Cintas L, Ros-Cucurull E, Sorribes-Puertas M, Esculies O, et al. The role of dual diagnosis in health-related quality of life among treatment-seeking patients in Spain. Quality of Life Research. 2017;26(12):3201-9. |
| Daigre C, Ramos-Quiroga J, Valero S, Bosch R, Roncero C, Gonzalvo B, et al. Adult ADHD Self-Report Scale (ASRS-v1.1) symptom checklist in patients with substance use disorders. Actas Espanolas de Psiquiatria. 2009;37(6):299-305. |
| Daigre C, Roncero C, Grau-Lopez L, Martinez-Luna N, Prat G, Valero S, et al. Attention deficit hyperactivity disorder in cocaine-dependent adults: a psychiatric comorbidity analysis. American Journal on Addictions. 2013;22(5):466-73. |
| Daigre C, Roncero C, Rodriguez-Cintas L, Ortega L, Lligona A, Fuentes S, et al. Adult ADHD screening in alcohol-dependent patients using the Wender-Utah Rating Scale and the adult ADHD Self-Report Scale. Journal of Attention Disorders. 2015;19(4):328-34. |
| Dakwar E, Mahony A, Pavlicova M, Glass A, Brooks D, Mariani JJ, et al. The utility of attention-deficit/hyperactivity disorder screening instruments in individuals seeking treatment for substance use disorders. Journal of Clinical Psychiatry. 2012;73(11):e1372-8. |
| Danielyan A, Pathak S, Kowatch RA, Arszman SP, Johns ES. Clinical characteristics of bipolar disorder in very young children. Journal of Affective Disorders. 2007;97(1-3):51-9. |
| Davids E, von Bunau U, Specka M, Fischer B, Scherbaum N, Gastpar M. History of attention-deficit hyperactivity disorder symptoms and opioid dependence: a controlled study. Progress in Neuro-Psychopharmacology & Biological Psychiatry. 2005;29(2):291-6. |
| de Bruin EI, Ferdinand RF, Meester S, de Nijs PF, Verheij F. High rates of psychiatric co-morbidity in PDD-NOS. Journal of Autism & Developmental Disorders. 2007;37(5):877-86. |
| de los Cobos JP, Sinol N, Puerta C, Cantillano V, Zurita CL, Trujols J. Features and prevalence of patients with probable adult attention deficit hyperactivity disorder who request treatment for cocaine use disorders. Psychiatry Research. 2011;185(1-2):205-10. |
| de Veld L, van Hoof JJ, Wolberink IM, van der Lely N. The co-occurrence of mental disorders among Dutch adolescents admitted for acute alcohol intoxication. European Journal of Pediatrics. 2021;180(3):937-47. |
| de Vries HT, Nakamae T, Fukui K, Denys D, Narumoto J. Problematic internet use and psychiatric co-morbidity in a population of Japanese adult psychiatric patients. BMC Psychiatry. 2018;18(1):9. |
| Deas D, Friendly RW, Vo K, Johnson N, Upadhyaya H, Thomas SE. Dual diagnosis and drinking behaviors in an otupatient treatment seeking sample of adolescents with alcohol use disorders. Journal of Dual Diagnosis. 2005;2(1):47-57. |
| Debes NMMM, Lange T, Jessen TL, Hjalgrim H, Skov L. Performance on Wechsler intelligence scales in children with Tourette syndrome. European Journal of Paediatric Neurology. 2011;15(2):146-54. |
| Deepthi K, Sagar Kommu J, Smitha M, Reddy Y. Clinical profile and outcome in a large sample of children and adolescents with obsessive-compulsive disorder: A chart review from a tertiary care center in India. Indian Journal of Psychiatry. 2018;60(2):205-12. |
| Demeter CA, Youngstrom EA, Carlson GA, Frazier TW, Rowles BM, Lingler J, et al. Age differences in the phenomenology of pediatric bipolar disorder. Journal of Affective Disorders. 2013;147(1-3):295-303. |
| DeMilio L. Psychiatric syndromes in adolescent substance abusers. American Journal of Psychiatry. 1989;146(9):1212-4. |
| Di Nicola M, Sala L, Romo L, Catalano V, Even C, Dubertret C, et al. Adult attention-deficit/hyperactivity disorder in major depressed and bipolar subjects: role of personality traits and clinical implications. European Archives of Psychiatry & Clinical Neuroscience. 2014;264(5):391-400. |
| Diaz DR, Landsberger SA, Povlinski J, Sheward J, Sculley C. Psychiatric disorder prevalence among deaf and hard-of-hearing outpatients. Comprehensive Psychiatry. 2013;54(7):991-5. |
| Diaz R, Goti J, Garcia M, Gual A, Serrano L, Gonzalez L, et al. Patterns of substance use in adolescents attending a mental health department. European Child & Adolescent Psychiatry. 2011;20(6):279-89. |
| Doerfler LA, Connor DF, Toscano Jr PF. Aggression, ADHD symptoms, and dysphoria in children and adolescents diagnosed with bipolar disorder and ADHD. Journal of Affective Disorders. 2011;131(1-3):312-9. |
| Downey LVA, Zun LS. Identifying Undiagnosed Pediatric Mental Illness in the Emergency Department. Pediatric Emergency Care. 2018;34(2):e21-e3. |
| Downs J, Hotopf M, Ford T, Simonoff E, Jackson R, Shetty H, et al. Clinical predictors of antipsychotic use in children and adolescents with autism spectrum disorders: a historical open cohort study using electronic health records. European Child & Adolescent Psychiatry. 2016;25(6):649-58. |
| Durukan I, Karaman D, Kara K, Turker T, Tufan AE, Yalcin O, Karabekiroglu K. Diagnoses of patients referring to a child and adolescent psychiatry outpatient clinic. Dusunen Adam. 2011;24(2):113-20. |
| Eapen V, Al-Sabosy M, Saeed M, Sabri S. Child psychiatric disorders in a primary care Arab population. International Journal of Psychiatry in Medicine. 2004;34(1):51-60. |
| Eddy CM, Cavanna AE. Premonitory Urges in Adults With Complicated and Uncomplicated Tourette Syndrome. Behavior Modification. 2014;38(2):264-75. |
| El Ayoubi H, Barrault S, Gateau A, Cortese S, Frammery J, Mollat E, et al. Adult attention-deficit/hyperactivity disorder among alcohol use disorder inpatients is associated with food addiction and binge eating, but not BMI. Appetite. 2021;168:105665. |
| El Ayoubi H, Brunault P, Barrault S, Mauge D, Baudin G, Ballon N, El-Hage W. Posttraumatic Stress Disorder Is Highly Comorbid With Adult ADHD in Alcohol Use Disorder Inpatients. Journal of Attention Disorders. 2021;25(11):1594-602. |
| Endo T, Sugiyama T, Someya T. Attention-deficit/hyperactivity disorder and dissociative disorder among abused children. Psychiatry & Clinical Neurosciences. 2006;60(4):434-8. |
| Ertekin H, Karamustafalıoğlu O, Ertekin Y, Akpınar A, Bakım B, Tankaya O. The comorbidity of adult attention-deficit/hyperactivity disorder in panic disorder patients. Journal of Harran University Medical Faculty. 2013;10(3):108-13. |
| Ertekin H, Karamustafalioglu O, Ertekin YH, Bakim B, Akpinar A. Adult attention deficit and hyperactivity disorder comorbidity in obsessive compulsive disorder. Anatolian Journal of Clinical Investigation. 2015;9(4):167-73. |
| Etain B, Lajnef M, Loftus J, Henry C, Raust A, Gard S, et al. Association between childhood dimensions of attention deficit hyperactivity disorder and adulthood clinical severity of bipolar disorders. Australian and New Zealand Journal of Psychiatry. 2017;51(4):382-92. |
| Evren C, Umut G, Bozkurt M, Can Y, Evren B, Agachanli R. Partial mediator role of physical abuse on the relationship between attention-deficit/hyperactivity disorder symptoms and severity of dissociative experiences in a sample of inpatients with alcohol use disorder. Indian Journal of Psychiatry. 2017;59(3):306-12. |
| Evren C, Umut G, Bozkurt M, Teksin-Unal G, Agachanli R, Evren B. Psychometric properties of the Turkish version of the Adult ADHD Self-Report Scale (ASRS-v1.1) in a sample of Inpatients with alcohol use disorder. Dusunen Adam: Journal of Psychiatry and Neurological Sciences. 2016;29(2):109-19. |
| Fabricius V, Langa M, Wilson K. An exploratory investigation of co-occurring substancerelated and psychiatric disorders. Journal of Substance Use. 2008;13(2):99-114. |
| Fago DP. Evaluation and treatment of neurodevelopmental deficits in sexually aggressive children and adolescents. Professional Psychology: Research and Practice. 2003;34(3):248-57. |
| Falcone T, Mishra L, Carlton E, Lee C, Butler RS, Janigro D, et al. Suicidal behavior in adolescents with first-episode psychosis. Clinical Schizophrenia & Related Psychoses. 2010;4(1):34-40. |
| Faraone SV, Biederman J, Wozniak J, Mundy E, Mennin D, O'Donnell D. Is comorbidity with ADHD a marker for juvenile-onset mania? Journal of the American Academy of Child & Adolescent Psychiatry. 1997;36(8):1046-55. |
| Fatseas M, Hurmic H, Serre F, Debrabant R, Daulouede J-P, Denis C, Auriacombe M. Addiction severity pattern associated with adult and childhood Attention Deficit Hyperactivity Disorder (ADHD) in patients with addictions. Psychiatry Research. 2016;246:656-62. |
| Fava GA. Consultation psychiatry in an Italian Child Guidance Center: A report on 200 referrals. Child Psychiatry and Human Development. 1981;12(2)(5):90-5. |
| Feingold D, Nitzan U, Ratzoni G, Lev-Ran S. Clinical Correlates of Alcohol Abuse among Adolescent Psychiatric Inpatients in Israel. Israel Journal of Psychiatry & Related Sciences. 2014;51(4):258-60. |
| Ferre F, Cambra J, Ovejero M, Basurte-Villamor I. Influence of attention deficit hyperactivity disorder symptoms on quality of life and functionality in adults with eating disorders. Actas Espanolas de Psiquiatria. 2017;45(3):98-107. |
| Ferreira-Maia AP, Boronat AC, Boarati MA, Fu-I L, Wang Y-P. Evaluation of bipolar disorder in children and adolescents referred to a mood service: Diagnostic pathways and manic dimensions. Journal of Psychiatric Practice. 2016;22(6):429-41. |
| Ferrer M, Andion O, Matali J, Valero S, Navarro JA, Ramos-Quiroga JA, et al. Comorbid attention-deficit/hyperactivity disorder in borderline patients defines an impulsive subtype of borderline personality disorder. Journal of personality disorders. 2010;24(6):812-22. |
| Fischer M, Barkley RA, Smallish L, Fletcher K. Young adult follow-up of hyperactive children: Self-reported psychiatric disorders, comorbidity, and the role of childhood conduct problems and teen CD. Journal of Abnormal Child Psychology. 2002;30(5):463-75. |
| Fones CS, Pollack MH, Susswein L, Otto M. History of childhood attention deficit hyperactivity disorder (ADHD) features among adults with panic disorder. Journal of Affective Disorders. 2000;58(2):99-106. |
| Fossati A, Gratz KL, Borroni S, Maffei C, Somma A, Carlotta D. The relationship between childhood history of ADHD symptoms and DSM-IV borderline personality disorder features among personality disordered outpatients: the moderating role of gender and the mediating roles of emotion dysregulation and impulsivity. Comprehensive Psychiatry. 2015;56:121-7. |
| Fossati A, Novella L, Donati D, Donini M, Maffei C. History of childhood attention deficit/hyperactivity disorder symptoms and borderline personality disorder: a controlled study. Comprehensive Psychiatry. 2002;43(5):369-77. |
| Frazier J, Biederman J, Bellordre C, Garfield S, Geller D, Coffey B, Faraone S. Should the diagnosis of attention-deficit/hyperactivity disorder be considered in children with pervasive developmental disorder? Journal of Attention Disorders. 2001;4(4):203-11. |
| Freeman AJ, Youngstrom EA, Youngstrom JK, Findling RL. Disruptive Mood Dysregulation Disorder in a Community Mental Health Clinic: Prevalence, Comorbidity and Correlates. Journal of Child & Adolescent Psychopharmacology. 2016;26(2):123-30. |
| Frost RO, Steketee G, Tolin DF, Frost RO, Steketee G, Tolin DF. Comorbidity in hoarding disorder. Depression & Anxiety (1091-4269). 2011;28(10):876-84. |
| Gaber TJ, Bouyrakhen S, Herpertz-Dahlmann B, Hagenah U, Holtmann M, Freitag CM, et al. Migration background and juvenile mental health: a descriptive retrospective analysis of diagnostic rates of psychiatric disorders in young people. Glob Health Action. 2013;6:20187. |
| Gadow KD, Devincent CJ, Pomeroy J, Azizian A. Comparison of DSM-IV symptoms in elementary school-age children with PDD versus clinic and community samples. Autism. 2005;9(4):392-415. |
| Gadow KD, Sprafkin J, Nolan EE. DSM-IV Symptoms in community and clinic preschool children. Journal of the American Academy of Child & Adolescent Psychiatry. 2001;40(12):1383-92. |
| Ganesh S, Kandasamy A, Sahayaraj US, Benegal V. Adult Attention Deficit Hyperactivity Disorder in Patients with Substance Use Disorders: A Study from Southern India. Indian Journal of Psychological Medicine. 2017;39(1):59-62. |
| Gao K, Wang Z, Chen J, Kemp DE, Chan PK, Conroy CM, et al. Should an assessment of Axis I comorbidity be included in the initial diagnostic assessment of mood disorders? Role of QIDS-16-SR total score in predicting number of Axis I comorbidity. Journal of Affective Disorders. 2013;148(2-3):256-64. |
| Garakani A, Buono FD, Larkin K, Goldberg JF. Parsing the effects of comorbid adult ADHD and substance misuse on affective lability in bipolar disorder. Journal of Affective Disorders. 2020;266:338-40. |
| Garcia Marchena N, Araos P, Pavon FJ, Ponce G, Pedraz M, Serrano A, et al. Psychiatric comorbidity and plasma levels of 2-acyl-glycerols in outpatient treatment alcohol users. Analysis of gender differences. Adicciones. 2016;29(2):83-96. |
| Garland AF, Hough RL, McCabe KM, Yeh M, Wood PA, Aarons GA. Prevalence of psychiatric disorders in youths across five sectors of care. Journal of the American Academy of Child & Adolescent Psychiatry. 2001;40(4):409-18. |
| Garralda ME, Bailey D. Psychiatric disorders in general paediatric referrals. Archives of Disease in Childhood. 1989;64(12):1727-33. |
| Gelegen V, Tamam L. Prevalence and clinical correlates of intermittent explosive disorder in Turkish psychiatric outpatients. Comprehensive Psychiatry. 2018;83:64-70. |
| Ghanizadeh A. Association of nail biting and psychiatric disorders in children and their parents in a psychiatrically referred sample of children. Child and Adolescent Psychiatry and Mental Health. 2008;2 (no pagination). |
| Ghanizadeh A. Co-morbidity and factor analysis on attention deficit hyperactivity disorder and autism spectrum disorder DSM-IV-derived items. Journal of Research in Medical Sciences. 2012;17(4):368-72. |
| Ghaziuddin M, Weidmer-Mikhail E, Ghaziuddin N. Comorbidity of Asperger syndrome: a preliminary report. Journal of Intellectual Disability Research. 1998;42(Pt 4):279-83. |
| Gillberg I, Helles A, Billstedt E, Gillberg C. Boys with Asperger syndrome grow up: Psychiatric and neurodevelopmental disorders 20 years after initial diagnosis. Journal of Autism and Developmental Disorders. 2016;46(1):74-82. |
| Gjevik E, Eldevik S, Fjaeran-Granum T, Sponheim E. Kiddie-SADS reveals high rates of DSM-IV disorders in children and adolescents with autism spectrum disorders. Journal of Autism & Developmental Disorders. 2011;41(6):761-9. |
| Goldstein S, Schwebach AJ. The comorbidity of Pervasive Developmental Disorder and Attention Deficit Hyperactivity Disorder: results of a retrospective chart review. Journal of Autism & Developmental Disorders. 2004;34(3):329-39. |
| Gomez R, Vance A, Gomez RM. Validity of the ADHD Bifactor Model in General Community Samples of Adolescents and Adults, and a Clinic-Referred Sample of Children and Adolescents. Journal of Attention Disorders. 2018;22(14):1307-19. |
| Gomez RL, Janowsky D, Zetin M, Huey L, Clopton PL. Adult psychiatric diagnosis and symptoms compatible with the hyperactive child syndrome: a retrospective study. Journal of Clinical Psychiatry. 1981;42(10):389-94. |
| Goodman G, Gerstadt C, Pfeffer CR, Stroh M, Valdez A. ADHD and aggression as correlates of suicidal behavior in assaultive prepubertal psychiatric inpatients. Suicide & Life-Threatening Behavior. 2008;38(1):46-59. |
| Goossensen MA, van de Glind G, Carpentier PJ, Wijsen RMA, van Duin D, Kooij JJS. An intervention program for ADHD in patients with substance use disorders: Preliminary results of a field trial. Journal of Substance Abuse Treatment. 2006;30(3):253-9. |
| Gordon SM, Tulak F, Troncale J. Prevalence and characteristics of adolescents patients with co-occurring ADHD and substance dependence. Journal of Addictive Diseases. 2004;23(4):31-40. |
| Gordon-Lipkin E, Marvin AR, Law J, Lipkin PH. Anxiety and mood disorder in children with autism spectrum disorder and ADHD. Pediatrics. 2018;141(4):1-8. |
| Gorlin EI, Dalrymple K, Chelminski I, Zimmerman M. Diagnostic profiles of adult psychiatric outpatients with and without attention deficit hyperactivity disorder. Comprehensive Psychiatry. 2016;70:90-7. |
| Gorlin EI, Dalrymple K, Chelminski I, Zimmerman M. Reliability and validity of a semi-structured DSM-based diagnostic interview module for the assessment of Attention Deficit Hyperactivity Disorder in adult psychiatric outpatients. Psychiatry Research. 2016;242:46-53. |
| Gorman DA, Thompson N, Plessen KJ, Robertson MM, Leckman JF, Peterson BS. Psychosocial outcome and psychiatric comorbidity in older adolescents with Tourette syndrome: controlled study. British Journal of Psychiatry. 2010;197(1):36-44. |
| Gould KL, Porter M, Lyneham HJ, Hudson JL. Cognitive-Behavioral Therapy for Children With Anxiety and Comorbid Attention-Deficit/Hyperactivity Disorder. Journal of the American Academy of Child and Adolescent Psychiatry. 2018;57(7):481-90.e2. |
| Grall-Bronnec M, Wainstein L, Augy J, Bouju G, Feuillet F, Venisse J-L, Sebille-Rivain V. Attention deficit hyperactivity disorder among pathological and at-risk gamblers seeking treatment: A hidden disorder. European Addiction Research. 2011;17(5):231-40. |
| Groth C, Mol Debes N, Rask CU, Lange T, Skov L. Course of Tourette Syndrome and Comorbidities in a Large Prospective Clinical Study. Journal of the American Academy of Child and Adolescent Psychiatry. 2017;56(4):304-12. |
| Gupta JK, Rohiwal S, Marwah P, Saini A, Maheshwari A, Gupta S, Saini P. The pattern of psychiatric morbidity in an out-patient child psychiatry clinic: A study from a tertiary care centre in Jaipur, North India. Journal of Cardiovascular Disease Research. 2021;12(4):622-7. |
| Gupta PK, Sivakumar T, Agarwal V, Sitholey P. A clinical study of phenomenology and comorbidity of paediatric bipolar disorder. Journal of Indian Association for Child and Adolescent Mental Health. 2012;8(1):12-9. |
| Gupta S, Bhatia G, Sarkar S, Chatterjee B, Balhara YPS, Dhawan A. Adult attention-deficit hyperactivity disorders and its correlates in patients with opioid dependence: An exploratory study. Indian Journal of Psychiatry. 2020;62(5):501-8. |
| Gurkan K, Akcakin M, Kilic BG, Bilgic A. Psychiatric comorbidity and drug treatments in high functioning children and adolescents with Pervasive Developmental Disorders. Neurology Psychiatry and Brain Research. 2008;15(3):143-50. |
| Gyllenberg D, Gissler M, Malm H, Artama M, Hinkka-Yli-Salomaki S, Brown AS, Sourander A. Specialized service use for psychiatric and neurodevelopmental disorders by age 14 in Finland. Psychiatric Services. 2014;65(3):367-73. |
| Hallerback MU, Lugnegard T, Gillberg C. ADHD and nicotine use in schizophrenia or Asperger syndrome: a controlled study. Journal of Attention Disorders. 2014;18(5):425-33. |
| Halloran EC, Ross GJ, Carey MP. The relationship of adolescent personality and family environment to psychiatric diagnosis. Child Psychiatry and Human Development. 2002;32(3):201-16. |
| Hanley C, Saleem F, Graffeo I, McCarthy G, Gavin B, McNicholas F, Adamis D. Association of Wender Utah Rating Scale (WURS)-61 items with clinical psychiatric diagnosis in adulthood. Irish Journal of Medical Science. 2021;04:04. |
| Hansen BH, Oerbeck B, Skirbekk B, Kristensen H. Non-obsessive-compulsive anxiety disorders in child and adolescent mental health services -Are they underdiagnosed, and how accurate is referral information? Nordic Journal of Psychiatry. 2016;70(2):133-9. |
| Hansen BH, Oerbeck B, Skirbekk B, Petrovski BE, Kristensen H. Neurodevelopmental disorders: prevalence and comorbidity in children referred to mental health services. Nordic Journal of Psychiatry. 2018;72(4):285-91. |
| Hanson E, Cerban B, Slater C, Caccamo L, Bacic J, Chan E. Brief Report: Prevalence of Attention Deficit/Hyperactivity Disorder Among Individuals with an Autism Spectrum Disorder. Journal of Autism & Developmental Disorders. 2013;43(6):1459-64. |
| Harada Y, Hayashida A, Hikita S, Imai J, Sasayama D, Masutani S, et al. Impact of behavioral/developmental disorders comorbid with conduct disorder. Psychiatry & Clinical Neurosciences. 2009;63(6):762-8. |
| Hardan A, Sahl R. Psychopathology in children and adolescents with developmental disorders. Research in Developmental Disabilities. 1997;18(5):369-82. |
| Harding G, Hu N, Larter N, Montgomery A, Stephensen J, Callaghan L, et al. Health status and health service use of urban Aboriginal children attending an Aboriginal community child health service in Sydney. Journal of Paediatrics and Child Health. 2021;57(7):1072-7. |
| Harmanci H, ÇElİKel FÇ, EtİKan İ. Comorbidity of Adult Attention Deficit and Hyperactivity Disorder in Bipolar and Unipolar Patients. Archives of Neuropsychiatry / Noropsikiatri Arsivi. 2016;53(3):257-62. |
| Haw C, Hawton K, Houston K, Townsend E. Psychiatric and personality disorders in deliberate self-harm patients. British Journal of Psychiatry. 2001;178(JAN.):48-54. |
| Hayashida K, Anderson B, Paparella T, Freeman SFN, Forness SR. Comorbid Psychiatric Diagnoses in Preschoolers with Autism Spectrum Disorders. Behavioral Disorders. 2017;35(3):243-54. |
| Heinrichs N, Kamp-Becker I, Bussing R, Schimek M, Becker A, Briegel W. Disruptive behaviors across different disorders: Evaluation of a clinical sample using the eyberg child behavior inventory+. Zeitschrift fur Kinder- und Jugendpsychiatrie und Psychotherapie. 2019;47(1):35-47. |
| Henin A, Biederman J, Mick E, Hirshfeld-Becker DR, Sachs GS, Wu Y, et al. Childhood antecedent disorders to bipolar disorder in adults: a controlled study. Journal of Affective Disorders. 2007;99(1-3):51-7. |
| Hesse M. Course of self-reported symptoms of attention deficit and hyperactivity in substance abusers during early treatment. Addictive Behaviors. 2010;35(5):504-6. |
| Hirschtritt ME, Pauls DL, Dion Y, Grados MA, Illmann C, King RA, et al. Lifetime prevalence, age of risk, and genetic relationships of comorbid psychiatric disorders in Tourette Syndrome. JAMA Psychiatry. 2015;72(4):325-33. |
| Hofvander B, Delorme R, Chaste P, Nyden A, Wentz E, Stahlberg O, et al. Psychiatric and psychosocial problems in adults with normal-intelligence autism spectrum disorders. BMC Psychiatry. 2009;9:35. |
| Holtmann M, Duketis E, Poustka L, Zepf FD, Poustka F, Bolte S. Bipolar disorder in children and adolescents in Germany: national trends in the rates of inpatients, 2000-2007. Bipolar Disorders. 2010;12(2):155-63. |
| Hong JS, Singh V, Kalb L. Attention Deficit Hyperactivity Disorder Symptoms in Young Children with Autism Spectrum Disorder. Autism research : Official Journal of the International Society for Autism Research. 2021;14(1):182-92. |
| Hong M, Kooij J, Kim B, Joung Y-S, Yoo HK, Kim E-J, et al. Validity of the Korean version of DIVA-5: A semi-structured diagnostic interview for adult ADHD. Neuropsychiatric Disease and Treatment Vol 16 2020, ArtID 2371-2376. 2020;16. |
| Horton AM, Fiscella R, Schwartz N, Anilane J, et al. Diagnosis of attention deficit disorder in adult alcoholics: The case of DSM III-R. Psychotherapy in Private Practice. 1990;8(3):123-7. |
| Huntley Z, Maltezos S, Williams C, Morinan A, Hammon A, Ball D, et al. Rates of undiagnosed attention deficit hyperactivity disorder in London drug and alcohol detoxification units. BMC Psychiatry Vol 12 2012, ArtID 223. 2012;12. |
| Icick R, Moggi F, Slobodin O, Dom G, Mathys F, Van Den Brink W, et al. Attention Deficit/Hyperactivity Disorder and Global Severity Profiles in Treatment-Seeking Patients with Substance Use Disorders. European Addiction Research. 2020;26(4-5):201-10. |
| Irastorza Eguskiza LJ, Bellon JM, Mora M. Comorbidity of personality disorders and attention-deficit hyperactivity disorder in adults. Revista De Psiquiatria Y Salud Mental. 2018;11(3):151-5. |
| Isaac G. Is bipolar disorder the most common diagnostic entity in hospitalized adolescents and children? Adolescence. 1995;30(118):273-6. |
| Ishizuka K, Ishiguro T, Nomura N, Inada T. Depressive mood changes are associated with self-perceptions of ADHD characteristics in adults. Psychiatry Research. 2021;300:113893. |
| Ivanenko A, Crabtree VM, O'Brien LM, Gozal D. Sleep complaints and psychiatric symptoms in children evaluated at a pediatric mental health clinic. Journal of Clinical Sleep Medicine. 2006;2(1):42-8. |
| Jafferany M, Osuagwu FC, Khalid Z, Oberbarnscheidt T, Roy N. Prevalence and clinical characteristics of body dysmorphic disorder in adolescent inpatient psychiatric patients-a pilot study. Nordic Journal of Psychiatry. 2019;73(4-5):244-7. |
| Jager I, de Koning P, Bost T, Denys D, Vulink N. Misophonia: Phenomenology, comorbidity and demographics in a large sample. PLoS ONE [Electronic Resource]. 2020;15(4):e0231390. |
| Jaideep T, Reddy Y, Srinath S. Comorbidity of attention deficit hyperactivity disorder in juvenile bipolar disorder. Bipolar Disorders. 2006;8(2):182-7. |
| Jainchill N, De Leon G, Yagelka J. Ethnic differences in psychiatric disorders among adolescent abusers in treatment. Journal of Psychopathology and Behavioral Assessment. 1997;19(2):133-48. |
| Jaisoorya T, Reddy Y, Srinath S. Is juvenile obsessive-compulsive disorder a developmental subtype of the disorder? Findings from an Indian study. European Child & Adolescent Psychiatry. 2003;12(6):290-7. |
| James PD, Smyth BP, Apantaku-Olajide T. Substance use and psychiatric disorders in Irish adolescents: A cross-sectional study of patients attending substance abuse treatment service. Mental Health and Substance Use: Dual Diagnosis. 2013;6(2):124-32. |
| Jankovic J, Gelineau-Kattner R, Davidson A. Tourette's syndrome in adults. Movement Disorders. 2010;25(13):2171-5. |
| Janoczkin A, Kiers S, Edara N, He P, Li Y. Impact of COVID-19 pandemic on emergency psychiatry-Millcreek community hospital, Erie, PA. Comprehensive Psychiatry. 2021;110 (no pagination). |
| Jaworowski S, Mergui J, Golmard J-L, Fine N, Alfassi T, Lavy DG, et al. Prevalence of Attention Deficit Hyperactivity Disorder Among 255 Patients in Three Israeli Methadone Clinics62. Israel Journal of Psychiatry & Related Sciences. 2020;57(1):55-62. |
| Jayaprakash R, Rajamohanan K, Anil P. Determinants of symptom profile and severity of conduct disorder in a tertiary level pediatric care set up: A pilot study. Indian Journal of Psychiatry. 2014;56(4):330-6. |
| Jerrell JM, McIntyre RS, Deroche CB. Diagnostic clusters associated with an early onset schizophrenia diagnosis among children and adolescents. Human Psychopharmacology. 2017;32(2):03. |
| Jerrell JM, Prewette ED, II. Outcomes for youths with early- and very-early-onset bipolar I disorder. The Journal of Behavioral Health Services & Research. 2008;35(1):52-9. |
| Jerrell JM, Shugart MA. Community-based care for youths with early and very-early onset bipolar I disorder. Bipolar Disorders. 2004;6(4):299-304. |
| Jiang L, Li Y, Zhang X, Jiang W, Yang C, Hao N, et al. Twelve-year retrospective analysis of outpatients with Attention-Deficit/Hyperactivity Disorder in Shanghai. Shanghai Jingshen Yixue. 2013;25(4):236-42. |
| Johnston K, Dittner A, Bramham J, Murphy C, Knight A, Russell A. Attention deficit hyperactivity disorder symptoms in adults with autism spectrum disorders. Autism research : Official Journal of the International Society for Autism Research. 2013;6(4):225-36. |
| Joo E-J, Lee KY, Choi K-S, Kim SH, Song JY, Bang YW, et al. Childhood attention deficit hyperactivity disorder features in adult mood disorders. Comprehensive Psychiatry. 2012;53(3):217-23. |
| Kafka MP, Hennen J. A DSM-IV Axis I comorbidity study of males (n = 120) with paraphilias and paraphilia-related disorders. Sexual Abuse: Journal of Research & Treatment. 2002;14(4):349-66. |
| Kafka MP, Prentky RA. Attention-deficit/hyperactivity disorder in males with paraphilias and paraphilia-related disorders: a comorbidity study. Journal of Clinical Psychiatry. 1998;59(7):388-96; quiz 97. |
| Kamal RM, Dijkstra BAG, de Weert-van Oene GH, van Duren JAM, de Jong CAJ. Psychiatric comorbidity, psychological distress, and quality of life in gamma-hydroxybutyrate-dependent patients. Journal of Addictive Diseases. 2017;36(1):72-9. |
| Kambanis PE, Kuhnle MC, Wons OB, Jo JH, Keshishian AC, Hauser K, et al. Prevalence and correlates of psychiatric comorbidities in children and adolescents with full and subthreshold avoidant/restrictive food intake disorder. International Journal of Eating Disorders. 2020;53(2):256-65. |
| Kantzer A-K, Fernell E, Westerlund J, Hagberg B, Gillberg C, Miniscalco C. Young children who screen positive for autism: Stability, change and "comorbidity" over two years. Research in Developmental Disabilities. 2018;72:297-307. |
| Karaahmet E, Konuk N, Dalkilic A, Saracli O, Atasoy N, Kurcer MA, Atik L. The comorbidity of adult attention-deficit/hyperactivity disorder in bipolar disorder patients. Comprehensive Psychiatry. 2013;54(5):549-55. |
| Karlsdotter K, Bushe C, Hakkaart L, Sobanski E, Kan CC, Lebrec J, et al. Burden of illness and health care resource utilization in adult psychiatric outpatients with attention-deficit/hyperactivity disorder in Europe. Current Medical Research and Opinion. 2016;32(9):1547-56. |
| Karlsson AT, Vederhus JK, Clausen T, Weimand B, Solli KK, Tanum L. Levels of Impulsivity, Hyperactivity, and Inattention and the Association with Mental Health and Substance Use Severity in Opioid-Dependent Patients Seeking Treatment with Extended-Release Naltrexone. Journal of Clinical Medicine. 2021;10(19):30. |
| Kast KA, Rao V, Wilens TE. Pharmacotherapy for Attention-Deficit/Hyperactivity Disorder and Retention in Outpatient Substance Use Disorder Treatment: A Retrospective Cohort Study. Journal of Clinical Psychiatry. 2021;82(2):23. |
| Kawabe K, Horiuchi F, Miyama T, Jogamoto T, Aibara K, Ishii E, Ueno S-i. Internet addiction and attention-deficit / hyperactivity disorder symptoms in adolescents with autism spectrum disorder. Research in Developmental Disabilities. 2019;89:22-8. |
| Kaye S, Ramos-Quiroga JA, van de Glind G, Levin FR, Faraone SV, Allsop S, et al. Persistence and subtype stability of ADHD among substance use disorder treatment seekers. Journal of Attention Disorders. 2019;23(12):1438-53. |
| Keller KM, Fox RA. Toddlers with developmental delays and challenging behaviors. Early Child Development and Care. 2009;179(1):87-92. |
| Kelly TM, Cornelius JR, Clark DB, Kelly TM, Cornelius JR, Clark DB. Psychiatric disorders and attempted suicide among adolescents with substance use disorders. Drug & Alcohol Dependence. 2004;73(1):87-97. |
| Kennemer K, Goldstein S. Incidence of ADHD in adults with severe mental health problems. Applied Neuropsychology. 2005;12(2):77-82. |
| Kenneson A, Funderburk JS, Maisto SA. Risk factors for secondary substance use disorders in people with childhood and adolescent-onset bipolar disorder: opportunities for prevention. Comprehensive Psychiatry. 2013;54(5):439-46. |
| Kessing LV, Vradi E, Andersen PK. Diagnostic stability in pediatric bipolar disorder. Journal of Affective Disorders. 2015;172:417-21. |
| Kewitz S, Vonderlin E, Wartberg L, Lindenberg K. Estimated Prevalence of Unreported IGD Cases in Routine Outpatient Children and Adolescent Psychotherapy. International Journal of Environmental Research & Public Health [Electronic Resource]. 2021;18(13):24. |
| Khairkar P, Pathak C, Lakhkar B, Sarode R, Vagha J, Jagzape T, et al. A 5-year hospital prevalence of child and adolescent psychiatric disorders from central India. Indian Journal of Pediatrics. 2013;80(10):826-31. |
| Kim J-W, Park C-S, Hwang J-W, Shin M-S, Hong K-E, Cho S-C, Kim B-N. Clinical and genetic characteristics of Korean male alcoholics with and without attention deficit hyperactivity disorder. Alcohol and Alcoholism. 2006;41(4):407-11. |
| King VL, Brooner RK, Kidorf MS, Stoller KB, Mirsky AF. Attention deficit hyperactivity disorder and treatment outcome in opioid abusers entering treatment. Journal of Nervous & Mental Disease. 1999;187(8):487-95. |
| Kommu JVS, Gayathri K, Srinath S, Girimaji SC, Seshadri SP, Gopalakrishna G, Subbakrishna DK. Profile of two hundred children with Autism Spectrum Disorder from a tertiary child and adolescent psychiatry centre. Asian Journal of Psychiatry. 2017;28:51-6. |
| Konrad K, Kohls G, Baumann S, Bernhard A, Martinelli A, Ackermann K, et al. Sex differences in psychiatric comorbidity and clinical presentation in youths with conduct disorder. Journal of Child Psychology & Psychiatry & Allied Disciplines. 2021;19:19. |
| Kopp S, Gillberg C. Swedish child and adolescent psychiatric out-patients: A five-year cohort. European Child and Adolescent Psychiatry. 2003;12(1):30-5. |
| Korsgaard HO, Torgersen S, Wentzel-Larsen T, Ulberg R. Substance abuse and personality disorder comorbidity in adolescent outpatients: Are girls more severely ill than boys? Child and Adolescent Psychiatry and Mental Health Vol 10 2016, ArtID 8. 2016;10. |
| Kousha M, Mehdizadeh Tehrani S. Normative life events and PTSD in children: how easy stress can affect children's brain. Acta Medica Iranica. 2013;51(1):47-51. |
| Koyuncu A, Celebi F, Ertekin E, Kok BE, Tukel R. Clinical Effects of ADHD Subtypes in Patients With Social Anxiety Disorder. Journal of Attention Disorders. 2019;23(12):1464-9. |
| Koyuncu A, ÇElebi F, Ertekin E, MemiŞ ÇÖ, TÜKel R, Memiş ÇÖ, Tükel R. The Presence of Childhood Attention Deficit/Hyperactivity Disorder May Be Associated With Interpersonal Sensitivity in Patients With Social Anxiety Disorder. Journal of Psychiatric Practice®. 2017;23(4):254-9. |
| Koyuncu A, Ertekin E, Yüksel Ç, Aslantas Ertekin B, Çelebi F, Binbay Z, et al. Predominantly Inattentive Type of ADHD is Associated With Social Anxiety Disorder. Journal of Attention Disorders. 2015;19(10):856-64. |
| Kragh K, Husby M, Melin K, Weidle B, Torp NC, Hojgaard DR, et al. Convergent and divergent validity of the Schedule for Affective Disorders and Schizophrenia for School-Age Children-Present and Lifetime Version diagnoses in a sample of children and adolescents with obsessive-compulsive disorder. Nordic Journal of Psychiatry. 2019;73(2):111-7. |
| Kronstrom K, Ellila H, Kuosmanen L, Kaljonen A, Sourander A. Changes in the clinical features of child and adolescent psychiatric inpatients: a nationwide time-trend study from Finland. Nordic Journal of Psychiatry. 2016;70(6):436-41. |
| Kulacaoglu F, Solmaz M, Belli H, Ardic FC, Akin E, Kose S. The relationship between impulsivity and attention-deficit/hyperactivity symptoms in female patients with borderline personality disorder. Psychiatry and Clinical Psychopharmacology. 2017;27(3):255-61. |
| Kumar G, Faden J, Steer RA. Screening for attention-deficit/hyperactivity disorder in adult inpatients with psychiatric disorders. Psychological Reports. 2011;108(3):815-24. |
| Kumar SV, Chate SS, Patil NM, Tekalaki BV, Patil S. Prevalence of undiagnosed and untreated attention deficit hyperactivity disorder in men with alcohol dependence: A case-control study. Archives of Psychiatry and Psychotherapy. 2018;20(1):26-32. |
| Kuperman S, Schlosser SS, Kramer JR, Bucholz K, Hesselbrock V, Reich T, Reich W. Developmental sequence from disruptive behavior diagnosis to adolescent alcohol dependence. American Journal of Psychiatry. 2001;158(12):2022-6. |
| Kusaka H, Miyawaki D, Nakai Y, Okamoto H, Futoo E, Goto A, et al. Psychiatric comorbidity in children with high-functioning pervasive developmental disorder. Osaka City Medical Journal. 2014;60(1):1-10. |
| Kutcher SP, Marton P, Korenblum M. Relationship between psychiatric illness and conduct disorder in adolescents. Canadian Journal of Psychiatry - Revue Canadienne de Psychiatrie. 1989;34(6):526-9. |
| Lamanna AL, Craig F, Matera E, Simone M, Buttiglione M, Margari L. Risk factors for the existence of attention deficit hyperactivity disorder symptoms in children with autism spectrum disorders. Neuropsychiatric Disease & Treatment. 2017;13:1559-67. |
| Lazaro L, Castro-Fornieles J, de la Fuente JE, Baeza I, Morer A, Pamias M. Differences between prepubertal- versus adolescent- onset bipolar disorder in a Spanish clinical sample. European Child & Adolescent Psychiatry. 2007;16(8):510-6. |
| Leader G, Moore R, Chen JL, Caher A, Arndt S, Maher L, et al. Attention deficit hyperactivity disorder (ADHD) symptoms, comorbid psychopathology, behaviour problems and gastrointestinal symptoms in children and adolescents with autism spectrum disorder. Irish Journal of Psychological Medicine. 2021:1-11. |
| Lecavalier L, McCracken CE, Aman MG, McDougle CJ, McCracken JT, Tierney E, et al. An exploration of concomitant psychiatric disorders in children with autism spectrum disorder. Comprehensive Psychiatry. 2019;88:57-64. |
| Lee AR, Bahn GH. Trends of Mental Disorders and Treatment Continuity Predictors of New Patients in the Paediatric Psychiatry Clinic of a University Hospital. International Journal of Environmental Research & Public Health [Electronic Resource]. 2021;18(18):12. |
| Lee DO, Ousley OY. Attention-deficit hyperactivity disorder symptoms in a clinic sample of children and adolescents with pervasive developmental disorders. Journal of Child & Adolescent Psychopharmacology. 2006;16(6):737-46. |
| Lencz T, Smith CW, Auther A, Correll CU, Cornblatt B. Nonspecific and attenuated negative symptoms in patients at clinical high-risk for schizophrenia. Schizophrenia Research. 2004;68(1):37-48. |
| Leopold K, Ratzer S, Correll CU, Rottmann-Wolf M, Pfeiffer S, Ritter P, et al. Characteristics, symptomatology and naturalistic treatment in individuals at-risk for bipolar disorders: baseline results in the first 180 help-seeking individuals assessed at the Dresden high-risk project. Journal of Affective Disorders. 2014;152-154:427-33. |
| Leung V, Chan L. A cross-sectional cohort study of prevalence, co-morbidities, and correlates of attention-deficit hyperactivity disorder among adult patients admitted to the Li Ka Shing psychiatric outpatient clinic, Hong Kong. East Asian Archives of Psychiatry. 2017;27(2):63-70. |
| Levin FR, Evans SM, Kleber HD. Prevalence of adult attention-deficit hyperactivity disorder among cocaine abusers seeking treatment. Drug & Alcohol Dependence. 1998;52(1):15-25. |
| Lev-Ran S, Aviram A, Braw Y, Nitzan U, Ratzoni G, Fennig S. Clinical correlates of cannabis use among adolescent psychiatric inpatients. European Psychiatry: the Journal of the Association of European Psychiatrists. 2012;27(6):470-5. |
| Lewczyk CM, Garland AF, Hurlburt MS, Gearity J, Hough RL. Comparing DISC-IV and clinician diagnoses among youths receiving public mental health services. Journal of the American Academy of Child & Adolescent Psychiatry. 2003;42(3):349-56. |
| Lewin AB, Chang S, McCracken J, McQueen M, Piacentini J. Comparison of clinical features among youth with tic disorders, obsessive-compulsive disorder (OCD), and both conditions. Psychiatry Research. 2010;178(2):317-22. |
| Lohit SR, Babu GN, Sharma S, Rao S, Sachin BS, Matkar AV. Prevalence of Adult ADHD Co-morbidity in Alcohol Use Disorders in a General Hospital Setup. Indian Journal of Psychological Medicine. 2019;41(6):523-8. |
| Lowe TL, Capriotti MR, McBurnett K. Long-Term Follow-up of Patients with Tourette's Syndrome. Movement Disorders Clinical Practice. 2019;6(1):40-5. |
| Luderer M, Kaplan-Wickel N, Richter A, Reinhard I, Kiefer F, Weber T. Screening for adult attention-deficit/hyperactivity disorder in alcohol dependent patients: Underreporting of ADHD symptoms in self-report scales. Drug & Alcohol Dependence. 2019;195:52-8. |
| Lugoboni F, Levin FR, Pieri MC, Manfredini M, Zamboni L, Somaini L, Gerra. Co-occurring Attention Deficit Hyperactivity Disorder symptoms in adults affected by heroin dependence: Patients characteristics and treatment needs. Psychiatry Research. 2017;250:210-6. |
| Lumley VA, McNeil CB, Herschell AD, Bahl AB. An examination of gender differences among young children with disruptive behavior disorders. Child Study Journal. 2002;32(2):89-100. |
| Luoni C, Agosti M, Crugnola S, Rossi G, Termine C. Psychopathology, Dissociation and Somatic Symptoms in Adolescents Who Were Exposed to Traumatic Experiences. Frontiers in Psychology. 2018;9:2390. |
| Magruder KM, Sonne SC, Brady KT, Quello S, Martin RH. Screening for Co-occurring Mental Disorders in Drug Treatment Populations. Journal of Drug Issues. 2005;35(3):593-606. |
| Malhotra S, Biswas P, Sharan P, Grover S. Characteristics of patients visiting the child & adolescent psychiatric clinic: A 26-year study from North India. Journal of Indian Association for Child and Adolescent Mental Health. 2007;3(3):53-60. |
| Mancini C, Van Ameringen M, Oakman JM, Figueiredo D. Childhood attention deficit/hyperactivity disorder in adults with anxiety disorders. Psychological Medicine. 1999;29(3):515-25. |
| Mandell DS, Ittenbach RF, Levy SE, Pinto-Martin JA. Disparities in diagnoses received prior to a diagnosis of autism spectrum disorder. Journal of Autism & Developmental Disorders. 2007;37(9):1795-802. |
| Manohar H, Kuppili PP, Kandasamy P, Chandrasekaran V, Rajkumar RP. Implications of comorbid ADHD in ASD interventions and outcome: Results from a naturalistic follow up study from south India. Asian Journal of Psychiatry. 2018;33:68-73. |
| Manor I, Gutnik I, Ben-Dor D, Apter A, Sever J, Tyano S, et al. Possible association between attention deficit hyperactivity disorder and attempted suicide in adolescents-A pilot study. European Psychiatry. 2010;25(3):146-50. |
| Marin A, Scott D, Groll DL. Bipolar disorder comorbid with attention-deficit/hyperactivity disorder in adult inpatients with acute relapse. The Primary Care Companion to CNS Disorders. 2013;15(4). |
| Marks S, Shaikh U, Hilty DM, Cole S. Weight status of children and adolescents in a telepsychiatry clinic. Telemedicine Journal & E-Health. 2009;15(10):970-4. |
| Martins J, Roberts N, Nesdole R, Reddy PS, Groll D. Attention deficit hyperactivity disorder presentations to child and adolescent mental health urgent consult clinic. Journal of the Canadian Academy of Child and Adolescent Psychiatry. 2019;28(2):66-71. |
| Marwitz L, Pringsheim T. Clinical Utility of Screening for Anxiety and Depression in Children with Tourette Syndrome. Journal of the Canadian Academy of Child & Adolescent Psychiatry = Journal de l’Academie canadienne de psychiatrie de l’enfant et de l’adolescent. 2018;27(1):15-21. |
| Masi G, Berloffa S, Muratori P, Mucci M, Viglione V, Villafranca A, et al. A Naturalistic Study of Youth Referred to a Tertiary Care Facility for Acute Hypomanic or Manic Episode. Brain Sciences. 2020;10(10):29. |
| Masi G, Millepiedi S, Mucci M, Bertini N, Pfanner C, Arcangeli F. Comorbidity of obsessive-compulsive disorder and attention-deficit/hyperactivity disorder in referred children and adolescents. Comprehensive Psychiatry. 2006;47(1):42-7. |
| Masi G, Milone A, Manfredi A, Pari C, Paziente A, Millepiedi S. Comorbidity of conduct disorder and bipolar disorder in clinically referred children and adolescents. Journal of Child & Adolescent Psychopharmacology. 2008;18(3):271-9. |
| Masi G, Milone A, Manfredi A, Pari C, Paziente A, Millepiedi S. Conduct disorder in referred children and adolescents: clinical and therapeutic issues. Comprehensive Psychiatry. 2008;49(2):146-53. |
| Masi G, Perugi G, Millepiedi S, Mucci M, Pari C, Pfanner C, et al. Clinical implications of DSM-IV subtyping of bipolar disorders in referred children and adolescents. Journal of the American Academy of Child & Adolescent Psychiatry. 2007;46(10):1299-306. |
| Masi G, Perugi G, Millepiedi S, Toni C, Mucci M, Bertini N, et al. Clinical and research implications of panic-bipolar comorbidity in children and adolescents. Psychiatry Research. 2007;153(1):47-54. |
| Masi G, Perugi G, Toni C, Millepiedi S, Mucci M, Bertini N, Akiskal HS. Obsessive-compulsive bipolar comorbidity: focus on children and adolescents. Journal of Affective Disorders. 2004;78(3):175-83. |
| Masi G, Perugi G, Toni C, Millepiedi S, Mucci M, Bertini N, Akiskal HS. Predictors of treatment nonresponse in bipolar children and adolescents with manic or mixed episodes. Journal of Child & Adolescent Psychopharmacology. 2004;14(3):395-404. |
| Masi G, Perugi G, Toni C, Millepiedi S, Mucci M, Bertini N, Pfanner C. Attention-deficit hyperactivity disorder - Bipolar comorbidity in children and adolescents. Bipolar Disorders. 2006;8(4):373-81. |
| Matthews M, Bell E, Mirfin-Veitch B. Comparing psychopathology rates across autism spectrum disorders and intellectual disabilities. Advances in Mental Health and Intellectual Disabilities. 2018;12(5-6):163-72. |
| Mayne SL, Ross ME, Lihai S, McCarn B, Steffes J, Weiwei L, et al. Variations in Mental Health Diagnosis and Prescribing Across Pediatric Primary Care Practices. Pediatrics. 2016;137(5):1-10. |
| McAweeney M, Rogers NL, Huddleston C, Moore D, Gentile JP. Symptom prevalence of ADHD in a community residential substance abuse treatment program. Journal of Attention Disorders. 2010;13(6):601-8. |
| McIntyre RS, Kennedy SH, Soczynska JK, Nguyen HTT, Bilkey TS, Woldeyohannes HO, et al. Attention-deficit/hyperactivity disorder in adults with bipolar disorder or major depressive disorder: Results from the international mood disorders collaborative project. Primary Care Companion to the Journal of Clinical Psychiatry. 2010;12(3). |
| McPhate L, Williams K, Vance A, Winther J, Pang K, May T. Gender Variance in Children and Adolescents with Neurodevelopmental and Psychiatric Conditions from Australia. Archives of Sexual Behavior. 2021;50(3):863-71. |
| Mihan R, Shahrivar Z, Mahmoudi-Gharaei J, Shakiba A, Hosseini M. Attention-Deficit Hyperactivity Disorder in Adults Using Methamphetamine: Does It Affect Comorbidity, Quality of Life, and Global Functioning? Iranian Journal of Psychiatry. 2018;13(2):111-8. |
| Milin R, Loh E, Chow J, Wilson A. Assessment of symptoms of attention-deficit hyperactivity disorder in adults with substance use disorders. Psychiatric Services. 1997;48(11):1378-80, 95. |
| Miovsky M, Lukavska K, Rubasova E, Stastna L, Sefranek M, Gabrhelik R. Attention Deficit Hyperactivity Disorder among Clients Diagnosed with a Substance Use Disorder in the Therapeutic Communities: Prevalence and Psychiatric Comorbidity. European Addiction Research. 2021;27(2):87-96. |
| Modestin J, Matutat B, Wurmle O. Antecedents of opioid dependence and personality disorder: attention-deficit/hyperactivity disorder and conduct disorder. European Archives of Psychiatry & Clinical Neuroscience. 2001;251(1):42-7. |
| Mol Debes NMM, Hjalgrim H, Skov L. Validation of the presence of comorbidities in a Danish clinical cohort of children with Tourette syndrome. Journal of Child Neurology. 2008;23(9):1017-27. |
| Moller LR, Sorensen MJ, Thomsen PH. ICD-10 classification in Danish child and adolescent psychiatry--Have diagnoses changed after the introduction of ICD-10? Nordic Journal of Psychiatry. 2007;61(1):71-8. |
| Moodley SV, Pillay AL. Two years of admissions to Natal's first inpatient child mental health centre. South African Medical Journal Suid-Afrikaanse Tydskrif Vir Geneeskunde. 1993;83(3):209-11. |
| Moon M, Lister J, Milosevic A, Ledgerwood D, Lister JJ, Ledgerwood DM. Subtyping Non-treatment-seeking Problem Gamblers Using the Pathways Model. Journal of Gambling Studies. 2017;33(3):841-53. |
| Mortberg E, Tilfors K, Bejerot S. Screening for ADHD in an adult social phobia sample. Journal of Attention Disorders. 2012;16(8):645-9. |
| Moura HF, Faller S, Benzano D, Szobot C, Von Diemen L, Stolf AR, et al. The effects of ADHD in adult substance abusers. Journal of Addictive Diseases. 2013;32(3):252-62. |
| Mukaddes NM, Hergner S, Tanidir C. Psychiatric disorders in individuals with high-functioning autism and Asperger's disorder: Similarities and differences. World Journal of Biological Psychiatry. 2010;11(8):964-71. |
| Mukaddes NM, Mutluer T, Ayik B, Umut A. What happens to children who move off the autism spectrum? Clinical follow-up study. Pediatrics International. 2017;59(4):416-21. |
| Myers WC, Burket RC, Otto TA. Conduct disorder and personality disorders in hospitalized adolescents. Journal of Clinical Psychiatry. 1993;54(1):21-6. |
| Nasser EH, Overholser JC. Assessing varying degrees of lethality in depressed adolescent suicide attempters. Acta Psychiatrica Scandinavica. 1999;99(6):423-31. |
| Noterdaeme M, Amorosa H. Evaluation of emotional and behavioral problems in language impaired children using the Child Behavior Checklist. European Child & Adolescent Psychiatry. 1999;8(2):71-7. |
| Ohlmeier MD, Peters K, Wildt BT, Zedler M, Ziegenbein M, Wiese B, et al. Comorbidity of alcohol and substance dependence with attention-deficit/hyperactivity disorder (ADHD). Alcohol and Alcoholism. 2008;43(3):300-4. |
| Olashore AA, Frank-Hatitchki B, Ogunwobi O. Diagnostic profiles and predictors of treatment outcome among children and adolescents attending a national psychiatric hospital in Botswana. Child & Adolescent Psychiatry & Mental Health [Electronic Resource]. 2017;11:8. |
| Ooi YP, Tan ZJ, Lim CX, Goh TJ, Sung M. Prevalence of behavioural and emotional problems in children with high-functioning autism spectrum disorders. Australian & New Zealand Journal of Psychiatry. 2011;45(5):370-5. |
| Ordonez AE, Loeb FF, Zhou X, Shora L, Berman RA, Broadnax DD, et al. Lack of Gender-Related Differences in Childhood-Onset Schizophrenia. Journal of the American Academy of Child & Adolescent Psychiatry. 2016;55(9):792-9. |
| Pachado MP, Scherer JN, Guimaraes LSP, von Diemen L, Pechansky F, Kessler FHP, de Almeida RMM. Markers for Severity of Problems in Interpersonal Relationships of Crack Cocaine Users from a Brazilian Multicenter Study. Psychiatric Quarterly. 2018;89(4):923-36. |
| Patel RS, Virani S, Saeed H, Nimmagadda S, Talukdar J, Youssef NA. Gender differences and comorbidities in U.S. Adults with bipolar disorder. Brain Sciences. 2018;8(9) (no pagination). |
| Peles E, Schreiber S, Sutzman A, Adelson M. Attention deficit hyperactivity disorder and obsessive-compulsive disorder among former heroin addicts currently in methadone maintenance treatment. Psychopathology. 2012;45(5):327-33. |
| Peralta V, de Jalón EG, Campos MS, Zandio M, Sanchez-Torres A, Cuesta MJ, et al. The meaning of childhood attention-deficit hyperactivity symptoms in patients with a first-episode of schizophrenia-spectrum psychosis. Schizophrenia Research. 2011;126(1-3):28-35. |
| Perugi G, Ceraudo G, Vannucchi G, Rizzato S, Toni C, Dell'Osso L. Attention deficit/hyperactivity disorder symptoms in Italian bipolar adult patients: a preliminary report. Journal of Affective Disorders. 2013;149(1-3):430-4. |
| Peter SC, Whelan JP, Ginley MK, Pfund RA, Wilson KK, Meyers AW. Disordered gamblers with and without ADHD: The role of coping in elevated psychological distress. International Gambling Studies. 2016;16(3):455-69. |
| Petersen MC, Kube DA, Whitaker TM, Graff JC, Palmer FB. Prevalence of developmental and behavioral disorders in a pediatric hospital. Pediatrics. 2009;123(3):e490-e5. |
| Philipsen A, Limberger MF, Lieb K, Feige B, Kleindienst N, Ebner-Priemer U, et al. Attention-deficit hyperactivity disorder as a potentially aggravating factor in borderline personality disorder. British Journal of Psychiatry. 2008;192(2):118-23. |
| Pianca TG, Rohde LA, Rosa RL, Begnis APA, Ferronatto PB, Jensen MC, et al. Crack cocaine use in adolescents: Clinical characteristics and predictors of early initiation. Journal of Clinical Psychiatry. 2016;77(10):e1205-e10. |
| Pinna M, Visioli C, Rago CM, Manchia M, Tondo L, Baldessarini RJ. Attention deficit-hyperactivity disorder in adult bipolar disorder patients. Journal of Affective Disorders. 2019;243:391-6. |
| Poyraz Findik OT, Ceri V, Unver H, Perdahli Fis N, Rodopman Arman A, Beser C, et al. Mental health need and psychiatric service utilization patterns of refugee children in Turkey: A comparative study. Children and Youth Services Review Vol 124 2021, ArtID 105970. 2021;124. |
| Pringsheim T, Hammer T. Social behavior and comorbidity in children with tics. Pediatric Neurology. 2013;49(6):406-10. |
| Prochaska JJ, Fromont SC, Delucchi K, Young-Wolff KC, Benowitz NL, Hall S, et al. Multiple risk-behavior profiles of smokers with serious mental illness and motivation for change. Health Psychology. 2014;33(12):1518-29. |
| Pulsifer BH, Evans CL, Capel L, Lyons-Hunter M, Grieco JA. Cross-sectional assessment of mental health and service disparities in a high-risk community. Translational Issues in Psychological Science. 2019;5(4):365-73. |
| Rad F, Buica A, Stancu M, Irimie-Ana A, Andrei E, Rosca D, Dobrescu I. Adult ADHD symptoms in a group of patients with substance abuse. Rivista di Psichiatria. 2020;55(3):161-7. |
| Ralph N, McMenamy C. Treatment outcomes in an adolescent chemical dependency program. Adolescence. 1996;31(121):91-107. |
| Raman N, Janse van Rensburg AB. Clinical and psycho-social profile of child and adolescent mental health care users and services at an urban child mental health clinic in South Africa. African Journal of Psychiatry. 2013;16(5):356-63. |
| Rawat VS, Dhiman V, Sinha S, Sagar KJV, Thippeswamy H, Chaturvedi SK, et al. Co-morbidities and outcome of childhood psychogenic non-epileptic seizures-An observational study. Seizure. 2015;25:95-8. |
| Regan T, Tubman J. Attention Deficit Hyperactivity Disorder (ADHD) Subtypes, Co-Occurring Psychiatric Symptoms and Sexual Risk Behaviors among Adolescents Receiving Substance Abuse Treatment. Substance Use & Misuse. 2020;55(1):119-32. |
| Retz W, Ringling J, Retz-Junginger P, Vogelgesang M, Rosler M. Association of attention-deficit/hyperactivity disorder with gambling disorder. Journal of Neural Transmission. 2016;123(8):1013-9. |
| Rey JM, Morris-Yates A, Singh M, Andrews G, Stewart GW. Continuities between psychiatric disorders in adolescents and personality disorders in young adults. American Journal of Psychiatry. 1995;152(6):895-900. |
| Rey JM. Comorbidity between disruptive disorders and depression in referred adolescents. Australian & New Zealand Journal of Psychiatry. 1994;28(1):106-13. |
| Reyes MM, Schneekloth TD, Hitschfeld MJ, Geske JR, Atkinson DL, Karpyak VM. The Clinical Utility of ASRS-v1.1 for Identifying ADHD in Alcoholics Using PRISM as the Reference Standard. Journal of Attention Disorders. 2019;23(10):1119-25. |
| Reyes MM, Schneekloth TD, Hitschfeld MJ, Karpyak VM. Impact of Sex and ADHD Status on Psychiatric Comorbidity in Treatment-Seeking Alcoholics. Journal of Attention Disorders. 2019;23(12):1505-13. |
| Rho A, Traicu A, Lepage M, Iyer SN, Malla A, Joober R. Clinical and functional implications of a history of childhood ADHD in first-episode psychosis. Schizophrenia Research. 2015;165(2-3):128-33. |
| Ribeiro SN, Jennen-Steinmetz C, Schmidt MH, Becker K. Nicotine and alcohol use in adolescent psychiatric inpatients: associations with diagnoses, psychosocial factors, gender and age. Nordic Journal of Psychiatry. 2008;62(4):315-21. |
| Richter M, Spangenberg H, Ramklint M, Ramirez A. The clinical relevance of asking young psychiatric patients about childhood ADHD symptoms. Nordic Journal of Psychiatry. 2020;74(1):23-9. |
| Rimal HS, Pokharel A. Prevalence of attention deficit hyperactivity disorder among school children and associated co-morbidities - a hospital based descriptive study. Kathmandu University Medical Journal. 2016;14(55):226-30. |
| Roberts N, Booij L, Axas N, Repetti L. Two-year prospective study of characteristics and outcome of adolescents referred to an adolescent urgent psychiatric clinic. International Journal of Adolescent Medicine and Health. 2018;30(1):1-5. |
| Roncero C, Ortega L, Perez-Pazos J, Lligona A, Abad AC, Gual A, et al. Psychiatric Comorbidity in Treatment-Seeking Alcohol Dependence Patients With and Without ADHD. Journal of Attention Disorders. 2019;23(12):1497-504. |
| Ross RG, Heinlein S, Tregellas H. High rates of comorbidity are found in childhood-onset schizophrenia. Schizophrenia Research. 2006;88(1-3):90-5. |
| Rounsaville BJ, Anton SF, Carroll K, Budde D, Prusoff BA, Gawin F. Psychiatric diagnoses of treatment-seeking cocaine abusers. Archives of General Psychiatry. 1991;48(1):43-51. |
| Rubino I, Frank E, Nanni RC, Pozzi D, di Scalea TL, Siracusano A. A comparative study of axis I antecedents before age 18 of unipolar depression, bipolar disorder and schizophrenia. Psychopathology. 2009;42(5):325-32. |
| Ruiz-Ramos D, Martinez-Magana JJ, Garcia AR, Juarez-Rojop IE, Gonzalez-Castro TB, Tovilla-Zarate CA, et al. Psychiatric Comorbidity in Mexican Adolescents with a Diagnosis of Eating Disorders Its Relationship with the Body Mass Index. International Journal of Environmental Research & Public Health [Electronic Resource]. 2021;18(8):08. |
| Ryden E, Thase M, Straht D, Aberg-Wistedt A, Bejerot S, Landen M. A history of childhood attention-deficit hyperactivity disorder (ADHD) impacts clinical outcome in adult bipolar patients regardless of current ADHD. Acta Psychiatrica Scandinavica. 2009;120(3):239-46. |
| Saba L, Byrne A, Mulligan A. Child art psychotherapy in CAMHS: Which cases are referred and which cases drop out? Springerplus. 2016;5(1):1816. |
| Safren SA, Lanka GD, Otto MW, Pollack MH. Prevalence of childhood ADHD among patients with generalized anxiety disorder and a comparison condition, social phobia. Depression and Anxiety. 2001;13(4):190-1. |
| Sagar R, Pattanayak RD, Mehta M. Clinical profile of mood disorders in children. Indian Pediatrics. 2012;49(1):21-3. |
| Sala L, Martinotti G, Carenti M, Romo L, Oumaya M, Pham-Scottez A, et al. Attention-deficit/hyperactivity disorder symptoms and psychological comorbidity in eating disorder patients. Eating and Weight Disorders. 2018;23(4):513-9. |
| Salous A, Al-Alem L, Omar HA. Trends in mental health of an adolescent medicine clinic patient population. International Journal of Adolescent Medicine & Health. 2009;21(1):9-14. |
| Samar SM, Moyano MB, Brana-Berrios M, Irazoqui G, Matos A, Kichic R, et al. Children and adolescents with Tourette's disorder in the USA versus Argentina: behavioral differences may reflect cultural factors. European Child & Adolescent Psychiatry. 2013;22(11):701-7. |
| Sanchez-Gistau V, Manzanares N, Cabezas A, Sole M, Algora MJ, Vilella E. Clinical and cognitive correlates of childhood attention-deficit/hyperactivity disorder in first-episode psychosis: A controlled study. European Neuropsychopharmacology. 2020;36:90-9. |
| Sathyabama R. Clinical characteristics and demographic profile of children with Autism Spectrum Disorder (ASD) at child development clinic (CDC), Penang Hospital, Malaysia. Medical Journal of Malaysia. 2019;74(5):372-6. |
| Say GN, Tasdemir HA, Akbas S, Yuce M, Karabekiroglu K. Self-esteem and psychiatric features of Turkish adolescents with psychogenic non-epileptic seizures: a comparative study with epilepsy and healthy control groups. International Journal of Psychiatry in Medicine. 2014;47(1):41-53. |
| Say GN, Tasdemir HA, Ince H. Semiological and psychiatric characteristics of children with psychogenic nonepileptic seizures: Gender-related differences. Seizure. 2015;31:144-8. |
| Schiff MM, Cavaiola AA. Teenage chemical dependence and the prevalence of psychiatric disorders: Issues for prevention. Journal of Adolescent Chemical Dependency. 1990;1(2):35-46. |
| Schubiner H, Tzelepis A, Milberger S, Lockhart N, Kruger M, Kelley BJ, Schoener EP. Prevalence of attention-deficit/hyperactivity disorder and conduct disorder among substance abusers. Journal of Clinical Psychiatry. 2000;61(4):244-51. |
| Seitz J, Kahraman-Lanzerath B, Legenbauer T, Sarrar L, Herpertz S, Salbach-Andrae H, et al. The role of impulsivity, inattention and comorbid ADHD in patients with bulimia nervosa. PLoS ONE [Electronic Resource]. 2013;8(5):e63891. |
| Seleem MA, Amer RA, Romeh AH, Hamoda HM. Demographic and clinical characteristics of children seeking psychiatric services in the Nile Delta region: an observational retrospective study. International Journal of Mental Health Systems. 2019;13:66. |
| Selles RR, Storch EA, Lewin AB. Variations in symptom prevalence and clinical correlates in younger versus older youth with obsessive-compulsive disorder. Child Psychiatry & Human Development. 2014;45(6):666-74. |
| Sentissi O, Navarro JC, Oliveira HD, Gourion D, Bourdel MC, Bayle FJ, et al. Bipolar disorders and quality of life: The impact of attention deficit/hyperactivity disorder and substance abuse in euthymic patients. Psychiatry Research. 2008;161(1):36-42. |
| Serra G, Iannoni ME, Trasolini M, Maglio G, Frattini C, Casini MP, et al. Characteristics associated with depression severity in 270 juveniles in a major depressive episode. Brain Sciences. 2021;11(4) (no pagination). |
| Shekunov J, Wozniak J, Conroy K, Pinsky E, Fitzgerald M, De Leon MF, et al. Prescribing patterns in a psychiatrically referred sample of youth with Autism spectrum disorder. Journal of Clinical Psychiatry. 2017;78(9):e1276-e83. |
| Sheppard B, Chavira D, Azzam A, Grados MA, Umaña P, Garrido H, et al. ADHD prevalence and association with hoarding behaviors in childhood-onset OCD. Depression & Anxiety (1091-4269). 2010;27(7):667-74. |
| Sidana A, Bhatia MS, Choudhary S. Prevalence and pattern of psychiatric morbidity in children. Indian Journal of Medical Sciences. 1998;52(12):556-8. |
| Siegel CE, Laska EM, Wanderling JA, Hernandez JC, Levenson RB. Prevalence and Diagnosis Rates of Childhood ADHD Among Racial-Ethnic Groups in a Public Mental Health System. Psychiatric Services. 2016;67(2):199-205. |
| Sitholey P, Agarwal V, Sharma S. An exploratory clinical study of adult attention deficit/hyperactivity disorder from India. Indian Journal of Medical Research. 2009;129(1):83-8. |
| Sivakumar T, Agarwal V, Sitholey P. Comorbidity of attention-deficit/hyperactivity disorder and bipolar disorder in North Indian clinic children and adolescents. Asian Journal of Psychiatry. 2013;6(3):235-42. |
| Skokauskas N, Dunne M, Gallogly A, Clark C. Ethnic minority populations and child psychiatry services: An Irish study. Children and Youth Services Review. 2010;32(10):1242-5. |
| Soutullo CA, DelBello MP, Ochsner JE, McElroy SL, Taylor SA, Strakowski SM, Keck Jr PE. Severity of bipolarity in hospitalized manic adolescents with history of stimulant or antidepressant treatment. Journal of Affective Disorders. 2002;70(3):323-7. |
| Soutullo CA, Escamilla-Canales I, Wozniak J, Gamazo-Garran P, Figueroa-Quintana A, Biederman J. Pediatric bipolar disorder in a Spanish sample: Features before and at the time of diagnosis. Journal of Affective Disorders. 2009;118(1-3):39-47. |
| Specker SM, Carlson GA, Christenson GA, Marcotte M. Impulse control disorders and attention deficit disorder in pathological gamblers. Annals of Clinical Psychiatry. 1995;7(4):175-9. |
| Speltz ML, McClellan J, DeKlyen M, Jones K. Preschool boys with oppositional defiant disorder: clinical presentation and diagnostic change. Journal of the American Academy of Child & Adolescent Psychiatry. 1999;38(7):838-45. |
| Speranza M, Revah-Levy A, Cortese S, Falissard B, Pham-Scottez A, Corcos M. ADHD in adolescents with borderline personality disorder. BMC Psychiatry. 2011;11:158. |
| Srinivasaraghavan R, Mahadevan S, Kattimani S. Impact of Comorbidity on Three Month Follow-up Outcome of Children with ADHD in a Child Guidance Clinic: Preliminary Report. Indian Journal of Psychological Medicine. 2013;35(4):346-51. |
| Srivastava V, Girdhar R, Verma P, Arya S, Sethi S. Pattern of child and adolescent mental disorders at a tertiary care centre in North India. Journal of Indian Association for Child and Adolescent Mental Health. 2021;17(4):152-65. |
| Stadnick N, Chlebowski C, Baker-Ericzen M, Dyson M, Garland A, Brookman-Frazee L. Psychiatric comorbidity in autism spectrum disorder: Correspondence between mental health clinician report and structured parent interview. Autism. 2017;21(7):841-51. |
| Staller JA, Wade MJ, Baker M. Current prescribing patterns in outpatient child and adolescent psychiatric practice in central New York. Journal of Child & Adolescent Psychopharmacology. 2005;15(1):57-61. |
| Staller JA. Diagnostic profiles in outpatient child psychiatry. American Journal of Orthopsychiatry. 2006;76(1):98-102. |
| Stargatt R, Crocker JH, Denton C. Prediction of aggression and restraint in child inpatient units. Australian & New Zealand Journal of Psychiatry. 2010;44(5):443-9. |
| Stephens JR, Heffner JL, Adler CM, Blom TJ, Anthenelli RM, Fleck DE, et al. Risk and protective factors associated with substance use disorders in adolescents with first-episode mania. Journal of the American Academy of Child & Adolescent Psychiatry. 2014;53(7):771-9. |
| Sterling S, Weisner C. Chemical dependency and psychiatric services for adolescents in private managed care: implications for outcomes. Alcoholism: Clinical & Experimental Research. 2005;29(5):801-9. |
| Sternat T, Fotinos K, Fine A, Epstein I, Katzman MA. Low hedonic tone and attention-deficit hyperactivity disorder: risk factors for treatment resistance in depressed adults. Neuropsychiatric Disease & Treatment. 2018;14:2379-87. |
| Stewart MA, Cummings C, Singer S, DeBlois C. The overlap between hyperactive and unsocialized aggressive children. Child Psychology & Psychiatry & Allied Disciplines. 1981;22(1):35-45. |
| Stickley A, Tachimori H, Inoue Y, Shinkai T, Yoshimura R, Nakamura J, et al. Attention-deficit/hyperactivity disorder symptoms and suicidal behavior in adult psychiatric outpatients. Psychiatry & Clinical Neurosciences. 2018;72(9):713-22. |
| Storch EA, Merlo LJ, Larson MJ, Geffken GR, Lehmkuhl HD, Jacob ML, et al. Impact of comorbidity on cognitive-behavioral therapy response in pediatric obsessive-compulsive disorder. Journal of the American Academy of Child & Adolescent Psychiatry. 2008;47(5):583-92. |
| Stralin P, Hetta J. First episode psychosis and comorbid ADHD, autism and intellectual disability. European Psychiatry: the Journal of the Association of European Psychiatrists. 2019;55:18-22. |
| Svedlund NE, Norring C, Ginsberg Y, Hausswolff-Juhlin Y. Are treatment results for eating disorders affected by ADHD symptoms? A one-year follow-up of adult females. European Eating Disorders Review. 2018;26(4):337-45. |
| Svedlund NE, Norring C, Ginsberg Y, von Hausswolff-Juhlin Y. Symptoms of Attention Deficit Hyperactivity Disorder (ADHD) among adult eating disorder patients. BMC Psychiatry. 2017;17(1):19. |
| Syed EU, Hussein SA, Yousafzai AW. Developing Services with Limited Resources: Establishing a CAMHS in Pakistan. Child & Adolescent Mental Health. 2007;12(3):121-4. |
| Syed H, Masaud TM, Nkire N, Iro C, Garland MR. Estimating the prevalence of adult ADHD in the psychiatric clinic: a cross-sectional study using the adult ADHD self-report scale (ASRS). Irish Journal of Psychological Medicine. 2010;27(4):195-7. |
| Taich A, Crowe S, Kosmorsky GS, Traboulsi EI. Prevalence of psychosocial disturbances in children with nonorganic visual loss. Journal of AAPOS. 2004;8(5):457-61. |
| Tamam L, Tuglu C, Karatas G, Ozcan S. Adult attention-deficit hyperactivity disorder in patients with bipolar I disorder in remission: preliminary study. Psychiatry & Clinical Neurosciences. 2006;60(4):480-5. |
| Tamburin S, Federico A, Morbioli L, Faccini M, Casari R, Zamboni L, et al. Screening for adult attention deficit/hyperactivity disorder in high-dose benzodiazepine dependent patients. American Journal on Addictions. 2017;26(6):610-4. |
| Tang Y-L, Kranzler HR, Gelernter J, Farrer LA, Cubells JF. Comorbid psychiatric diagnoses and their association with cocaine-induced psychosis in cocaine-dependent subjects. The American Journal on Addictions. 2007;16(5):343-51. |
| Tani P, Lindberg N, Appelberg B, Nieminen-von Wendt T, von Wendt L, Porkka-Heiskanen T. Childhood inattention and hyperactivity symptoms self-reported by adults with Asperger syndrome. Psychopathology. 2006;39(1):49-54. |
| Tejeda‐Romero C, Kobashi‐Margáin RA, Alvarez‐Arellano L, Corona JC, González‐García N, Tejeda-Romero C, et al. Differences in substance use, psychiatric disorders and social factors between Mexican adolescents and young adults. American Journal on Addictions. 2018;27(8):625-31. |
| Testa G, Baenas I, Vintro-Alcaraz C, Granero R, Aguera Z, Sanchez I, et al. Does ADHD symptomatology influence treatment outcome and dropout risk in eating disorders? A longitudinal study. Journal of Clinical Medicine. 2020;9(7):1-11. |
| Thomson K, Randall E, Ibeziako P, Bujoreanu IS. Somatoform Disorders and Trauma in Medically-Admitted Children, Adolescents, and Young Adults: Prevalence Rates and Psychosocial Characteristics. Psychosomatics. 2014;55(6):630-9. |
| Tiwari R, Agarwal V, Arya A, Gupta PK, Mahour P. An exploratory clinical study of disruptive mood dysregulation disorder in children and adolescents from India. Asian Journal of Psychiatry. 2016;21:37-40. |
| Torok M, Darke S, Kaye S. Attention deficit hyperactivity disorder and severity of substance use: the role of comorbid psychopathology. Psychology of Addictive Behaviors. 2012;26(4):974-9. |
| Torres I, Gomez N, Colom F, Jimenez E, Bosch R, Bonnin C, et al. Bipolar disorder with comorbid attention-deficit and hyperactivity disorder. Main clinical features and clues for an accurate diagnosis. Acta Psychiatrica Scandinavica. 2015;132(5):389-99. |
| Tramontina S, Schmitz M, Polanczyk G, Rohde LA. Juvenile bipolar disorder in Brazil: clinical and treatment findings. Biological Psychiatry. 2003;53(11):1043-9. |
| Ubhi M, Achinivu K, Seri S, Cavanna AE. Motor stereotypies in adult patients with Tourette syndrome. Future Neurology. 2020;15(2) (no pagination). |
| Unver H, Karakaya I. The Assessment of the Relationship Between ADHD and Posttraumatic Stress Disorder in Child and Adolescent Patients. Journal of Attention Disorders. 2019;23(8):900-3. |
| Valsecchi P, Nibbio G, Rosa J, Tamussi E, Turrina C, Sacchetti E, Vita A. Adult ADHD: Prevalence and Clinical Correlates in a Sample of Italian Psychiatric Outpatients. Journal of Attention Disorders. 2018 Dec 20;25(4):530-9. |
| Van Ameringen M, Mancini C, Simpson W, Patterson B. Adult attention deficit hyperactivity disorder in an anxiety disorders population. CNS Neuroscience & Therapeutics. 2011;17(4):221-6. |
| van de Glind G, van den Brink W, Koeter MW, Carpentier P-J, van Emmerik-van Oortmerssen K, Kaye S, et al. Validity of the Adult ADHD Self-Report Scale (ASRS) as a screener for adult ADHD in treatment seeking substance use disorder patients. Drug and Alcohol Dependence. 2013;132(3):587-96. |
| van Emmerik-van Oortmerssen K, van de Glind G, Koeter MW, Allsop S, Auriacombe M, Barta C, et al. Psychiatric comorbidity in treatment-seeking substance use disorder patients with and without attention deficit hyperactivity disorder: Results of the IASP study. Addiction. 2014;109(2):262-72. |
| Van Hout M, Foley M. Attention-deficit hyperactivity disorder (ADHD) in adults attending addiction treatment in Ireland: Preliminary international ADHD in substance-use disorders prevalence study (IASP) results. Irish Journal of Psychological Medicine. 2013;30(3):171-7. |
| Vanbronkhorst SB, Roberts DE, Edwards EM, Blankenship K. Diagnosis and Use of Psychotherapy Among Children and Adolescents Prescribed Antipsychotics. Journal of Psychiatric Practice. 2018;24(5):323-30. |
| Vasa RA, Kalb L, Mazurek M, Kanne S, Freedman B, Keefer A, et al. Age-related differences in the prevalence and correlates of anxiety in youth with autism spectrum disorders. Research in Autism Spectrum Disorders. 2013;7(11):1358-69. |
| Venkatesh C, Ravikumar T, Andal A, Virudhagirinathan BS. Attention-deficit/Hyperactivity Disorder in Children: Clinical Profile and Co-morbidity. Indian Journal of Psychological Medicine. 2012;34(1):34-8. |
| Vergara-Moragues E, Gonzalez-Saiz F, Lozano OM, Garcia AV. Psychiatric profile of three-month retention in cocaine-dependent patients treated in a therapeutic community. Journal of Studies on Alcohol and Drugs. 2013;74(3):452-9. |
| Vergara-Moragues E, Gonzalez-Saiz F, Lozano Rojas O, Bilbao Acedos I, Fernandez Calderon F, Betanzos Espinosa P, et al. Diagnosing adult attention deficit/hyperactivity disorder in patients with cocaine dependence: discriminant validity of Barkley executive dysfunction symptoms. European Addiction Research. 2011;17(6):279-84. |
| Vuijk PJ, Lee BA, Ditmars HL, Samkavitz AR, Lind HS, Doyle AE, et al. Characteristics of Child Psychiatric Outpatients at Highest Risk for Suicidal Thoughts and Behaviors. Child Psychiatry & Human Development. 2019;50(3):505-19. |
| Waluk O, Youssef G, Dowling N. The relationship between problem gambling and attention deficit hyperactivity disorder. Journal of Gambling Studies. 2016;32(2):591-604. |
| Wamithi S, Ochieng R, Njenga F, Akech S, Macharia WM. Cross-sectional survey on prevalence of attention deficit hyperactivity disorder symptoms at a tertiary care health facility in Nairobi. Child & Adolescent Psychiatry & Mental Health [Electronic Resource]. 2015;9(1):1. |
| Wamulugwa J, Kakooza A, Kitaka SB, Nalugya J, Kaddumukasa M, Moore S, et al. Prevalence and associated factors of attention deficit hyperactivity disorder (ADHD) among Ugandan children; a cross-sectional study. Child & Adolescent Psychiatry & Mental Health [Electronic Resource]. 2017;11:18. |
| Wapp M, van de Glind G, van Emmerik-van Oortmerssen K, Dom G, Verspreet S, Carpentier PJ, et al. Risk factors for borderline personality disorder in treatment seeking patients with a substance use disorder: An international multicenter study. European Addiction Research. 2015;21(4):188-94. |
| Waseem M, Prasankumar R, Pagan K, Leber M. A retrospective look at length of stay for pediatric psychiatric patients in an urban emergency department. Pediatric Emergency Care. 2011;27(3):170-3. |
| Watanabe-Galloway S, Rieke K, Corley B, Valleley R. Behavioral Health Problems Presented to Integrated Pediatric Behavioral Health Clinics: Differences in Urban and Rural Patients. Community Mental Health Journal. 2017;53(1):27-33. |
| Weber EB, Studeny J, Kavanaugh BC, Cook NE, Gaudet CE, McCurdy K, Holler KA. Pediatric depression symptoms, executive functioning weaknesses, and associated neuropsychological and psychiatric outcomes. Journal of Child and Family Studies. 2018;27(5):1661-70. |
| Weibel S, Nicastro R, Prada P, Cole P, Rufenacht E, Pham E, et al. Screening for attention-deficit/hyperactivity disorder in borderline personality disorder. Journal of Affective Disorders. 2018;226:85-91. |
| Weintraub MJ, Schneck CD, Walshaw PD, Chang KD, Singh MK, Axelson DA, et al. Characteristics of youth at high risk for bipolar disorder compared to youth with bipolar I or II disorder. Journal of Psychiatric Research. 2020;123:48-53. |
| Weintraub MJ, Youngstrom EA, Marvin SE, Podell JL, Walshaw PD, Kim EY, et al. Diagnostic profiles and clinical characteristics of youth referred to a pediatric mood disorders clinic. Journal of Psychiatric Practice. 2014;20(2):154-62. |
| Welch E, Ghaderi A, Swenne I. A comparison of clinical characteristics between adolescent males and females with eating disorders. BMC Psychiatry. 2015;15:45. |
| Wentz E, Lacey JH, Waller G, Råstam M, Turk J, Gillberg C. Childhood onset neuropsychiatric disorders in adult eating disorder patients: a pilot study. European Child & Adolescent Psychiatry. 2005;14(8):431-7. |
| Wesemann D. Decreasing Rates of Pediatric Bipolar Within an Outpatient Practice. Journal of Child & Adolescent Psychiatric Nursing. 2016;29(4):188-95. |
| West SA, Strakowski SM, Sax KW, McElroy SL, Keck PE, McConville BJ. Phenomenology and comorbidity of adolescents hospitalized for the treatment of acute mania. Biological Psychiatry. 1996;39(6):458-60. |
| West SL, Mulsow M, Arredondo R. An examination of the psychometric properties of the attention deficit scales for adults with outpatient substance abusers. American Journal of Drug & Alcohol Abuse. 2007;33(5):755-64. |
| Wise BK, Cuffe SP, Fischer T. Dual diagnosis and successful participation of adolescents in substance abuse treatment. Journal of Substance Abuse Treatment. 2001;21(3):161-5. |
| Wodrich DL, Benjamin E, Lachar D. Tourette's syndrome and psychopathology in a child psychiatry setting. Journal of the American Academy of Child & Adolescent Psychiatry. 1997;36(11):1618-24. |
| Wright HH, Batey SR, Buttefield PT, Harris EC. The changing spectrum of children and adolescents seen in a child and adolescent psychiatric clinic during the 1970's. Psychiatric Forum. 1989;15(1):11-8. |
| Wu L-T, Gersing K, Burchett B, Woody GE, Blazer DG. Substance use disorders and comorbid axis I and II psychiatric disorders among young psychiatric patients: Findings from a large electronic health records database. Journal of Psychiatric Research. 2011;45(11):1453-62. |
| Yager J. Can trans-syndromal prototypes ("types") improve depiction of complex psychiatric cases?: An alternative way to consider concordant comorbid psychiatric disorders and their contexts as coherent units for research, assessment, and treatment planning. Journal of Nervous and Mental Disease. 2021;209(1):1-8. |
| Yar A, Gundogdu OY, Tural U, Cakin Memik N. The prevalence of internet addiction in turkish adolescents with psychiatric disorders. Noropsikiyatri Arsivi. 2019;56(3):200-4. |
| Yates WR, Lund BC, Johnson C, Mitchell J, McKee P. Attention-deficit hyperactivity symptoms and disorder in eating disorder inpatients. International Journal of Eating Disorders. 2009;42(4):375-8. |
| Yellowlees PM, Hilty DM, Marks SL, Neufeld J, Bourgeois JA, Yellowlees PM, et al. A retrospective analysis of a child and adolescent eMental Health program. Journal of the American Academy of Child & Adolescent Psychiatry. 2008;47(1):103-7. |
| Yen J-Y, Liu T-L, Wang P-W, Chen C-S, Yen C-F, Ko C-H. Association between Internet gaming disorder and adult attention deficit and hyperactivity disorder and their correlates: Impulsivity and hostility. Addictive Behaviors. 2017;64:308-13. |
| Yildirim B, Fis NP, Akgul GY, Ayaz AB. Gender dysphoria and attention problems: Possible clue for biological underpinnings. Psychiatry and Clinical Psychopharmacology. 2017;27(3):289-96. |
| Yildiz D, Ciftci A, Yalcin O. Substance use patterns, psychosocial traumas, psychiatric comorbidities, and gender differences among adolescent inpatients at an addiction treatment center. Dusunen Adam. 2020;33(2):190-202. |
| Yoshida Y, Uchiyama T. The clinical necessity for assessing Attention Deficit/Hyperactivity Disorder (AD/HD) symptoms in children with high-functioning Pervasive Developmental Disorder (PDD). European Child & Adolescent Psychiatry. 2004;13(5):307-14. |
| Yule AM, DiSalvo M, Biederman J, Wilens TE, Dallenbach NT, Taubin D, Joshi G. Decreased risk for substance use disorders in individuals with high-functioning autism spectrum disorder. European Child & Adolescent Psychiatry. 2021;07:07. |
| Zahid S, Bodicherla KP, Eskander N, Patel RS. Attention-Deficit/Hyperactivity Disorder and Suicidal Risk in Major Depression: Analysis of 141,530 Adolescent Hospitalizations. Cureus. 2020;12(5):e7949. |
| Zanarini MC, Athanasiadi A, Temes CM, Magni LR, Hein KE, Fitzmaurice GM, et al. Symptomatic Disorders in Adults and Adolescents With Borderline Personality Disorder. Journal of Personality Disorders. 2021;35(Supplement B):48-55. |
| Ziedonis DM, Rayford BS, Bryant KJ, Rounsaville BJ. Psychiatric comorbidity in white and African-American cocaine addicts seeking substance abuse treatment. Hospital & Community Psychiatry. 1994;45(1):43-9. |
| Zima BT, Rodean J, Hall M, Bardach NS, Coker TR, Berry JG. Psychiatric Disorders and Trends in Resource Use in Pediatric Hospitals. Pediatrics. 2016;138(5):11. |
| Zimmerman M, Gorlin E, Dalrymple K, Chelminiski I. A clinically useful screen for attention-deficit/hyperactivity disorder in adult psychiatric outpatients. Annals of Clinical Psychiatry. 2017;29(3):160-6. |
| Zinna S, Luxton R, Papachristou E, Dima D, Kyriakopoulos M. Comorbid chronic tic disorder and Tourette syndrome in children requiring inpatient mental health treatment. Clinical child psychology and psychiatry. 2021;26(3):894-905. |
|  |
| **Phase 2 (n= 82)** |
| Adamis D, Flynn C, Wrigley M, Gavin B, McNicholas F. ADHD in Adults: A Systematic Review and Meta-Analysis of Prevalence Studies in Outpatient Psychiatric Clinics. Journal of Attention Disorders. 2022;26(12). |
| Adamis D, Fox N, de M de Camargo APP, Saleem F, Gavin B, McNicholas F. Prevalence of attention deficit hyperactivity disorder in an adult mental health service in the Republic of Ireland. International Journal of Psychiatry in Medicine. 2023;58(2). |
| Anitha FS, Narasimhan U, Janakiraman A, Janakarajan N, Tamilselvan P. Association of digital media exposure and addiction with child development and behavior: A cross-sectional study. Industrial Psychiatry Journal. 2021;30(2). |
| Aydin S, Crone MR, Siebelink BM, Numans ME, Vermeiren RRJM, Westenberg PM. Informative value of referral letters from general practice for child and adolescent mental healthcare. European Child & Adolescent Psychiatry. 2023;32(2). |
| Badrfam R, Zandifar A, Barkhori Mehni M, Farid M, Rahiminejad F. Comorbidity of adult ADHD and substance use disorder in a sample of inpatients bipolar disorder in Iran. BMC Psychiatry. 2022;22(1). |
| Baizabal-Carvallo JF, Jankovic J. Sex differences in patients with Tourette syndrome. CNS Spectr. 2022:1-7. |
| Bannett Y, Dahlen A, Huffman LC, Feldman HM. Primary Care Diagnosis and Treatment of Attention-Deficit/Hyperactivity Disorder in School-Age Children: Trends and Disparities During the COVID-19 Pandemic. Journal of Developmental & Behavioral Pediatrics. 2022;43(7). |
| Bartoli F, Callovini T, Cavaleri D, Cioni RM, Bachi B, Calabrese A, et al. Clinical correlates of comorbid attention deficit hyperactivity disorder in adults suffering from bipolar disorder: A meta-analysis. Australian & New Zealand Journal of Psychiatry. 2023;57(1). |
| Bassiony MM, Salah El‐Deen GM, Ameen N, Mahdy RS. Prevalence, correlates, and consequences of attention‐deficit/hyperactivity disorder in a clinical sample of adults with tramadol use in Egypt. American Journal on Addictions. 2022;31(1). |
| Berg L, Pringsheim T, Martino D. Etiological factors associated with functional tic like behaviours during the COVID-19 pandemic. Neurology Conference: American Academy Of Neurology Annual Meeting, AAN. 2023;100(17 Supplement 2). |
| Beverly J, Giannouchos T, Callaghan T. Examining frequent emergency department use among children and adolescents with autism spectrum disorder. Autism: The International Journal of Research & Practice. 2021;25(5). |
| Beyoglu R, Erdur B. Evaluation of the Relationship Between Head Trauma and Attention-Deficit/Hyperactivity Disorder in Primary School Children Admitted to the Emergency Department. Pediatric Emergency Care. 2022;38(11). |
| Bezborodovs N, Kocane A, Rancans E, Villerusa A. Clinical Utility of the Parent-Report Version of the Strengths and Difficulties Questionnaire (SDQ) in Latvian Child and Adolescent Psychiatry Practice. Medicina. 2022;58(11). |
| Bjork JM, Shull ER, Perrin PB, Shura RD. Suicidal ideation and clinician-rated suicide risk in veterans referred for ADHD evaluation at a VA Medical Center. Psychological Services. 2022;14. |
| Bonati M, Scarpellini F, Cartabia M, Zanetti M. Ten Years (2011–2021) of the Italian Lombardy ADHD Register for the Diagnosis and Treatment of Children and Adolescents with ADHD. Children. 2021;8(7). |
| Brandt L, Levin FR, Kraigher D. Impulsive Personality Traits Mediate the Relationship Between Attention-Deficit/Hyperactivity Disorder Symptoms and Psychiatric Comorbidity among Patients with Severe Alcohol Use Disorder. Journal of Dual Diagnosis. 2021;17(3). |
| Buica AM, Preda DM, Andrei LE, Stancu M, Gica N, Rad F. Maladaptive Personality Traits in a Group of Patients with Substance Use Disorder and ADHD. Medicina. 2022;58(7). |
| Carbone JT, Kremer KP, Holzer KJ, Kondis JS, Vaughn MG. Emergency Department Admissions for Physical Child Abuse: Evidence from the 2006-2017 Nationwide Emergency Department Sample. Journal of Interpersonal Violence. 2022;37(15/16). |
| Caye A, Agnew-Blais J, Arseneault L, Goncalves H, Kieling C, Langley K, et al. "A risk calculator to predict adult attention-deficit/hyperactivity disorder: Generation and external validation in three birth cohorts and one clinical sample": Erratum. Epidemiology and Psychiatric Sciences Vol 29 2020, ArtID e41. 2020;29. |
| Caye A, Agnew-Blais J, Arseneault L, Goncalves H, Kieling C, Langley K, et al. A risk calculator to predict adult attention-deficit/hyperactivity disorder: Generation and external validation in three birth cohorts and one clinical sample. Epidemiology and Psychiatric Sciences Vol 29 2020, ArtID e37. 2020;29. |
| Cerny BM, Reynolds TP, Chang F, Scimeca LM, Phillips MS, Ogram Buckley CM, et al. Cognitive Performance and Psychiatric Self-Reports Across Adult Cognitive Disengagement Syndrome and ADHD Diagnostic Groups. Journal of Attention Disorders. 2023;27(3). |
| Coetzee C, Truter I, Meyer A. Differences in alcohol and cannabis use amongst substance use disorder patients with and without comorbid attention-deficit/hyperactivity disorder. The South African Journal Of Psychiatry : SAJP : The Journal Of The Society Of Psychiatrists Of South Africa. 2022;28. |
| Colgan B, Ouyang B, Gera A, Kompoliti A. Effects of Videogaming on Attention and Conduct in Adolescents with Tourette ' s Syndrome. Neurology Conference: American Academy Of Neurology Annual Meeting, AAN. 2023;100(17 Supplement 2). |
| Convertino AD, Blashill AJ. Psychiatric comorbidity of eating disorders in children between the ages of 9 and 10. Journal of Child Psychology & Psychiatry & Allied Disciplines. 2022;63(5). |
| Cook NE, Iaccarino MA, Karr JE, Iverson GL. Attention-deficit/hyperactivity disorder and outcome after concussion: A systematic review. Journal of Developmental and Behavioral Pediatrics. 2020;41(7). |
| Cook NE, Teel E, Iverson GL, Friedman D, Grilli L, Gagnon I. Attention-Deficit/Hyperactivity Disorder and Outcome from Concussion: Examining Duration of Active Rehabilitation and Clinical Recovery. Physical & Occupational Therapy in Pediatrics. 2022;42(6). |
| Cornell E, Blanchard A, Chihuri S, DiGuiseppi CG, Li G. Poisoning-related emergency department visits in children with autism spectrum disorder. Injury Epidemiology. 2022;9(1). |
| Cost KT, Crosbie J, Anagnostou E, Birken CS, Charach A, Monga S, et al. Mostly worse, occasionally better: impact of COVID-19 pandemic on the mental health of Canadian children and adolescents. European Child & Adolescent Psychiatry. 2022;31(4). |
| Cummings AD, Van Horne B, Correa N, Schwarzwald H, Chapman S. Can Pediatric Primary Care Practices Afford Integrated Behavioral Health? A Comparison of 5 Pediatric Practices. Clinical Pediatrics. 2022;61(12). |
| Dy-Hollins M, Chibnik L, Osiecki L, Sharma N, Mathews C, Scharf J. Assessing Sex Differences in Individuals with Tourette Syndrome and Persistent Motor or Vocal Tic Disorder. Neurology Conference: American Academy Of Neurology Annual Meeting, AAN. 2023;100(17 Supplement 2). |
| Edinoff AN, Apgar TL, Rogers JJ, Harper JD, Cornett EM, Kaye AM, et al. Attention Deficit Hyperactivity Disorder and Bipolar Disorder: Diagnosis, Treatments, and Clinical Considerations: A Narrative Review. Psychiatry International. 2022;3(1). |
| El Archi S, Barrault S, Garcia M, Branger S, Maugé D, Ballon N, et al. Adult ADHD Diagnosis, Symptoms of Impulsivity, and Emotional Dysregulation in a Clinical Sample of Outpatients Consulting for a Behavioral Addiction. Journal of Attention Disorders. 2023;27(7). |
| Eseaton PO, Nwaobi S, Onyeaka H, Oladunjoye A, Enosolease A, Edigin E, et al. Prevalence of Substance Use Disorder in Hospitalized Pediatric Patients With Eating Disorders: Analysis of the Kids Inpatient Database. American Journal on Addictions. 2023;32(2). |
| Fahrendorff AM, Pagsberg AK, Kessing LV, Maigaard K. Psychiatric comorbidity in patients with pediatric bipolar disorder - A systematic review. Acta Psychiatrica Scandinavica. 2023;148(2). |
| Federico A, Mantovani E, Casari R, Bertoldi A, Lugoboni F, Tamburin S. Adult attention-deficit/hyperactivity disorder symptoms, cognitive dysfunction and quality of life in high-dose use of benzodiazepine and Z-drug. Journal of Neural Transmission. 2021;128(7). |
| Ford JD, Spinazzola J, van der Kolk B, Chan G. Toward an empirically based Developmental Trauma Disorder diagnosis and semi-structured interview for children: The DTD field trial replication. Acta Psychiatrica Scandinavica. 2022;145(6). |
| Garcia-Delgar B, Servera M, Coffey BJ, Lázaro L, Openneer T, Benaroya-Milshtein N, et al. Tic disorders in children and adolescents: does the clinical presentation differ in males and females? A report by the EMTICS group. European Child & Adolescent Psychiatry. 2022;31(10). |
| Gardvik KS, Rygg M, Torgersen T, Wallander JL, Lydersen S, Indredavik MS. Association of treatment procedures and resilience to symptom load three-years later in a clinical sample of adolescent psychiatric patients. BMC Psychiatry. 2021;21(1). |
| Gerhand S, Saville CWN. ADHD prevalence in the psychiatric population. International Journal of Psychiatry in Clinical Practice. 2022;26(2). |
| Girgis J, Martino D, Pringsheim T. Influence of sex on tic severity and psychiatric comorbidity profile in patients with pediatric tic disorder. Developmental Medicine & Child Neurology. 2022;64(4). |
| Herd T, Palmer L, Font S. Prevalence of Mental Health Diagnoses Among Early Adolescents Before and During Foster Care. Journal of Developmental & Behavioral Pediatrics. 2023;44(4). |
| High P, Silver EJ, Stein REK, Roizen N, Augustyn M, Blum N. Do Referral Factors Predict a Probable Autism Spectrum Disorder Diagnosis? A DBPNet Study. Academic Pediatrics. 2022;22(2). |
| Hinshaw SP, Nguyen PT, O'Grady SM, Rosenthal EA. Annual Research Review: Attention-deficit/hyperactivity disorder in girls and women: underrepresentation, longitudinal processes, and key directions. Journal of Child Psychology & Psychiatry & Allied Disciplines. 2022;63(4). |
| Jarbin H, Höglund K, Skarphedinsson G, Bremander A. Aerobic exercise for adolescent outpatients with persistent major depression: Feasibility and acceptability of moderate to vigorous group exercise in a clinically referred sample. Clinical Child Psychology & Psychiatry. 2021;26(4). |
| Kammer PV, Moro JS, Soares JP, Massignan C, Phadraig CMG, Bolan M. Prevalence of tooth grinding in children and adolescents with neurodevelopmental disorders: A systematic review and meta-analysis. Journal of Oral Rehabilitation. 2022;49(6). |
| Kawata T, Sugihara G, Kakibuchi Y, Tomitaka M, Miyajima M, Matsushima E, et al. Attention-deficit hyperactivity symptoms and risk of alcohol use relapse. Neuropsychopharmacology Reports. 2023;43(1). |
| Khadr S, Masic U, Clarke V, Lynn RM, Holt V, Carmichael P. Key socio-demographic characteristics of children and adolescents with gender dysphoria: A British Isles surveillance study. Clinical Child Psychology & Psychiatry. 2022;27(4). |
| Kola S, Larson D. Gender Identity and Treatment Response in Patients with Functional Tic-Like Behavior. Movement Disorders. 2022;37(Supplement 2). |
| Lino F, Chieffo DPR. Developmental Coordination Disorder and Most Prevalent Comorbidities: A Narrative Review. Children. 2022;9(7). |
| Liu F, Wang G, Ye J, Yao B, Wang J, Wang H, et al. Sociodemographic and clinical characteristics of children with tic disorders and behavioral problems: A real-world study and development of a prediction model. BMC Pediatrics. 2023;23(1) (no pagination). |
| Luk JW, Ramchandani VA, Diazgranados N, Schwandt ML, Gunawan T, George DT, et al. Multidimensional Quality of Life Across the Spectrum of Alcohol Use Behavior. Psychiatric Research & Clinical Practice. 2022;4(4). |
| Marr M, Horwitz SM, Gerson R, Storfer-Isser A, Havens JF. Friendly Faces: Characteristics of Children and Adolescents with Repeat Visits to a Specialized Child Psychiatric Emergency Program. Pediatric Emergency Care. 2021;37(1). |
| May T, Aizenstros A, Aizenstros J. Characteristics of adults with attention deficit hyperactivity disorder consecutively referred to an Australian private psychiatric clinic. Australian Psychologist. 2022;57(2). |
| Moss CM, Metzger KB, Carey ME, Blum NJ, Curry AE, Power TJ. Chronic Care for Attention-Deficit/Hyperactivity Disorder: Clinical Management from Childhood Through Adolescence. Journal of developmental and behavioral pediatrics : JDBP. 2020;41 Suppl 2S. |
| Mroczkowski MM, Walkup JT, Appelbaum PS. Assessing Violence Risk in Adolescents in the Pediatric Emergency Department: Systematic Review and Clinical Guidance. Western Journal of Emergency Medicine: Integrating Emergency Care with Population Health. 2021;22. |
| Mucci M, Lenzi F, D'Acunto GM, Gazzillo M, Accorinti I, Boldrini S, et al. How COVID-19 Phases Have Impacted Psychiatric Risk: A Retrospective Study in an Emergency Care Unit for Adolescents. Children. 2022;9(12). |
| Nilles C, Fletcher J, Martino D, Pringsheim T. An exploration of tic phenomenology in children with primary tic disorders. Movement Disorders. 2022;37(Supplement 2). |
| Nilles C, Roze E, Hartmann A, Worbe Y, Bendetowicz D, Szejko N, et al. Substance misuse in adults with primary tic disorders. Neurology Conference: American Academy Of Neurology Annual Meeting, AAN. 2023;100(17 Supplement 2). |
| Olsson P, Wiktorsson S, Stromsten LM, Salander Renberg E, Runeson B, Waern M. Attention deficit hyperactivity disorder in adults who present with self-harm: A comparative 6-month follow-up study. BMC Psychiatry Vol 22 2022, ArtID 428. 2022;22. |
| Pakyurek M, Badawy M, Ugalde IT, Ishimine P, Chaudhari PP, McCarten-Gibbs K, et al. Does attention-deficit/hyperactivity disorder increase the risk of minor blunt head trauma in children? Journal of Child & Adolescent Psychiatric Nursing. 2022;35(4). |
| Paruk ME, Nassen R. Cyberbullying perpetration and victimisation amongst adolescent psychiatric patients at Lentegeur Hospital, South Africa. The South African Journal Of Psychiatry : SAJP : The Journal Of The Society Of Psychiatrists Of South Africa. 2022;28. |
| Ragusa A, Ganti L, Vera AE, Gue S. Pediatric Psychiatric Holds in the Emergency Department. Academic Emergency Medicine. 2023;30(Supplement 1). |
| Roest S, Siebelink B, van Ewijk H, Vermeiren R, Middeldorp C, van der Lans R. Sociodemographic and clinical characteristics in child and youth mental health; comparison of routine outcome measurements of an Australian and Dutch outpatient cohort. Epidemiology and Psychiatric Sciences Vol 30 2021, ArtID e74. 2021;30. |
| Sadeghian Nadooshan MR, Shahrivar Z, Mahmoudi Gharaie J, Salehi L. ADHD in adults with major depressive or bipolar disorder: does it affect clinical features, comorbidity, quality of life, and global functioning? BMC Psychiatry. 2022;22(1). |
| Schachar RJ, Dupuis A, Anagnostou E, Georgiades S, Soreni N, Arnold PD, et al. Obsessive-compulsive disorder in children and youth: neurocognitive function in clinic and community samples. Journal of Child Psychology & Psychiatry & Allied Disciplines. 2022;63(8). |
| Schott W, Sha T, Shea L. Emergency Visits for Autistic Children and Children With ADHD. Pediatrics. 2022;149. |
| Schulte-Ruther M, Kulvicius T, Stroth S, Wolff N, Roessner V, Marschik PB, et al. Using machine learning to improve diagnostic assessment of ASD in the light of specific differential and co-occurring diagnoses. Journal of Child Psychology & Psychiatry & Allied Disciplines. 2023;64(1). |
| Seleem MA, Amer RA. Demographic & clinical correlates of admission into a specialized psychiatric inpatient service for children and adolescents in Egypt: An observational retrospective study. Journal of Psychiatric & Mental Health Nursing (John Wiley & Sons, Inc). 2021;28(6). |
| Sheldrick RC, Bair-Merritt MH, Durham MP, Rosenberg J, Tamene M, Bonacci C, et al. Integrating Pediatric Universal Behavioral Health Care at Federally Qualified Health Centers. Pediatrics. 2022;149(4). |
| Shkalim Zemer V, Hoshen M, Gerstein M, Richenberg Y, Jacobson E, Grossu R, et al. COVID-19 vaccine compliance in adolescents with attention-deficit/hyperactivity disorder. International Journal of Psychiatry in Medicine. 2023;58(2). |
| Shoaib A, Cepeda MS, Murray G, Ochs-Ross R. Autism: Comorbidities and Treatment Patterns in the Real World, a Retrospective Cohort Study Among Children, Adolescents and Adults Newly Diagnosed with Autism. Journal of Autism & Developmental Disorders. 2022;52(10). |
| Slaby I, Hain HS, Abrams D, Mentch FD, Glessner JT, Sleiman PM, et al. An electronic health record (EHR) phenotype algorithm to identify patients with attention deficit hyperactivity disorders (ADHD) and psychiatric comorbidities. Journal of Neurodevelopmental Disorders Vol 14 2022, ArtID 37. 2022;14. |
| Trivedi C, Nandakumar AL, Yousefzadehfard Y, Goriparthi TS, Chaudhari G, Vora D, et al. Suicide risk among adolescents with ADHD: An overview from the National Inpatient Sample data set. Journal of Nervous and Mental Disease. 2023;211(3). |
| Wang SM, Woo YS, Choi WS, Sung HM, Yoon BH, Kwon YJ. Prevalence of psychiatric comorbidities in adult ADHD compared with non-ADHD populations: A systematic review 1.05 Epidemiology. Early Intervention in Psychiatry. 2023;17(Supplement 1). |
| Yamada R, Miyashita K, Hashimoto TM, Hironaka N, Takada K, Shigeta M, et al. Prevalence and Clinical Significance of Psychiatric Comorbidities With Gambling Disorder in 12 Clinical Settings in Japan. Journal of Addiction Medicine. 2023;17(2). |
| Yamauchi Y, Takao S, Matsumoto N, Yorifuji T. Association of nighttime sleep with behaviors in Japanese early childhood. Pediatrics International. 2022;64(1) (no pagination). |
| Yildiz BB, Mutlu C, Ocakoglu FT, Dogan EB, Yalcin O, Dut R, et al. Relationship of Temperament Differences with Diagnosis, Severity and Accompanying Psychiatric Symptoms in Autism Spectrum Disorder. Noropsikiyatri Arsivi. 2022;59(1). |
| Yoshimura A, Matsushita S, Kimura M, Yoneda J-i, Maesato H, Yokoyama A, et al. Influence of ADHD, especially attention-deficit characteristics, on the course of alcohol-dependent individuals. BMC Psychiatry Vol 22 2022, ArtID 803. 2022;22. |
| Young JT, Bellgrove MA, Arunogiri S. Assessment of attention-deficit hyperactivity disorder in people with substance use disorder: Another case of what gets measured gets done. Australian & New Zealand Journal of Psychiatry. 2021;55(8). |
| Yule AM, DiSalvo M, Biederman J, Wilens TE, Dallenbach NT, Taubin D, et al. Decreased risk for substance use disorders in individuals with high-functioning autism spectrum disorder. European Child & Adolescent Psychiatry. 2023;32(2). |
| Zinna S, Luxton R, Papachristou E, Dima D, Kyriakopoulos M. Comorbid chronic tic disorder and Tourette syndrome in children requiring inpatient mental health treatment. Clinical Child Psychology & Psychiatry. 2021;26(3). |
| Zoromski AK, Epstein JN, Ciesielski HA. Unique Associations Between Specific Attention-Deficit Hyperactivity Disorder Symptoms and Related Functional Impairments. Journal of developmental and behavioral pediatrics : JDBP. 2021;42(5). |
|  |
| **From external sources / not from EndNote (n= 18)** |
| Backner W, Clark E, Jenson W, Gardner M, Kahn J. An investigation of psychiatric comorbidity and symptom awareness among male adolescents with autism spectrum disorders. International Journal of School & Educational Psychology. 2013 Oct 2;1(4):259-68. |
| Bertaut S, Rondepierre F, Jalenques I. Evaluation of physical pain in Gilles de la Tourette syndrome, exploratory study. InAnnales Médico-psychologique, psychiatric review 2016 Nov 1 (Vol. 174, No. 9, pp. 800-804). Elsevier Masson. |
| Bron TI, Bijlenga D, Verduijn J, Penninx BW, Beekman AT, Kooij JS. Prevalence of ADHD symptoms across clinical stages of major depressive disorder. Journal of Affective Disorders. 2016 Jun 1;197:29-35. |
| Carroll KM, Rounsaville BJ. History and significance of childhood attention deficit disorder in treatment-seeking cocaine abusers. Comprehensive psychiatry. 1993 Mar 1;34(2):75-82. |
| Chamberlain SR, Derbyshire K, Leppink E, Grant JE. Impact of ADHD symptoms on clinical and cognitive aspects of problem gambling. Comprehensive Psychiatry. 2015 Feb 1;57:51-7. |
| Claudio‐Campos K, Stevens D, Koo SW, Valko A, Bienvenu OJ, Budman CB, Cath DC, Darrow S, Geller D, Goes FS, Grados MA. Is persistent motor or vocal tic disorder a milder form of Tourette syndrome?. Movement Disorders. 2021 Aug;36(8):1899-910. |
| Deberdt, W., Thome, J., Lebrec, J., Kraemer, S., Fregenal, I., Ramos-Quiroga, J. A., & Arif, M. (2015). Prevalence of ADHD in nonpsychotic adult psychiatric care (ADPSYC): A multinational cross-sectional study in Europe. BMC Psychiatry, 15(1), 242 |
| Demb HB, Chang C. The use of psychostimulants in children with disruptive behavior disorders and developmental disabilities in a community setting. Mental Health Aspects of Developmental Disabilities. 2004 Jan;7:26-36. |
| Dew RΕ, Daniel SS, Koenig HG. A pilot study on religiousness/spirituality and ADHD. International journal of adolescent medicine and health. 2007 Oct;19(4):507-10. |
| Eyre SL, Rounsaville BJ, Kleber HD. History of childhood hyperactivity in a clinic population of opiate addicts. The Journal of nervous and mental disease. 1982 Sep 1;170(9):522-9. |
| Kronström, K., Tiiri, E., Vuori, M. et al. Multi-center nationwide study on pediatric psychiatric inpatients 2000–2018: length of stay, recurrent hospitalization, functioning level, suicidality, violence and diagnostic profiles. Eur Child Adolesc Psychiatry 32, 835–846 (2023). https://doi.org/10.1007/s00787-021-01898-0 |
| Lines, K.L. and Sadek, J., 2018. ADHD in acute care psychiatric inpatients. ADHD Attention Deficit and Hyperactivity Disorders, 10, pp.129-133. |
| Nylander, L., Holmqvist, M., Gustafson, L., & Gillberg, C. (2009). ADHD in adult psychiatry. Minimum rates and clinical presentation in general psychiatry outpatients. Nordic Journal of Psychiatry, 63(1), 64–71 |
| Pehlivanidis, A., Papanikolaou, K., Spyropoulou, A.C. and Papadimitriou, G.N., 2014. Comorbid attention-deficit/hyperactivity disorder in adult psychiatric outpatients with depressive or anxiety disorders. International journal of psychiatry in clinical practice, 18(4), pp.265-271. |
| Rao, P., & Place, M. (2011). Prevalence of ADHD in four general adult outpatient clinics in North East England. Progress in Neurology and Psychiatry, 15(5), 7–10 |
| Riedel O, Klau S, Langner I, Bachmann C, Scholle O. Prevalence of multimodal treatment in children and adolescents with ADHD in Germany: a nationwide study based on health insurance data. Child and Adolescent Psychiatry and Mental Health. 2021 Dec;15(1):1-0. |
| Sasaki, H., Jono, T., Fukuhara, R., Honda, K., Ishikawa, T., Boku, S. and Takebayashi, M., 2022. Late-manifestation of attention-deficit/hyperactivity disorder in older adults: an observational study. BMC psychiatry, 22(1), p.354. |
| van de Glind G, Koeter MW, Allsop S, Auriacombe M, Barta C, et al. Psychiatric comorbidity in treatment-seeking substance use disorder patients with and without attention deficit hyperactivity disorder: Results of the IASP study. Addiction. 2014;109(2):262-72. |

**Supplementary Table 2: 210 excluded papers and rationale**

| **Author** | **Year** | **Exclusion rationale** | **Simplified rationale** | **Exclusion code** | **Further information** |
| --- | --- | --- | --- | --- | --- |
| Abiodun | 2011 | primary care - child - phase 1 | Inappropriate sample – primary care | 4.4 |  |
| Adamis | 2022 | Meta-analysis | Inappropriate methodology | 1.2 |  |
| Adamis | 2023 | Uses same population as Adamis 2018 included in phase 1 | Inappropriate design - duplicate sample | 3.13 |  |
| Alpert | 1996 | research group population | research study population | 3.1 |  |
| Anckaraster | 2007 | juvenile setting, legal not medical | not a relevant clinical sample/setting - forensic | 4.3 |  |
| Anholt | 2014 | research study population | Inappropriate design - research study population | 3.1 |  |
| Anitha | 2021 | sample excluded those with pre-existing ADHD diagnosis | Inappropriate design | 3.11 |  |
| Arias | 2008 | childhood ADHD only, not current | lifetime/childhood ADHD only | 3.7 |  |
| Arican | 2019 | systematic review and research study cohort | meta-analysis or systematic review | 1.2 |  |
| Axelson | 2012 | research study population | research study population | 3.1 |  |
| Backner | 2013 | dissertation, not published research | Dissertation only | 1.3 |  |
| Baizabal-Carvallo | 2022 | all ages paper | all ages paper | 4.12 |  |
| Bannett | 2022 | primary care - child - phase 2 | not a relevant clinical sample/setting – Primary Care | 4.4 |  |
| Bartoli | 2023 | systematic review/meta-analysis | meta-analysis or systematic review | 1.2 |  |
| Baruch | 2009 | grouped hyperkinetic & conduct disorder | ADHD prevalence rate not available | 3.14 | author emailed 26.6.23 - replied nil separate data available |
| Bassiony | 2021 | duplicate paper, 2022 version used | duplicate sample | 3.13 |  |
| Bell | 2015 | nil clear data | ADHD prevalence rate not available | 3.14 |  |
| Ben-Yahuda | 2012 | ED setting - phase 1 | not a relevant clinical sample/setting - ED | 4.13 |  |
| Berg | 2023 | poster only | Conference poster presentations | 1.6 |  |
| Bertaut | 2016 | foreign language only (French) | foreign language only | 1.8 |  |
| Beverly | 2021 | ED setting x2 - phase 2 | not a relevant clinical sample/setting - ED | 4.11 |  |
| Beyoglu | 2022 | ED setting x1, one study population with head injury x1 - phase 2 | not a relevant clinical sample/setting - ED | 4.13 |  |
| Biederman | 2004 | prevalence data not provided, rough table only | ADHD prevalence rate not available | 3.14 |  |
| Bitter | 2019 | nil separate country prevalence data available | ADHD prevalence rate not available | 3.14 | author emailed 24.6.23 - nil reply |
| Bjork | 2022 | abstract only | Abstract only texts | 1.5 |  |
| Bonati | 2021 | ascertainment bias as from ADHD centre | ascertainment bias / ADHD samples | 3.8 |  |
| Bozkurt | 2016 | nil prevalence data | ADHD prevalence rate not available | 3.14 |  |
| Bron | 2016 | research study population | research study population | 3.1 |  |
| Carbone | 2022 | proportion of visits, not of the patient population | ADHD prevalence rate not available | 3.9 |  |
| Carlsson | 2013 | criteria "severe hyperactivity or diagnosed ADHD” not specific to ADHD only | ADHD prevalence rate not available | 3.14 |  |
| Carroll | 1993 | looked at childhood ADHD only in adult sample | lifetime/childhood ADHD only | 3.7 |  |
| Carvalho | 2016 | ADHD 'suspected' labelling, inattention sample, lots of int disability dx | inappropriate design +/- sample | 3.8 |  |
| Caye | 2020 | duplication of paper | duplicate sample | 3.13 |  |
| Caye | 2020 | Comparison of representative population with ADHD sample | Inappropriate design - community | 3.3 |  |
| Celluci | 2007 | not a clinical setting – university population | Inappropriate sample – university population | 4.1 |  |
| Cerny | 2023 | sample is referred for "inattention", ascertainment bias likely, to exclude | ascertainment bias / ADHD samples | 3.8 |  |
| Chamberlain | 2015 | population, not clinical sample | Inappropriate design – community study | 3.3 |  |
| Chavira | 2004 | primary care - child - phase 1 | Inappropriate sample – primary care | 4.4 |  |
| Chee | 1994 | all ages paper | Inappropriate sample - all ages paper | 4.12 |  |
| Cheung | 2018 | endocrine specialist clinic | Inappropriate sample – medical clinic | 4.2 |  |
| Claudio Campo | 2021 | international research study population | research study population | 3.1 |  |
| Coffey | 1998 | raw data not available | ADHD prevalence rate not available | 3.14 | author emailed 2/6/23 - nil reply |
| Colgan | 2023 | poster only | Conference poster presentations | 1.6 |  |
| Convertino | 2022 | not clinical population – recruited from schoools | Inappropriate sample – school population | 4.1 |  |
| Conway | 2011 | raw number ADHD not stated | ADHD prevalence rate not available | 3.14 | author emailed 23/6/23 - undeliverable |
| Cook | 2020 | Systematic review | meta-analysis or systematic review | 1.2 |  |
| Cook | 2022 | head injury - child - phase 2 | not a relevant clinical sample/setting | 4.13 |  |
| Cornell | 2022 | Only show the frequency of visits not number of individual patients | ADHD prevalence rate not available | 3.9 |  |
| Cost | 2022 | SDQ only, nil diagnostic/prevalence | Inappropriate design – not diagnostic of ADHD | 2.1 |  |
| Cummings | 2022 | primary care - child - phase 2 | Inappropriate design – general practice / primary care | 4.4 |  |
| Dakwar | 2012 | research study population | research study population | 3.1 |  |
| Danielyan | 2007 | large number referred for ?ADHD, ascertainment bias | ascertainment bias / ADHD samples | 3.8 |  |
| Davids | 2005 | childhood ADHD only | lifetime/childhood ADHD only | 3.7 |  |
| de Vries | 2018 | nil prevalence data available | ADHD prevalence rate not available | 3.14 |  |
| Demb | 2004 | data on stimulant prescribing only, prevalence not available | Data based on prescriptions of stimulants | 3.4 |  |
| Demeter | 2013 | nil data as ADHD is combined with disruptive behaviour disorder | ADHD prevalence rate not available | 3.14 | author emailed 2/6/23 - nil reply |
| Dew | 2007 | Explored link between spirituality and ADHD Dx | Inappropriate design | 3.14 |  |
| Downey | 2018 | ED child - phase 1 | not a relevant clinical sample/setting - ED | 4.11 |  |
| Dy-Hollins | 2023 | poster only | Conference poster presentations | 1.6 |  |
| Eapen | 2004 | primary care - child - phase 1 | not a relevant clinical sample/setting – primary care | 4.4 |  |
| Eddy | 2013 | all ages paper | all ages paper | 4.12 |  |
| Edinoff | 2022 | Narrative review | narrative review | 1.1 |  |
| Eseaton | 2023 | Abstract only | Abstract only texts | 1.5 |  |
| Etain | 2017 | childhood ADHD only, not current | lifetime/childhood ADHD only | 3.7 |  |
| Eyre | 1982 | childhood hyperactivity only, not current ADHD | lifetime/childhood ADHD only | 3.7 |  |
| Fago | 2003 | did not separate ADHD from PDD / other neurodevelopmental disorders | ADHD prevalence rate not available | 3.14 |  |
| Fahrendorff | 2023 | systematic review | meta-analysis or systematic review | 1.2 |  |
| Fischer | 2002 | follow up of a hyperactive group 13 years later, likely ascertainment bias | ascertainment bias / ADHD samples | 3.8 |  |
| Fones | 2000 | research group population | research study population | 3.1 |  |
| Fossati | 2015 | childhood ADHD only | lifetime/childhood ADHD only | 3.7 |  |
| Fossati | 2002 | childhood ADHD only | lifetime/childhood ADHD only | 3.7 |  |
| Frost | 2011 | only partially recruited from clinical settings | Inappropriate design – community samples | 3.3 |  |
| Garakani | 2020 | raw prevalence data not stated | ADHD prevalence rate not available | 3.14 | author emailed 22/6/23 - nil reply |
| Garcia-Delgar | 2022 | research study population | research study population | 3.1 |  |
| Garland | 2001 | too vague a source (San Diego county), inc: juvenile, welfare, school settings | Inappropriate samples | 4.14 |  |
| Gerhand | 2022 | Systematic review | meta-analysis or systematic review | 1.2 |  |
| Ghaziuddin | 1998 | all ages paper | all ages paper | 4.12 |  |
| Gjevik | 2010 | special school population, not clinical | not a relevant clinical sample/setting | 4.1 |  |
| Goldstein | 2004 | already from an ADHD sample, ascertainment bias | ascertainment bias / ADHD samples | 3.8 |  |
| Goossensen | 2006 | nil diagnostic testing for negative screeners, high dropout rate, ?intervention study | ADHD prevalence rate not available | 3.14 |  |
| Gorlin | 2016 | same sample as previous author's study | duplicate sample | 3.13 |  |
| Gould | 2018 | only if ADHD was mild-moderate, ignored severe ADHD | ADHD prevalence rate not available | 3.14 |  |
| Groth | 2017 | same cohort over time | same population over time | 3.6 |  |
| Gyllenberg | 2014 | epidemiological sample | Inappropriate design – epidemiological | 3.3 |  |
| Hansen | 2018 | same sample as previous author's study | duplicate sample | 3.13 |  |
| Hanson | 2013 | research study sample | research study population | 3.1 |  |
| Hardan | 1997 | Sample with mental retardation | Inappropriate sample – intellectual disability | 4.6 |  |
| Haw | 2001 | nil mention of ADHD | ADHD prevalence rate not available | 3.14 |  |
| Henin | 2007 | childhood ADHD only in adult patients | lifetime/childhood ADHD only | 3.7 |  |
| Herd | 2023 | not a clinical population – foster care | Inappropriate sample – foster home | 4.14 |  |
| Hesse | 2010 | nil prevalence data given | ADHD prevalence rate not available | 3.14 |  |
| Hinshaw | 2022 | Discursive paper, no data | ADHD prevalence rate not available | 3.14 |  |
| Hirschtritt | 2015 | Multi-country research study on Tourette’s | ADHD prevalence rate not available | 3.14 |  |
| Holtmann | 2010 | gives proportion of discharges, not prevalence | ADHD prevalence rate not available | 3.14 |  |
| Hong | 2020 | referred for ADHD clinic population | ascertainment bias / ADHD samples | 3.8 |  |
| Hong | 2021 | sample of patients with ADHD already | ascertainment bias / ADHD samples | 3.8 |  |
| Irastorza | 2018 | raw data needed | ADHD prevalence rate not available | 3.14 | author emailed 23/06/23 - no reply |
| Isaac | 1995 | nil ADHD data | ADHD prevalence rate not available | 3.14 |  |
| Jager | 2020 | ENT type sample - not F code sample | not a relevant clinical sample/setting - surgical | 4.2 |  |
| Jainchill | 1997 | nil ADHD data except racial comparison | ADHD prevalence rate not available | 3.14 |  |
| Jaisoorya | 2003 | all ages paper | all ages paper | 4.12 |  |
| Janoczkin | 2021 | not clear if true prevalence vs main diagnosis | ADHD prevalence rate not available | 3.14 | author emailed 23.6.23 - no reply |
| Jerrell | 2008 | nil raw data prevalence ADHD | ADHD prevalence rate not available | 3.14 | author emailed 9.6.23 - domain does not exist |
| Joo | 2012 | childhood ADHD only | lifetime/childhood ADHD only | 3.7 |  |
| Kafka | 1998 | childhood ADHD only | lifetime/childhood ADHD only | 3.7 |  |
| Kambanis | 2020 | research study population | research study population | 3.1 |  |
| Kammer | 2022 | systematic review / meta-analysis | meta-analysis or systematic review | 1.2 |  |
| Karlsdotter | 2016 | 8x multi-country, separate data | ADHD prevalence rate not available | 3.14 | author emailed 23.6.23 - undeliverable |
| Karlsson | 2021 | sub-population of clinic, multicentre, from a drug research study | research study population | 3.1 |  |
| Kaye | 2019 | Multi-country, nil separate data | ADHD prevalence rate not available | 3.14 | author emailed 23.6.23 - replied - same pop as van de Glind 2014 (new paper) |
| Kelly | 2004 | nonclinical sample, inc. detention centres & community, research pool | not a relevant clinical sample/setting | 4.3 |  |
| Kennemer | 2010 | included ADHD NOS | ADHD prevalence rate not available | 3.14 |  |
| Kenneson | 2013 | data from epidemiological survey | Inappropriate design - epidemiological | 3.3 |  |
| Kewitz | 2021 | definitive prevalence not clear | ADHD prevalence rate not available | 3.14 |  |
| Khadr | 2022 | questionnaires to clinicians to obtain patient details | Inappropriate design - surveys | 3.5 |  |
| Kola | 2022 | poster only | Conference poster presentations | 1.6 |  |
| Konrad | 2021 | part community & forensic sample | not a relevant clinical sample/setting | 4.3 |  |
| Koyuncu | 2019 | childhood ADHD only | lifetime/childhood ADHD only | 3.7 |  |
| Koyuncu | 2017 | childhood ADHD only | lifetime/childhood ADHD only | 3.7 |  |
| Koyuncu | 2015 | childhood ADHD only | lifetime/childhood ADHD only | 3.7 |  |
| Kragh | 2019 | multi country, nil separate data | ADHD prevalence rate not available | 3.14 | author emailed 23.6.23 - no reply |
| Kumar | 2011 | seeking kappa coefficient, not clear gold standard | kappa comparison / unclear gold standard | 3.12 |  |
| Kuperman | 2001 | research study population | research study population | 3.1 |  |
| Kutcher | 1989 | nil ADHD prevalence data | ADHD prevalence rate not available | 3.14 |  |
| Lecavalier | 2019 | research study population and hyperactive symptoms only | research study population | 3.1 |  |
| Lee | 2021 | no clear prevalence data | ADHD prevalence rate not available | 3.14 | author emailed for clarification 23.6.23 - undeliverable |
| Lev-Ran | 2012 | insufficient data - cannabis user group only, combined ADHD and disruptive behaviour disorder | ADHD prevalence rate not available | 3.14 | author emailed 26.6.23 - nil reply |
| Lewczyk | 2003 | kappa comparison, vague mental health sample in community & residential home setting | kappa comparison / unclear gold standard | 3.12 |  |
| Lino | 2022 | narrative review | narrative review | 1.1 |  |
| Liu | 2023 | CBCL not diagnostic for ADHD, only behavioural symptoms are described | non-diagnostic instrument | 2.1 |  |
| Lowe | 2019 | change in ADHD prevalence over time in same population | same population over time | 3.6 |  |
| Luk | 2022 | nil prevalence data | ADHD prevalence rate not available | 3.14 |  |
| Luoni | 2018 | combined data for ADHD and conduct disorder | ADHD prevalence rate not available | 3.14 | author emailed 24.6.23 - nil reply |
| Magruder | 2005 | kappa comparison, no gold standard | kappa comparison / unclear gold standard | 3.12 |  |
| Malhotra | 2007 | data combined with conduct disorder, long time-period | ADHD prevalence rate not available | 3.14 |  |
| Mandell | 2007 | Data sourced from insurance claims – not a clinical setting | Inappropriate design – data from insurance claims | 3.15 |  |
| Marr | 2021 | ED child - phase 2 | Inappropriate sample - ED | 4.11 |  |
| Masi | 2008 | same sample as previous author's study | duplicate sample | 3.13 |  |
| Masi | 2007 | same sample as previous author's study | duplicate sample | 3.13 |  |
| Masi | 2007 | same sample as previous author's study | duplicate sample | 3.13 |  |
| Masi | 2006 | same sample as previous author's study | duplicate sample | 3.13 |  |
| Matthews | 2018 | community & learning disability sample | not a relevant clinical sample/setting | 4.6 |  |
| May | 2022 | only examines patients with ADHD, no prevalence | ascertainment bias / ADHD samples | 3.8 |  |
| Mayne | 2016 | primary care - child - phase 1 | not a relevant clinical sample/setting – primary care | 4.4 |  |
| Modestin | 2001 | childhood ADHD only | lifetime/childhood ADHD only | 3.7 |  |
| Moon | 2017 | community population sample | Inappropriate design - community | 3.3 |  |
| Moss | 2020 | only examines patients with ADHD, no prevalence | ascertainment bias / ADHD samples | 3.8 |  |
| Mroczkowski | 2021 | nil prevalence data | ADHD prevalence rate not available | 3.14 |  |
| Mucci | 2022 | three separate samples based on pandemic lockdown timings | ADHD prevalence rate not available | 3.14 |  |
| Nilles | 2022 | journal supplement - abstract only | Abstract only texts | 1.5 |  |
| Nilles | 2023 | journal supplement - abstract only | Abstract only texts | 1.5 |  |
| Olsson | 2022 | used medication prescription as a proxy for ADHD | inappropriate design | 3.4 |  |
| Ordonez | 2016 | research study population | research study population | 3.1 |  |
| Pakyurek | 2022 | ED setting / head trauma sample | inappropriate sample | 4.11 |  |
| Paruk | 2022 | no separate data on inpatient vs outpatient, unclear prevalence data | ADHD prevalence rate not available | 3.14 |  |
| Patel | 2018 | no raw prevalence / gender data - | ADHD prevalence rate not available | 3.14 | author emailed 23.6.23 - no reply |
| Peles | 2012 | childhood ADHD only | lifetime/childhood ADHD only | 3.7 |  |
| Peralta | 2011 | childhood ADHD only | lifetime/childhood ADHD only | 3.7 |  |
| Philipsen | 2008 | data for childhood ADHD and 'severe' adult ADHD only | lifetime/childhood ADHD only | 3.7 |  |
| Ragusa | 2023 | abstract only | Abstract only texts | 1.5 |  |
| Reyes | 2019 | research study population | research study population | 3.1 |  |
| Reyes | 2019 | same sample as previous author's study | duplicate sample | 3.13 |  |
| Rho | 2015 | new childhood ADHD dx only, excluded those w/ childhood ADHD | lifetime/childhood ADHD only | 3.7 |  |
| Richter | 2020 | childhood ADHD only | lifetime/childhood ADHD only | 3.7 |  |
| Riedel | 2021 | not a clinical setting – date from health claims | Inappropriate design – data from insurance claims | 3.15 |  |
| Ross | 2006 | research sample population | research study population | 3.1 |  |
| Rounsaville | 1991 | childhood ADHD only | lifetime/childhood ADHD only | 3.7 |  |
| Rubino | 2009 | childhood ADHD only | lifetime/childhood ADHD only | 3.7 |  |
| Safren | 2001 | childhood ADHD only | lifetime/childhood ADHD only | 3.7 |  |
| Sanchez-Gistau | 2020 | childhood ADHD only | lifetime/childhood ADHD only | 3.7 |  |
| Schiff | 1990 | nil prevalence data | ADHD prevalence rate not available | 3.14 |  |
| Schott | 2022 | Inappropriate sample - ED | Inappropriate sample -ED | 4.11 |  |
| Serra | 2021 | nil raw prevalence data | ADHD prevalence rate not available | 3.14 | author emailed 23.6.23 - undeliverable |
| Sheldrick | 2022 | no raw prevalence data, data looked at total visits, rather than patients | ADHD prevalence rate not available | 3.14 |  |
| Sheppard | 2010 | research study population | research study population | 3.1 |  |
| Shkalim-Zemer | 2023 | Not a clinical sample – data from health fund and prescription records | Inappropriate design – health fund data | 3.15 |  |
| Shoaib | 2022 | Data source from insurance claims, not a clinical setting | Inappropriate design – health insurance claims | 3.15 |  |
| Sivakumar | 2013 | authors screening for ADHD/BPAD symptoms i.e. ascertainment bias | ascertainment bias / ADHD samples | 3.8 |  |
| Slaby | 2022 | combined data for whole hospital from genetic database, rather than separate clinical samples | Inappropriate sample – not a mental health clinical setting | 4.15 |  |
| Speranza | 2011 | research population | research study population | 3.1 |  |
| Srinivasaraghavan | 2013 | suspected ADHD sample, ascertainment bias | ascertainment bias / ADHD samples | 3.8 |  |
| Stadnick | 2017 | Patients from school based mental health program | Inappropriate sample – school population | 4.1 |  |
| Staller | 2005 | same sample as previous author's study | duplicate sample | 3.13 |  |
| Stargatt | 2010 | no specific ADHD prevalence data | ADHD prevalence rate not available | 3.14 |  |
| Stephens | 2014 | research study population | research study population | 3.1 |  |
| Stewart | 1981 | 1979 data, pre-ADHD diagnosis recognition | ADHD-like symptoms but not diagnostic | 2.1 |  |
| Svedlund | 2018 | very similar sample to author's 2017 paper | duplicate sample | 3.13 |  |
| Tang | 2007 | Recruited substance users, not from substance use disorder clinic | Inappropriate design | 3.3 |  |
| Tani | 2006 | childhood ADHD only | lifetime/childhood ADHD only | 3.7 |  |
| Tejeda-Romero | 2018 | all ages paper | all ages paper | 4.12 |  |
| Torok | 2012 | community setting, users of psychostimulants | Inappropriate design - community | 3.3 |  |
| van de Glind | 2013 | nil prevalence data | ADHD prevalence rate not available | 3.14 | author emailed 2.7.23 - replied, see van de glind for results by country |
| Van Emmerik | 2014 | as per van de Glind population sample | duplicate sample | 3.13 |  |
| Vanbronkhorst | 2018 | Sample of kids prescribed antipsychotics, rather than whole clinic | other bias | 3.16 |  |
| Vasa | 2013 | multi country, raw data not stated | ADHD prevalence rate not available | 3.14 | author emailed 29.6.23 - replied data not available |
| Wamithi | 2015 | ED child - phase 1 | Inappropriate sample – emergency department | 4.11 |  |
| Wang | 2023 | poster / abstract | Abstract only texts | 1.5 |  |
| Wapp | 2015 | as per van de Glind population | duplicate sample | 3.13 |  |
| Waseem | 2011 | nil data on ADHD prevalence | ADHD prevalence rate not available | 3.14 | author emailed 23.6.23 - author unable to locate data |
| Weibel | 2018 | ascertainment bias, ADHD diagnosis clinic | ascertainment bias / ADHD samples | 3.8 |  |
| Weintraub | 2020 | research study population | research study population | 3.1 |  |
| Yamauchi | 2022 | Population based survey | Inappropriate design - community | 3.3 |  |
| Yildiz | 2022 | nil ADHD assessment | ADHD prevalence rate not available | 3.14 |  |
| Young | 2021 | Narrative report | Inappropriate methodology - narrative review | 1.1 |  |
| Yule | 2021 | lifetime ADHD not current ADHD | lifetime/childhood ADHD only | 3.7 |  |
| Yule | 2023 | lifetime prevalence of ADHD in an ASD sample, does not specify if current | lifetime/childhood ADHD only | 3.7 |  |
| Ziedonis | 1994 | not current ADHD prevalence | ADHD prevalence rate not available | 3.14 |  |
| Zima | 2016 | not purely psychiatric or paediatric sample | Inappropriate sample - medical | 4.2 |  |
| Zinna | 2021 | duplication of paper | duplicate sample | 3.13 |  |
| Zoromski | 2021 | Data from ADHD web portal software | Inappropriate design - community | 3.3 |  |

**Supplementary Table 3:** **Different nosological definitions of ADHD/HD.**

| **Diagnostic Nosology** | **% of total study populations** |
| --- | --- |
| DSM-III | 2.56 |
| DSM-III-R | 4.27 |
| DSM-IV | 43.9 |
| DSM-IV-TR | 21.1 |
| DSM 5 | 13.4 |
| DSM-III and DSM-IV combined | 0.28 |
| ICD-9 | 1.99 |
| ICD-10 | 6.27 |
| Unstated | 6.27 |

**Supplementary Table 4: Table of changes to protocol as registered in PROSPERO and rationale for change**

| Original protocol | Change | Rationale |
| --- | --- | --- |
| Additional investigators | Named contact (Prof. Wai Chen) and investigator Prof. Jenny Downs are the same, Amy Epstein left the study, and additional investigators include: Dr. Simon Johnson, Dr. Eric Lim, Mr Peter Jacoby, Prof. Stephen V. Faraone, Dr. Benjamin Minche Su, Prof. Marco Solmi, Dr. Benjamin Forrest, Dr. Bethany Furfaro, Dr. Kiri von Klier | Changing commitments of investigators, growth of the team to effectively address all issues. |
| Research question | The research question was to examine prevalence rates of ADHD across different clinical mental health settings. The research question in the paper is to examine the pooled prevalence and specifies additional analysis of modifiers. | We corrected the research question in the manuscript from rates to pooled prevalence. We specified additional detail of analyses of potential modifiers. |
| Participants / population | We explained that groups with intellectual disability were excluded but we provide additional detail on specific neurological or specialist clinics for exclusion, i.e., clinics providing services for individuals with epilepsy, encephalitis, genetic disorders, metabolic disorders and general medical conditions | We excluded these groups because symptoms of ADHD may be observed but the condition is not ADHD and there may be reduced accuracy of diagnosing ADHD. |
| Main outcome | Change from rate of ADHD to prevalence of ADHD. | Correction. |
| Study selection | We have included additional detail of Research Screener, a machine learning-assisted tool that we used for title and abstract screening. | This change represents responsiveness to new technologies where there is evidence to do so. |
| Risk of bias | We evaluated and reported risk of bias for sample size. | Other potential sources of bias listed in the Joanna Briggs Institute Prevalence Critical Appraisal Checklist were included in the inclusion and exclusion criteria for the study (e.g., valid assessment of ADHD), except for sample size, justifying that variable being reported and avoiding duplication of information for the other checklist items. |

**Supplementary Figure 5: Final studies (n=311) included in the systematic review and meta-analysis**

| Abel KF, Ravndal E, Clausen T, Bramness JG. Attention deficit hyperactivity disorder symptoms are common in patients in opioid maintenance treatment. European Addiction Research. 2018;23(6):298-305. |
| --- |
| Adamis D, Graffeo I, Kumar R, Meagher D, O'Neill D, Mulligan O, et al. Screening for attention deficit-hyperactivity disorder (ADHD) symptomatology in adult mental health clinics. Irish Journal of Psychological Medicine. 2018;35(3):193-201. |
| Adler LA, Guida F, Irons S, Rotrosen J, O'Donnell K. Screening and imputed prevalence of ADHD in adult patients with comorbid substance use disorder at a residential treatment facility. Postgraduate Medicine. 2009;121(5):7-10. |
| Albanese MJ, Clodfelter RC, Jr., Pardo TB, Ghaemi S. Underdiagnosis of bipolar disorder in men with substance use disorder. Journal of Psychiatric Practice. 2006;12(2):124-7. |
| Al-Haidar FA. Co-morbidity and treatment of attention deficit hyperactivity disorder in Saudi Arabia. Eastern Mediterranean Health Journal. 2003;9(5-6):988-95. |
| Alikhani R, Tehrani-Doost M, Shahrivar Z. A five-year report on clinical and demographic characteristics of children and adolescents admitted to a major psychiatric hospital. Iranian Journal of Rehabilitation Research. 2019;17(3):253-62. |
| Almeida-Montes. ADHD Prevalence in Adult Outpatients With Nonpsychotic Psychiatric Illnesses. Journal of Attention Disorders. 2007. |
| Al-Sharbati MM, Al-Farsi YM, Al-Sharbati ZM, Al-Sulaimani F, Ouhtit A, Al-Adawi S. Profile of Mental and Behavioral Disorders Among Preschoolers in a Tertiary Care Hospital in Oman: A Retrospective Study. Oman Medical Journal. 2016;31(5):357-64. |
| Al-Sharbati MM, Al-Hussaini AA, Antony SX. Profile of child and adolescent psychiatry in Oman. Saudi Medical Journal. 2003;24(4):391-5. |
| Al-Sharbati MM, Zaidan ZAJ, Dorvlo ASS, Al-Adawi S. Characteristics of ADHD among omani schoolchildren using DSM-IV: Descriptive study. Journal of Attention Disorders. 2011;15(2):139-46. |
| Andersson HW, Lauvsnes ADF, Nordfjaern T. Emerging Adults in Inpatient Substance Use Treatment: A Prospective Cohort Study of Patient Characteristics and Treatment Outcomes. European Addiction Research. 2021;27(3):206-15. |
| Araz Altay M, Bozatli L, Demirci Sipka B, Gorker I. Current Pattern of Psychiatric Comorbidity and Psychotropic Drug Prescription in Child and Adolescent Patients. Medicina. 2019;55(5):17. |
| Arcelus J, Vostanis P. Child psychiatric disorders among primary mental health service attenders. British Journal of General Practice. 2003;53(488):214-6. |
| Aydin S, Crone MR, Siebelink BM, Numans ME, Vermeiren RRJM, Westenberg PM. Informative value of referral letters from general practice for child and adolescent mental healthcare. European Child & Adolescent Psychiatry. 2023;32(2). |
| Badrfam R, Zandifar A, Barkhori Mehni M, Farid M, Rahiminejad F. Comorbidity of adult ADHD and substance use disorder in a sample of inpatients bipolar disorder in Iran. BMC Psychiatry. 2022;22(1). |
| Baeza I, de la Serna E, Amoretti S, Cuesta MJ, Diaz-Caneja CM, Mezquida G, et al. Premorbid Characteristics as Predictors of Early Onset Versus Adult Onset in Patients With a First Episode of Psychosis. Journal of Clinical Psychiatry. 2021;82(6):14. |
| Balazs J, Gyori D, Horvath LO, Meszaros G, Szentivanyi D. Attention-deficit hyperactivity disorder and nonsuicidal self-injury in a clinical sample of adolescents: the role of comorbidities and gender. BMC Psychiatry. 2018;18(1):34. |
| Bassiony MM, Salah El‐Deen GM, Ameen N, Mahdy RS. Prevalence, correlates, and consequences of attention‐deficit/hyperactivity disorder in a clinical sample of adults with tramadol use in Egypt. American Journal on Addictions. 2022;31(1). |
| Basu S, Isaacs AN. Profile of transcultural patients in a regional Child and Adolescent Mental Health Service in Gippsland, Australia: The need for a multidimensional understanding of the complexities. International Journal of Social Psychiatry. 2019;65(3):217-24. |
| Belirgan S, Ersoy MA, Ersoy HT. Prevalence of adult attention deficit hyperactivity disorder and comorbid axis-I disorders among first time applied cases of a general psychiatry outpatient clinic and a private psychotherapy centre. Psychiatry and Clinical Psychopharmacology. 2018;28(1):25-35. |
| Benarous X, Renaud J, Breton JJ, Cohen D, Labelle R, Guile J-M. Are youths with disruptive mood dysregulation disorder different from youths with major depressive disorder or persistent depressive disorder? Journal of Affective Disorders. 2020;265:207-15. |
| Bener A, Dafeeah EE, Abou-Saleh MT, Bhugra D, Ventriglio A. Co-Morbidity between Major Depression and Schizophrenia: Prevalence and Clinical Characteristics. Psychiatria Danubina. 2020;32(1):78-83. |
| Benti M, Bayeta AB, Abu H. Attention Deficit/Hyperactivity Disorder and Associated Factors Among Children Attending Pediatric Outpatient Departments of West Shewa Zone Public Hospitals, Central Ethiopia. Psychology Research & Behavior Management. 2021;14:1077-90. |
| Bergly TH, Somhovd MJ. The relation between ADHD medication and mild cognitive impairment, as assessed by the Montreal Cognitive Assessment (MoCA), in patients entering substance use disorder inpatient treatment. Journal of Dual Diagnosis. 2018;14(4):228-36. |
| Bernardi S, Cortesse S, Solanto M, Hollander E, Pallanti S. Bipolar disorder and comorbid attention deficit hyperactivity disorder. A distinct clinical phenotype? Clinical characteristics and temperamental traits. The World Journal of Biological Psychiatry. 2010;11(3-4):656-66. |
|  |
| Bezborodovs N, Kocane A, Rancans E, Villerusa A. Clinical Utility of the Parent-Report Version of the Strengths and Difficulties Questionnaire (SDQ) in Latvian Child and Adolescent Psychiatry Practice. Medicina. 2022;58(11). |
| Bhat BA, Hussain A, Dar MA, Dar SA, Jabeen N, Rasool S, Shafi S. The Pattern of Psychiatric Morbidity in an Outpatient Child Psychiatry Clinic: A Cross-sectional, Descriptive Study from a Tertiary Care Hospital in Kashmir, North India. Indian Journal of Psychological Medicine. 2018;40(4):349-55. |
| Bhatia MS, Nigam VR, Bohra N, Malik SC. Attention deficit disorder with hyperactivity among paediatric outpatients. Journal of Child Psychology & Psychiatry & Allied Disciplines. 1991;32(2):297-306. |
| Biederman J, Faraone SV, Wozniak J, Mick E, Kwon A, Cayton GA, Clark SV. Clinical correlates of bipolar disorder in a large, referred sample of children and adolescents. Journal of Psychiatric Research. 2005;39(6):611-22. |
| Blanco-Vieira T, Santos M, Ferrao YA, Torres AR, Miguel EC, Bloch MH, et al. The impact of attention deficit hyperactivity disorder in obsessive-compulsive disorder subjects. Depression & Anxiety. 2019;36(6):533-42. |
| Bolstad I, Lien L, Bramness JG. ADHD symptoms as risk factor for PTSD in inpatients treated for alcohol use disorder. Psychiatry Research. 2021;300:113904. |
| Borchardt CM, Bernstein GA. Comorbid disorders in hospitalized bipolar adolescents compared with unipolar depressed adolescents. Child Psychiatry and Human Development. 1995;26(1):11-8. |
| Bosgelmez S, Tufan AE. The prevalence of symptoms and diagnosis of attention-deficit-hyperactivity disorder among adult female patients with mild / moderate major depressive disorder who were referred to the psychiatric outpatient department of a training hospital: A preliminary study. Dusunen Adam. 2014;27(1):27-33. |
| Bozkurt H, Coskun M, Ayaydin H, Adak I, Zoroglu SS. Prevalence and patterns of psychiatric disorders in referred adolescents with Internet addiction. Psychiatry & Clinical Neurosciences. 2013;67(5):352-9. |
| Brandt L, Levin FR, Kraigher D. Impulsive Personality Traits Mediate the Relationship Between Attention-Deficit/Hyperactivity Disorder Symptoms and Psychiatric Comorbidity among Patients with Severe Alcohol Use Disorder. Journal of Dual Diagnosis. 2021;17(3). |
| Bryson SA, Corrigan SK, McDonald TP, Holmes C. Characteristics of children with autism spectrum disorders who received services through community mental health centers. Autism. 2008;12(1):65-82. |
| Budman CL, Bruun RD, Park KS, Olson ME. Rage attacks in children and adolescents with Tourette's disorder: a pilot study. Journal of Clinical Psychiatry. 1998;59(11):576-80. |
| Buica AM, Preda DM, Andrei LE, Stancu M, Gica N, Rad F. Maladaptive Personality Traits in a Group of Patients with Substance Use Disorder and ADHD. Medicina. 2022;58(7). |
| Burket RC, Myers WC. Axis I and personality comorbidity in adolescents with conduct disorder. Bulletin of the American Academy of Psychiatry & the Law. 1995;23(1):73-82. |
| Butler SF, Arredondo DE, McCloskey V. Affective comorbidity in children and adolescents with attention deficit hyperactivity disorder. Annals of Clinical Psychiatry. 1995;7(2):51-5. |
| Carballal Marino M, Gago Ageitos A, Ares Alvarez J, del Rio Garma M, Garcia Cendon C, Goicoechea Castano A, Pena Nieto J. Prevalence of neurodevelopmental, behavioural and learning disorders in Pediatric Primary Care. Anales de Pediatria. 2018;89(3):153-61. |
| Carballo JJ, Rodriguez-Blanco L, Garcia-Nieto R, Baca-Garcia E. Screening for the ADHD Phenotype Using the Strengths and Difficulties Questionnaire in a Clinical Sample of Newly Referred Children and Adolescents. Journal of Attention Disorders. 2018;22(11):1032-9. |
| Carpentier P-J, Knapen LJ, van Gogh MT, Buitelaar JK, De Jong CA. Addiction in developmental perspective: Influence of conduct disorder severity, subtype, and attention-deficit hyperactivity disorder on problem severity and comorbidity in adults with opioid dependence. Journal of Addictive Diseases. 2012;31(1):45-59. |
| Celebi F, Koyuncu A, Ertekin E, Alyanak B, Tukel R. The Features of Comorbidity of Childhood ADHD in Patients With Obsessive Compulsive Disorder. Journal of Attention Disorders. 2020;24(7):973-80. |
| Cengel-Kultur E, Cuhadaroglu-Cetin F, Gokler B. Demographic and clinical features of child abuse and neglect cases. Turkish Journal of Pediatrics. 2007;49(3):256-62. |
| Chiasson J-P, Stavro K, Rizkallah E, Lapierre L, Dussault M, Legault L, Potvin S. Questioning the specificity of ASRS-v1.1 to accurately detect ADHD in substance abusing populations. Journal of Attention Disorders. 2012;16(8):661-3. |
| Chinawa JM, Odetunde OI, Obu HA, Chinawa AT, Bakare MO, Ujunwa FA. Attention deficit hyperactivity disorder: a neglected issue in the developing world. Behavioural Neurology. 2014;2014:694764. |
| Chung T. Does adolescents' readiness to change substance use behavior differ depending on profile of psychiatric comorbidity? Journal of Dual Diagnosis. 2006;2(1):73-88. |
| Cleland C, Magura S, Foote J, Rosenblum A, Kosanke N. Factor structure of the Conners Adult ADHD Rating Scale (CAARS) for substance users. Addictive Behaviors. 2006;31(7):1277-82. |
| Clure C, Brady KT, Saladin ME, Johnson D, Waid R, Rittenbury M. Attention-deficit/hyperactivity disorder and substance use: symptom pattern and drug choice. American Journal of Drug & Alcohol Abuse. 1999;25(3):441-8. |
| Coetzee C, Truter I, Meyer A. Differences in alcohol and cannabis use amongst substance use disorder patients with and without comorbid attention-deficit/hyperactivity disorder. The South African Journal Of Psychiatry : SAJP : The Journal Of The Society Of Psychiatrists Of South Africa. 2022;28. |
| Coetzee C, Truter I, Meyer A. Prevalence and characteristics of South African treatment-seeking patients with substance use disorder and co-occurring attention-deficit/hyperactivity disorder. Expert Review of Clinical Pharmacology. 2020;13(11):1271-80. |
| Comin M, Braquehais MD, Valero S, Bel MJ, Navarro MC, Diaz A, et al. Inpatient physicians and nurses with dual diagnosis: An exploratory study. Journal of Dual Diagnosis. 2014;10(3):156-61. |
| Conejero I, Jaussent I, Lopez R, Guillaume S, Olie E, Hebbache C, et al. Association of symptoms of attention deficit-hyperactivity disorder and impulsive-aggression with severity of suicidal behavior in adult attempters. Scientific Reports. 2019;9(1):4593. |
| Connor DF, Ford JD, Albert DB, Doerfler LA. Conduct disorder subtype and comorbidity. Annals of Clinical Psychiatry. 2007;19(3):161-8. |
| Coppola M, Sacchetto G, Mondola R. Craving for heroin: difference between methadone maintenance therapy patients with and without ADHD. Trends in Psychiatry & Psychotherapy. 2019;41(1):83-6. |
| Corbisiero S, Riecher-Rossler A, Buchli-Kammermann J, Stieglitz R-D. Symptom overlap and screening for symptoms of attention-deficit/hyperactivity disorder and psychosis risk in help-seeking psychiatric patients. Frontiers in Psychiatry Vol 8 2017, ArtID 206. 2017;8. |
| Crowley TJ, Macdonald MJ, Whitmore EA, Mikulich SK. Cannabis dependence, withdrawal, and reinforcing effects among adolescents with conduct symptoms and substance use disorders. Drug & Alcohol Dependence. 1998;50(1):27-37. |
| Crowley TJ, Mikulich SK, Ehlers KM, Whitmore EA, MacDonald MJ. Validity of structured clinical evaluations in adolescents with conduct and substance problems. Journal of the American Academy of Child & Adolescent Psychiatry. 2001;40(3):265-73. |
| Cunha PJ, Goncalves PD, Ometto M, Dos Santos B, Nicastri S, Busatto GF, de Andrade AG. Executive cognitive dysfunction and ADHD in cocaine dependence: searching for a common cognitive endophenotype for addictive disorders. Frontiers in psychiatry Frontiers Research Foundation. 2013;4:126. |
| Daigre C, Grau-Lopez L, Rodriguez-Cintas L, Egido A, Casas M, Roncero C. Administrative prevalence of insomnia and associated clinical features in patients with addiction during active substance use. Actas espanolas de psiquiatria. 2016;44(2):64-71. |
| Daigre C, Grau-Lopez L, Rodriguez-Cintas L, Ros-Cucurull E, Sorribes-Puertas M, Esculies O, et al. The role of dual diagnosis in health-related quality of life among treatment-seeking patients in Spain. Quality of Life Research. 2017;26(12):3201-9. |
| Daigre C, Ramos-Quiroga J, Valero S, Bosch R, Roncero C, Gonzalvo B, et al. Adult ADHD Self-Report Scale (ASRS-v1.1) symptom checklist in patients with substance use disorders. Actas Espanolas de Psiquiatria. 2009;37(6):299-305. |
| Daigre C, Roncero C, Grau-Lopez L, Martinez-Luna N, Prat G, Valero S, et al. Attention deficit hyperactivity disorder in cocaine-dependent adults: a psychiatric comorbidity analysis. American Journal on Addictions. 2013;22(5):466-73. |
| Daigre C, Roncero C, Rodriguez-Cintas L, Ortega L, Lligona A, Fuentes S, et al. Adult ADHD screening in alcohol-dependent patients using the Wender-Utah Rating Scale and the adult ADHD Self-Report Scale. Journal of Attention Disorders. 2015;19(4):328-34. |
| de Bruin EI, Ferdinand RF, Meester S, de Nijs PF, Verheij F. High rates of psychiatric co-morbidity in PDD-NOS. Journal of Autism & Developmental Disorders. 2007;37(5):877-86. |
| de los Cobos JP, Sinol N, Puerta C, Cantillano V, Zurita CL, Trujols J. Features and prevalence of patients with probable adult attention deficit hyperactivity disorder who request treatment for cocaine use disorders. Psychiatry Research. 2011;185(1-2):205-10. |
| de Veld L, van Hoof JJ, Wolberink IM, van der Lely N. The co-occurrence of mental disorders among Dutch adolescents admitted for acute alcohol intoxication. European Journal of Pediatrics. 2021;180(3):937-47. |
| Deas D, Friendly RW, Vo K, Johnson N, Upadhyaya H, Thomas SE. Dual diagnosis and drinking behaviors in an outpatient treatment seeking sample of adolescents with alcohol use disorders. Journal of Dual Diagnosis. 2005;2(1):47-57. |
| Deberdt, W., Thome, J., Lebrec, J., Kraemer, S., Fregenal, I., Ramos-Quiroga, J. A., & Arif, M. (2015). Prevalence of ADHD in nonpsychotic adult psychiatric care (ADPSYC): A multinational cross-sectional study in Europe. BMC Psychiatry, 15(1), 242 |
| Debes NMMM, Lange T, Jessen TL, Hjalgrim H, Skov L. Performance on Wechsler intelligence scales in children with Tourette syndrome. European Journal of Paediatric Neurology. 2011;15(2):146-54. |
| Deepthi K, Sagar Kommu J, Smitha M, Reddy Y. Clinical profile and outcome in a large sample of children and adolescents with obsessive-compulsive disorder: A chart review from a tertiary care center in India. Indian Journal of Psychiatry. 2018;60(2):205-12. |
| DeMilio L. Psychiatric syndromes in adolescent substance abusers. American Journal of Psychiatry. 1989;146(9):1212-4. |
| Di Nicola M, Sala L, Romo L, Catalano V, Even C, Dubertret C, et al. Adult attention-deficit/hyperactivity disorder in major depressed and bipolar subjects: role of personality traits and clinical implications. European Archives of Psychiatry & Clinical Neuroscience. 2014;264(5):391-400. |
| Diaz DR, Landsberger SA, Povlinski J, Sheward J, Sculley C. Psychiatric disorder prevalence among deaf and hard-of-hearing outpatients. Comprehensive Psychiatry. 2013;54(7):991-5. |
| Diaz R, Goti J, Garcia M, Gual A, Serrano L, Gonzalez L, et al. Patterns of substance use in adolescents attending a mental health department. European Child & Adolescent Psychiatry. 2011;20(6):279-89. |
| Doerfler LA, Connor DF, Toscano Jr PF. Aggression, ADHD symptoms, and dysphoria in children and adolescents diagnosed with bipolar disorder and ADHD. Journal of Affective Disorders. 2011;131(1-3):312-9. |
| Downs J, Hotopf M, Ford T, Simonoff E, Jackson R, Shetty H, et al. Clinical predictors of antipsychotic use in children and adolescents with autism spectrum disorders: a historical open cohort study using electronic health records. European Child & Adolescent Psychiatry. 2016;25(6):649-58. |
| Durukan I, Karaman D, Kara K, Turker T, Tufan AE, Yalcin O, Karabekiroglu K. Diagnoses of patients referring to a child and adolescent psychiatry outpatient clinic. Dusunen Adam. 2011;24(2):113-20. |
| El Archi S, Barrault S, Garcia M, Branger S, Maugé D, Ballon N, et al. Adult ADHD Diagnosis, Symptoms of Impulsivity, and Emotional Dysregulation in a Clinical Sample of Outpatients Consulting for a Behavioral Addiction. Journal of Attention Disorders. 2023;27(7). |
| El Ayoubi H, Barrault S, Gateau A, Cortese S, Frammery J, Mollat E, et al. Adult attention-deficit/hyperactivity disorder among alcohol use disorder inpatients is associated with food addiction and binge eating, but not BMI. Appetite. 2021;168:105665. |
| El Ayoubi H, Brunault P, Barrault S, Mauge D, Baudin G, Ballon N, El-Hage W. Posttraumatic Stress Disorder Is Highly Comorbid With Adult ADHD in Alcohol Use Disorder Inpatients. Journal of Attention Disorders. 2021;25(11):1594-602. |
| Endo T, Sugiyama T, Someya T. Attention-deficit/hyperactivity disorder and dissociative disorder among abused children. Psychiatry & Clinical Neurosciences. 2006;60(4):434-8. |
| Ertekin H, Karamustafalıoğlu O, Ertekin Y, Akpınar A, Bakım B, Tankaya O. The comorbidity of adult attention-deficit/hyperactivity disorder in panic disorder patients. Journal of Harran University Medical Faculty. 2013;10(3):108-13. |
| Ertekin H, Karamustafalioglu O, Ertekin YH, Bakim B, Akpinar A. Adult attention deficit and hyperactivity disorder comorbidity in obsessive compulsive disorder. Anatolian Journal of Clinical Investigation. 2015;9(4):167-73. |
| Evren C, Umut G, Bozkurt M, Can Y, Evren B, Agachanli R. Partial mediator role of physical abuse on the relationship between attention-deficit/hyperactivity disorder symptoms and severity of dissociative experiences in a sample of inpatients with alcohol use disorder. Indian Journal of Psychiatry. 2017;59(3):306-12. |
| Evren C, Umut G, Bozkurt M, Teksin-Unal G, Agachanli R, Evren B. Psychometric properties of the Turkish version of the Adult ADHD Self-Report Scale (ASRS-v1.1) in a sample of Inpatients with alcohol use disorder. Dusunen Adam: Journal of Psychiatry and Neurological Sciences. 2016;29(2):109-19. |
| Fabricius V, Langa M, Wilson K. An exploratory investigation of co-occurring substance related and psychiatric disorders. Journal of Substance Use. 2008;13(2):99-114. |
| Falcone T, Mishra L, Carlton E, Lee C, Butler RS, Janigro D, et al. Suicidal behavior in adolescents with first-episode psychosis. Clinical Schizophrenia & Related Psychoses. 2010;4(1):34-40. |
| Faraone SV, Biederman J, Wozniak J, Mundy E, Mennin D, O'Donnell D. Is comorbidity with ADHD a marker for juvenile-onset mania? Journal of the American Academy of Child & Adolescent Psychiatry. 1997;36(8):1046-55. |
| Fatseas M, Hurmic H, Serre F, Debrabant R, Daulouede J-P, Denis C, Auriacombe M. Addiction severity pattern associated with adult and childhood Attention Deficit Hyperactivity Disorder (ADHD) in patients with addictions. Psychiatry Research. 2016;246:656-62. |
| Fava GA. Consultation psychiatry in an Italian Child Guidance Center: A report on 200 referrals. Child Psychiatry and Human Development. 1981;12(2)(5):90-5. |
| Federico A, Mantovani E, Casari R, Bertoldi A, Lugoboni F, Tamburin S. Adult attention-deficit/hyperactivity disorder symptoms, cognitive dysfunction and quality of life in high-dose use of benzodiazepine and Z-drug. Journal of Neural Transmission. 2021;128(7). |
| Feingold D, Nitzan U, Ratzoni G, Lev-Ran S. Clinical Correlates of Alcohol Abuse among Adolescent Psychiatric Inpatients in Israel. Israel Journal of Psychiatry & Related Sciences. 2014;51(4):258-60. |
| Ferre F, Cambra J, Ovejero M, Basurte-Villamor I. Influence of attention deficit hyperactivity disorder symptoms on quality of life and functionality in adults with eating disorders. Actas Espanolas de Psiquiatria. 2017;45(3):98-107. |
| Ferreira-Maia AP, Boronat AC, Boarati MA, Fu-I L, Wang Y-P. Evaluation of bipolar disorder in children and adolescents referred to a mood service: Diagnostic pathways and manic dimensions. Journal of Psychiatric Practice. 2016;22(6):429-41. |
| Ferrer M, Andion O, Matali J, Valero S, Navarro JA, Ramos-Quiroga JA, et al. Comorbid attention-deficit/hyperactivity disorder in borderline patients defines an impulsive subtype of borderline personality disorder. Journal of personality disorders. 2010;24(6):812-22. |
| Ford JD, Spinazzola J, van der Kolk B, Chan G. Toward an empirically based Developmental Trauma Disorder diagnosis and semi-structured interview for children: The DTD field trial replication. Acta Psychiatrica Scandinavica. 2022;145(6). |
| Frazier J, Biederman J, Bellordre C, Garfield S, Geller D, Coffey B, Faraone S. Should the diagnosis of attention-deficit/hyperactivity disorder be considered in children with pervasive developmental disorder? Journal of Attention Disorders. 2001;4(4):203-11. |
| Freeman AJ, Youngstrom EA, Youngstrom JK, Findling RL. Disruptive Mood Dysregulation Disorder in a Community Mental Health Clinic: Prevalence, Comorbidity and Correlates. Journal of Child & Adolescent Psychopharmacology. 2016;26(2):123-30. |
| Gaber TJ, Bouyrakhen S, Herpertz-Dahlmann B, Hagenah U, Holtmann M, Freitag CM, et al. Migration background and juvenile mental health: a descriptive retrospective analysis of diagnostic rates of psychiatric disorders in young people. Glob Health Action. 2013;6:20187. |
| Gadow KD, Devincent CJ, Pomeroy J, Azizian A. Comparison of DSM-IV symptoms in elementary school-age children with PDD versus clinic and community samples. Autism. 2005;9(4):392-415. |
| Gadow KD, Sprafkin J, Nolan EE. DSM-IV Symptoms in community and clinic preschool children. Journal of the American Academy of Child & Adolescent Psychiatry. 2001;40(12):1383-92. |
| Ganesh S, Kandasamy A, Sahayaraj US, Benegal V. Adult Attention Deficit Hyperactivity Disorder in Patients with Substance Use Disorders: A Study from Southern India. Indian Journal of Psychological Medicine. 2017;39(1):59-62. |
| Gao K, Wang Z, Chen J, Kemp DE, Chan PK, Conroy CM, et al. Should an assessment of Axis I comorbidity be included in the initial diagnostic assessment of mood disorders? Role of QIDS-16-SR total score in predicting number of Axis I comorbidity. Journal of Affective Disorders. 2013;148(2-3):256-64. |
| Garcia Marchena N, Araos P, Pavon FJ, Ponce G, Pedraz M, Serrano A, et al. Psychiatric comorbidity and plasma levels of 2-acyl-glycerols in outpatient treatment alcohol users. Analysis of gender differences. Adicciones. 2016;29(2):83-96. |
| Gardvik KS, Rygg M, Torgersen T, Wallander JL, Lydersen S, Indredavik MS. Association of treatment procedures and resilience to symptom load three-years later in a clinical sample of adolescent psychiatric patients. BMC Psychiatry. 2021;21(1). |
| Garralda ME, Bailey D. Psychiatric disorders in general paediatric referrals. Archives of Disease in Childhood. 1989;64(12):1727-33. |
| Gelegen V, Tamam L. Prevalence and clinical correlates of intermittent explosive disorder in Turkish psychiatric outpatients. Comprehensive Psychiatry. 2018;83:64-70. |
| Ghanizadeh A. Association of nail biting and psychiatric disorders in children and their parents in a psychiatrically referred sample of children. Child and Adolescent Psychiatry and Mental Health. 2008;2 (no pagination). |
| Ghanizadeh A. Co-morbidity and factor analysis on attention deficit hyperactivity disorder and autism spectrum disorder DSM-IV-derived items. Journal of Research in Medical Sciences. 2012;17(4):368-72. |
| Gillberg I, Helles A, Billstedt E, Gillberg C. Boys with Asperger syndrome grow up: Psychiatric and neurodevelopmental disorders 20 years after initial diagnosis. Journal of Autism and Developmental Disorders. 2016;46(1):74-82. |
| Girgis J, Martino D, Pringsheim T. Influence of sex on tic severity and psychiatric comorbidity profile in patients with pediatric tic disorder. Developmental Medicine & Child Neurology. 2022;64(4). |
| Gomez R, Vance A, Gomez RM. Validity of the ADHD Bifactor Model in General Community Samples of Adolescents and Adults, and a Clinic-Referred Sample of Children and Adolescents. Journal of Attention Disorders. 2018;22(14):1307-19. |
| Gomez RL, Janowsky D, Zetin M, Huey L, Clopton PL. Adult psychiatric diagnosis and symptoms compatible with the hyperactive child syndrome: a retrospective study. Journal of Clinical Psychiatry. 1981;42(10):389-94. |
| Goodman G, Gerstadt C, Pfeffer CR, Stroh M, Valdez A. ADHD and aggression as correlates of suicidal behavior in assaultive prepubertal psychiatric inpatients. Suicide & Life-Threatening Behavior. 2008;38(1):46-59. |
| Gordon SM, Tulak F, Troncale J. Prevalence and characteristics of adolescents patients with co-occurring ADHD and substance dependence. Journal of Addictive Diseases. 2004;23(4):31-40. |
| Gorlin EI, Dalrymple K, Chelminski I, Zimmerman M. Diagnostic profiles of adult psychiatric outpatients with and without attention deficit hyperactivity disorder. Comprehensive Psychiatry. 2016;70:90-7. |
| Gorman DA, Thompson N, Plessen KJ, Robertson MM, Leckman JF, Peterson BS. Psychosocial outcome and psychiatric comorbidity in older adolescents with Tourette syndrome: controlled study. British Journal of Psychiatry. 2010;197(1):36-44. |
| Grall-Bronnec M, Wainstein L, Augy J, Bouju G, Feuillet F, Venisse J-L, Sebille-Rivain V. Attention deficit hyperactivity disorder among pathological and at-risk gamblers seeking treatment: A hidden disorder. European Addiction Research. 2011;17(5):231-40. |
| Gupta JK, Rohiwal S, Marwah P, Saini A, Maheshwari A, Gupta S, Saini P. The pattern of psychiatric morbidity in an out-patient child psychiatry clinic: A study from a tertiary care centre in Jaipur, North India. Journal of Cardiovascular Disease Research. 2021;12(4):622-7. |
| Gupta PK, Sivakumar T, Agarwal V, Sitholey P. A clinical study of phenomenology and comorbidity of paediatric bipolar disorder. Journal of Indian Association for Child and Adolescent Mental Health. 2012;8(1):12-9. |
| Gupta S, Bhatia G, Sarkar S, Chatterjee B, Balhara YPS, Dhawan A. Adult attention-deficit hyperactivity disorders and its correlates in patients with opioid dependence: An exploratory study. Indian Journal of Psychiatry. 2020;62(5):501-8. |
| Gurkan K, Akcakin M, Kilic BG, Bilgic A. Psychiatric comorbidity and drug treatments in high functioning children and adolescents with Pervasive Developmental Disorders. Neurology Psychiatry and Brain Research. 2008;15(3):143-50. |
| Hallerback MU, Lugnegard T, Gillberg C. ADHD and nicotine use in schizophrenia or Asperger syndrome: a controlled study. Journal of Attention Disorders. 2014;18(5):425-33. |
| Halloran EC, Ross GJ, Carey MP. The relationship of adolescent personality and family environment to psychiatric diagnosis. Child Psychiatry and Human Development. 2002;32(3):201-16. |
| Hanley C, Saleem F, Graffeo I, McCarthy G, Gavin B, McNicholas F, Adamis D. Association of Wender Utah Rating Scale (WURS)-61 items with clinical psychiatric diagnosis in adulthood. Irish Journal of Medical Science. 2021;04:04. |
| Hansen BH, Oerbeck B, Skirbekk B, Kristensen H. Non-obsessive-compulsive anxiety disorders in child and adolescent mental health services -Are they underdiagnosed, and how accurate is referral information? Nordic Journal of Psychiatry. 2016;70(2):133-9. |
| Harada Y, Hayashida A, Hikita S, Imai J, Sasayama D, Masutani S, et al. Impact of behavioral/developmental disorders comorbid with conduct disorder. Psychiatry & Clinical Neurosciences. 2009;63(6):762-8. |
| Harding G, Hu N, Larter N, Montgomery A, Stephensen J, Callaghan L, et al. Health status and health service use of urban Aboriginal children attending an Aboriginal community child health service in Sydney. Journal of Paediatrics and Child Health. 2021;57(7):1072-7. |
| Harmanci H, ÇElİKel FÇ, EtİKan İ. Comorbidity of Adult Attention Deficit and Hyperactivity Disorder in Bipolar and Unipolar Patients. Archives of Neuropsychiatry / Noropsikiatri Arsivi. 2016;53(3):257-62. |
| Hayashida K, Anderson B, Paparella T, Freeman SFN, Forness SR. Comorbid Psychiatric Diagnoses in Preschoolers with Autism Spectrum Disorders. Behavioral Disorders. 2017;35(3):243-54. |
| Heinrichs N, Kamp-Becker I, Bussing R, Schimek M, Becker A, Briegel W. Disruptive behaviors across different disorders: Evaluation of a clinical sample using the eyberg child behavior inventory+. Zeitschrift fur Kinder- und Jugendpsychiatrie und Psychotherapie. 2019;47(1):35-47. |
| High P, Silver EJ, Stein REK, Roizen N, Augustyn M, Blum N. Do Referral Factors Predict a Probable Autism Spectrum Disorder Diagnosis? A DBPNet Study. Academic Pediatrics. 2022;22(2). |
| Hofvander B, Delorme R, Chaste P, Nyden A, Wentz E, Stahlberg O, et al. Psychiatric and psychosocial problems in adults with normal-intelligence autism spectrum disorders. BMC Psychiatry. 2009;9:35. |
| Huntley Z, Maltezos S, Williams C, Morinan A, Hammon A, Ball D, et al. Rates of undiagnosed attention deficit hyperactivity disorder in London drug and alcohol detoxification units. BMC Psychiatry Vol 12 2012, ArtID 223. 2012;12. |
| Ishizuka K, Ishiguro T, Nomura N, Inada T. Depressive mood changes are associated with self-perceptions of ADHD characteristics in adults. Psychiatry Research. 2021;300:113893. |
| Ivanenko A, Crabtree VM, O'Brien LM, Gozal D. Sleep complaints and psychiatric symptoms in children evaluated at a pediatric mental health clinic. Journal of Clinical Sleep Medicine. 2006;2(1):42-8. |
| Jafferany M, Osuagwu FC, Khalid Z, Oberbarnscheidt T, Roy N. Prevalence and clinical characteristics of body dysmorphic disorder in adolescent inpatient psychiatric patients-a pilot study. Nordic Journal of Psychiatry. 2019;73(4-5):244-7. |
| Jaideep T, Reddy Y, Srinath S. Comorbidity of attention deficit hyperactivity disorder in juvenile bipolar disorder. Bipolar Disorders. 2006;8(2):182-7. |
| Jankovic J, Gelineau-Kattner R, Davidson A. Tourette's syndrome in adults. Movement Disorders. 2010;25(13):2171-5. |
| Jarbin H, Höglund K, Skarphedinsson G, Bremander A. Aerobic exercise for adolescent outpatients with persistent major depression: Feasibility and acceptability of moderate to vigorous group exercise in a clinically referred sample. Clinical Child Psychology & Psychiatry. 2021;26(4). |
| Jaworowski S, Mergui J, Golmard J-L, Fine N, Alfassi T, Lavy DG, et al. Prevalence of Attention Deficit Hyperactivity Disorder Among 255 Patients in Three Israeli Methadone Clinics62. Israel Journal of Psychiatry & Related Sciences. 2020;57(1):55-62. |
| Jayaprakash R, Rajamohanan K, Anil P. Determinants of symptom profile and severity of conduct disorder in a tertiary level pediatric care set up: A pilot study. Indian Journal of Psychiatry. 2014;56(4):330-6. |
| Jerrell JM, McIntyre RS, Deroche CB. Diagnostic clusters associated with an early onset schizophrenia diagnosis among children and adolescents. Human Psychopharmacology. 2017;32(2):03. |
| Jerrell JM, Shugart MA. Community-based care for youths with early and very-early onset bipolar I disorder. Bipolar Disorders. 2004;6(4):299-304. |
| Jiang L, Li Y, Zhang X, Jiang W, Yang C, Hao N, et al. Twelve-year retrospective analysis of outpatients with Attention-Deficit/Hyperactivity Disorder in Shanghai. Shanghai Jingshen Yixue. 2013;25(4):236-42. |
| Johnston K, Dittner A, Bramham J, Murphy C, Knight A, Russell A. Attention deficit hyperactivity disorder symptoms in adults with autism spectrum disorders. Autism research : Official Journal of the International Society for Autism Research. 2013;6(4):225-36. |
| Kafka MP, Hennen J. A DSM-IV Axis I comorbidity study of males (n = 120) with paraphilias and paraphilia-related disorders. Sexual Abuse: Journal of Research & Treatment. 2002;14(4):349-66. |
| Kamal RM, Dijkstra BAG, de Weert-van Oene GH, van Duren JAM, de Jong CAJ. Psychiatric comorbidity, psychological distress, and quality of life in gamma-hydroxybutyrate-dependent patients. Journal of Addictive Diseases. 2017;36(1):72-9. |
| Kantzer A-K, Fernell E, Westerlund J, Hagberg B, Gillberg C, Miniscalco C. Young children who screen positive for autism: Stability, change and "comorbidity" over two years. Research in Developmental Disabilities. 2018;72:297-307. |
| Karaahmet E, Konuk N, Dalkilic A, Saracli O, Atasoy N, Kurcer MA, Atik L. The comorbidity of adult attention-deficit/hyperactivity disorder in bipolar disorder patients. Comprehensive Psychiatry. 2013;54(5):549-55. |
| Kast KA, Rao V, Wilens TE. Pharmacotherapy for Attention-Deficit/Hyperactivity Disorder and Retention in Outpatient Substance Use Disorder Treatment: A Retrospective Cohort Study. Journal of Clinical Psychiatry. 2021;82(2):23. |
| Kawabe K, Horiuchi F, Miyama T, Jogamoto T, Aibara K, Ishii E, Ueno S-i. Internet addiction and attention-deficit / hyperactivity disorder symptoms in adolescents with autism spectrum disorder. Research in Developmental Disabilities. 2019;89:22-8. |
| Kawata T, Sugihara G, Kakibuchi Y, Tomitaka M, Miyajima M, Matsushima E, et al. Attention-deficit hyperactivity symptoms and risk of alcohol use relapse. Neuropsychopharmacology Reports. 2023;43(1). |
| Keller KM, Fox RA. Toddlers with developmental delays and challenging behaviors. Early Child Development and Care. 2009;179(1):87-92. |
| Kessing LV, Vradi E, Andersen PK. Diagnostic stability in pediatric bipolar disorder. Journal of Affective Disorders. 2015;172:417-21. |
| Khairkar P, Pathak C, Lakhkar B, Sarode R, Vagha J, Jagzape T, et al. A 5-year hospital prevalence of child and adolescent psychiatric disorders from central India. Indian Journal of Pediatrics. 2013;80(10):826-31. |
| Kim J-W, Park C-S, Hwang J-W, Shin M-S, Hong K-E, Cho S-C, Kim B-N. Clinical and genetic characteristics of Korean male alcoholics with and without attention deficit hyperactivity disorder. Alcohol and Alcoholism. 2006;41(4):407-11. |
| King VL, Brooner RK, Kidorf MS, Stoller KB, Mirsky AF. Attention deficit hyperactivity disorder and treatment outcome in opioid abusers entering treatment. Journal of Nervous & Mental Disease. 1999;187(8):487-95. |
| Kommu JVS, Gayathri K, Srinath S, Girimaji SC, Seshadri SP, Gopalakrishna G, Subbakrishna DK. Profile of two hundred children with Autism Spectrum Disorder from a tertiary child and adolescent psychiatry centre. Asian Journal of Psychiatry. 2017;28:51-6. |
| Kopp S, Gillberg C. Swedish child and adolescent psychiatric out-patients: A five-year cohort. European Child and Adolescent Psychiatry. 2003;12(1):30-5. |
| Korsgaard HO, Torgersen S, Wentzel-Larsen T, Ulberg R. Substance abuse and personality disorder comorbidity in adolescent outpatients: Are girls more severely ill than boys? Child and Adolescent Psychiatry and Mental Health Vol 10 2016, ArtID 8. 2016;10. |
| Kousha M, Mehdizadeh Tehrani S. Normative life events and PTSD in children: how easy stress can affect children's brain. Acta Medica Iranica. 2013;51(1):47-51. |
| Kronstrom K, Ellila H, Kuosmanen L, Kaljonen A, Sourander A. Changes in the clinical features of child and adolescent psychiatric inpatients: a nationwide time-trend study from Finland. Nordic Journal of Psychiatry. 2016;70(6):436-41. |
| Kronström, K., Tiiri, E., Vuori, M. et al. Multi-center nationwide study on pediatric psychiatric inpatients 2000–2018: length of stay, recurrent hospitalization, functioning level, suicidality, violence and diagnostic profiles. Eur Child Adolesc Psychiatry 32, 835–846 (2023). https://doi.org/10.1007/s00787-021-01898-0 |
| Kulacaoglu F, Solmaz M, Belli H, Ardic FC, Akin E, Kose S. The relationship between impulsivity and attention-deficit/hyperactivity symptoms in female patients with borderline personality disorder. Psychiatry and Clinical Psychopharmacology. 2017;27(3):255-61. |
| Kumar SV, Chate SS, Patil NM, Tekalaki BV, Patil S. Prevalence of undiagnosed and untreated attention deficit hyperactivity disorder in men with alcohol dependence: A case-control study. Archives of Psychiatry and Psychotherapy. 2018;20(1):26-32. |
| Kusaka H, Miyawaki D, Nakai Y, Okamoto H, Futoo E, Goto A, et al. Psychiatric comorbidity in children with high-functioning pervasive developmental disorder. Osaka City Medical Journal. 2014;60(1):1-10. |
| Lamanna AL, Craig F, Matera E, Simone M, Buttiglione M, Margari L. Risk factors for the existence of attention deficit hyperactivity disorder symptoms in children with autism spectrum disorders. Neuropsychiatric Disease & Treatment. 2017;13:1559-67. |
| Lazaro L, Castro-Fornieles J, de la Fuente JE, Baeza I, Morer A, Pamias M. Differences between prepubertal- versus adolescent- onset bipolar disorder in a Spanish clinical sample. European Child & Adolescent Psychiatry. 2007;16(8):510-6. |
| Leader G, Moore R, Chen JL, Caher A, Arndt S, Maher L, et al. Attention deficit hyperactivity disorder (ADHD) symptoms, comorbid psychopathology, behaviour problems and gastrointestinal symptoms in children and adolescents with autism spectrum disorder. Irish Journal of Psychological Medicine. 2021:1-11. |
| Lee DO, Ousley OY. Attention-deficit hyperactivity disorder symptoms in a clinic sample of children and adolescents with pervasive developmental disorders. Journal of Child & Adolescent Psychopharmacology. 2006;16(6):737-46. |
| Leopold K, Ratzer S, Correll CU, Rottmann-Wolf M, Pfeiffer S, Ritter P, et al. Characteristics, symptomatology and naturalistic treatment in individuals at-risk for bipolar disorders: baseline results in the first 180 help-seeking individuals assessed at the Dresden high-risk project. Journal of Affective Disorders. 2014;152-154:427-33. |
| Leung V, Chan L. A cross-sectional cohort study of prevalence, co-morbidities, and correlates of attention-deficit hyperactivity disorder among adult patients admitted to the Li Ka Shing psychiatric outpatient clinic, Hong Kong. East Asian Archives of Psychiatry. 2017;27(2):63-70. |
| Levin FR, Evans SM, Kleber HD. Prevalence of adult attention-deficit hyperactivity disorder among cocaine abusers seeking treatment. Drug & Alcohol Dependence. 1998;52(1):15-25. |
| Lewin AB, Chang S, McCracken J, McQueen M, Piacentini J. Comparison of clinical features among youth with tic disorders, obsessive-compulsive disorder (OCD), and both conditions. Psychiatry Research. 2010;178(2):317-22. |
| Lines, K.L. and Sadek, J., 2018. ADHD in acute care psychiatric inpatients. ADHD Attention Deficit and Hyperactivity Disorders, 10, pp.129-133. |
| Lohit SR, Babu GN, Sharma S, Rao S, Sachin BS, Matkar AV. Prevalence of Adult ADHD Co-morbidity in Alcohol Use Disorders in a General Hospital Setup. Indian Journal of Psychological Medicine. 2019;41(6):523-8. |
| Luderer M, Kaplan-Wickel N, Richter A, Reinhard I, Kiefer F, Weber T. Screening for adult attention-deficit/hyperactivity disorder in alcohol dependent patients: Underreporting of ADHD symptoms in self-report scales. Drug & Alcohol Dependence. 2019;195:52-8. |
| Lugoboni F, Levin FR, Pieri MC, Manfredini M, Zamboni L, Somaini L, Gerra. Co-occurring Attention Deficit Hyperactivity Disorder symptoms in adults affected by heroin dependence: Patients characteristics and treatment needs. Psychiatry Research. 2017;250:210-6. |
| Mancini C, Van Ameringen M, Oakman JM, Figueiredo D. Childhood attention deficit/hyperactivity disorder in adults with anxiety disorders. Psychological Medicine. 1999;29(3):515-25. |
| Manohar H, Kuppili PP, Kandasamy P, Chandrasekaran V, Rajkumar RP. Implications of comorbid ADHD in ASD interventions and outcome: Results from a naturalistic follow up study from south India. Asian Journal of Psychiatry. 2018;33:68-73. |
| Manor I, Gutnik I, Ben-Dor D, Apter A, Sever J, Tyano S, et al. Possible association between attention deficit hyperactivity disorder and attempted suicide in adolescents-A pilot study. European Psychiatry. 2010;25(3):146-50. |
| Marin A, Scott D, Groll DL. Bipolar disorder comorbid with attention-deficit/hyperactivity disorder in adult inpatients with acute relapse. The Primary Care Companion to CNS Disorders. 2013;15(4). |
| Marks S, Shaikh U, Hilty DM, Cole S. Weight status of children and adolescents in a telepsychiatry clinic. Telemedicine Journal & E-Health. 2009;15(10):970-4. |
| Martins J, Roberts N, Nesdole R, Reddy PS, Groll D. Attention deficit hyperactivity disorder presentations to thchild and adolescent mental health urgent consult clinic. Journal of the Canadian Academy of Child and Adolescent Psychiatry. 2019;28(2):66-71. |
| Marwitz L, Pringsheim T. Clinical Utility of Screening for Anxiety and Depression in Children with Tourette Syndrome. Journal of the Canadian Academy of Child & Adolescent Psychiatry = Journal de l’Academie canadienne de psychiatrie de l’enfant et de l’adolescent. 2018;27(1):15-21. |
| Masi G, Berloffa S, Muratori P, Mucci M, Viglione V, Villafranca A, et al. A Naturalistic Study of Youth Referred to a Tertiary Care Facility for Acute Hypomanic or Manic Episode. Brain Sciences. 2020;10(10):29. |
| Masi G, Milone A, Manfredi A, Pari C, Paziente A, Millepiedi S. Conduct disorder in referred children and adolescents: clinical and therapeutic issues. Comprehensive Psychiatry. 2008;49(2):146-53. |
| Masi G, Perugi G, Toni C, Millepiedi S, Mucci M, Bertini N, Akiskal HS. Obsessive-compulsive bipolar comorbidity: focus on children and adolescents. Journal of Affective Disorders. 2004;78(3):175-83. |
| Masi G, Perugi G, Toni C, Millepiedi S, Mucci M, Bertini N, Akiskal HS. Predictors of treatment nonresponse in bipolar children and adolescents with manic or mixed episodes. Journal of Child & Adolescent Psychopharmacology. 2004;14(3):395-404. |
| Masi G, Perugi G, Toni C, Millepiedi S, Mucci M, Bertini N, Pfanner C. Attention-deficit hyperactivity disorder - Bipolar comorbidity in children and adolescents. Bipolar Disorders. 2006;8(4):373-81. |
| McPhate L, Williams K, Vance A, Winther J, Pang K, May T. Gender Variance in Children and Adolescents with Neurodevelopmental and Psychiatric Conditions from Australia. Archives of Sexual Behavior. 2021;50(3):863-71. |
| Mihan R, Shahrivar Z, Mahmoudi-Gharaei J, Shakiba A, Hosseini M. Attention-Deficit Hyperactivity Disorder in Adults Using Methamphetamine: Does It Affect Comorbidity, Quality of Life, and Global Functioning? Iranian Journal of Psychiatry. 2018;13(2):111-8. |
| Milin R, Loh E, Chow J, Wilson A. Assessment of symptoms of attention-deficit hyperactivity disorder in adults with substance use disorders. Psychiatric Services. 1997;48(11):1378-80, 95. |
| Miovsky M, Lukavska K, Rubasova E, Stastna L, Sefranek M, Gabrhelik R. Attention Deficit Hyperactivity Disorder among Clients Diagnosed with a Substance Use Disorder in the Therapeutic Communities: Prevalence and Psychiatric Comorbidity. European Addiction Research. 2021;27(2):87-96. |
| Moodley SV, Pillay AL. Two years of admissions to Natal's first inpatient child mental health centre. South African Medical Journal Suid-Afrikaanse Tydskrif Vir Geneeskunde. 1993;83(3):209-11. |
| Mukaddes NM, Hergner S, Tanidir C. Psychiatric disorders in individuals with high-functioning autism and Asperger's disorder: Similarities and differences. World Journal of Biological Psychiatry. 2010;11(8):964-71. |
| Mukaddes NM, Mutluer T, Ayik B, Umut A. What happens to children who move off the autism spectrum? Clinical follow-up study. Pediatrics International. 2017;59(4):416-21. |
| Myers WC, Burket RC, Otto TA. Conduct disorder and personality disorders in hospitalized adolescents. Journal of Clinical Psychiatry. 1993;54(1):21-6. |
| Nasser EH, Overholser JC. Assessing varying degrees of lethality in depressed adolescent suicide attempters. Acta Psychiatrica Scandinavica. 1999;99(6):423-31. |
| Noterdaeme M, Amorosa H. Evaluation of emotional and behavioral problems in language impaired children using the Child Behavior Checklist. European Child & Adolescent Psychiatry. 1999;8(2):71-7. |
| Nylander, L., Holmqvist, M., Gustafson, L., & Gillberg, C. (2009). ADHD in adult psychiatry. Minimum rates and clinical presentation in general psychiatry outpatients. Nordic Journal of Psychiatry, 63(1), 64–71 |
| Ohlmeier MD, Peters K, Wildt BT, Zedler M, Ziegenbein M, Wiese B, et al. Comorbidity of alcohol and substance dependence with attention-deficit/hyperactivity disorder (ADHD). Alcohol and Alcoholism. 2008;43(3):300-4. |
| Olashore AA, Frank-Hatitchki B, Ogunwobi O. Diagnostic profiles and predictors of treatment outcome among children and adolescents attending a national psychiatric hospital in Botswana. Child & Adolescent Psychiatry & Mental Health [Electronic Resource]. 2017;11:8. |
| Ooi YP, Tan ZJ, Lim CX, Goh TJ, Sung M. Prevalence of behavioural and emotional problems in children with high-functioning autism spectrum disorders. Australian & New Zealand Journal of Psychiatry. 2011;45(5):370-5. |
| Pachado MP, Scherer JN, Guimaraes LSP, von Diemen L, Pechansky F, Kessler FHP, de Almeida RMM. Markers for Severity of Problems in Interpersonal Relationships of Crack Cocaine Users from a Brazilian Multicenter Study. Psychiatric Quarterly. 2018;89(4):923-36. |
| Pehlivanidis, A., Papanikolaou, K., Spyropoulou, A.C. and Papadimitriou, G.N., 2014. Comorbid attention-deficit/hyperactivity disorder in adult psychiatric outpatients with depressive or anxiety disorders. International journal of psychiatry in clinical practice, 18(4), pp.265-271. |
| Perugi G, Ceraudo G, Vannucchi G, Rizzato S, Toni C, Dell'Osso L. Attention deficit/hyperactivity disorder symptoms in Italian bipolar adult patients: a preliminary report. Journal of Affective Disorders. 2013;149(1-3):430-4. |
| Pianca TG, Rohde LA, Rosa RL, Begnis APA, Ferronatto PB, Jensen MC, et al. Crack cocaine use in adolescents: Clinical characteristics and predictors of early initiation. Journal of Clinical Psychiatry. 2016;77(10):e1205-e10. |
| Pinna M, Visioli C, Rago CM, Manchia M, Tondo L, Baldessarini RJ. Attention deficit-hyperactivity disorder in adult bipolar disorder patients. Journal of Affective Disorders. 2019;243:391-6. |
| Pringsheim T, Hammer T. Social behavior and comorbidity in children with tics. Pediatric Neurology. 2013;49(6):406-10. |
| Prochaska JJ, Fromont SC, Delucchi K, Young-Wolff KC, Benowitz NL, Hall S, et al. Multiple risk-behavior profiles of smokers with serious mental illness and motivation for change. Health Psychology. 2014;33(12):1518-29. |
| Rad F, Buica A, Stancu M, Irimie-Ana A, Andrei E, Rosca D, Dobrescu I. Adult ADHD symptoms in a group of patients with substance abuse. Rivista di Psichiatria. 2020;55(3):161-7. |
| Ralph N, McMenamy C. Treatment outcomes in an adolescent chemical dependency program. Adolescence. 1996;31(121):91-107. |
| Raman N, Janse van Rensburg AB. Clinical and psycho-social profile of child and adolescent mental health care users and services at an urban child mental health clinic in South Africa. African Journal of Psychiatry. 2013;16(5):356-63. |
| Rao, P., & Place, M. (2011). Prevalence of ADHD in four general adult outpatient clinics in North East England. Progress in Neurology and Psychiatry, 15(5), 7–10 |
| Rawat VS, Dhiman V, Sinha S, Sagar KJV, Thippeswamy H, Chaturvedi SK, et al. Co-morbidities and outcome of childhood psychogenic non-epileptic seizures-An observational study. Seizure. 2015;25:95-8. |
| Regan T, Tubman J. Attention Deficit Hyperactivity Disorder (ADHD) Subtypes, Co-Occurring Psychiatric Symptoms and Sexual Risk Behaviors among Adolescents Receiving Substance Abuse Treatment. Substance Use & Misuse. 2020;55(1):119-32. |
| Retz W, Ringling J, Retz-Junginger P, Vogelgesang M, Rosler M. Association of attention-deficit/hyperactivity disorder with gambling disorder. Journal of Neural Transmission. 2016;123(8):1013-9. |
| Rey JM, Morris-Yates A, Singh M, Andrews G, Stewart GW. Continuities between psychiatric disorders in adolescents and personality disorders in young adults. American Journal of Psychiatry. 1995;152(6):895-900. |
| Rey JM. Comorbidity between disruptive disorders and depression in referred adolescents. Australian & New Zealand Journal of Psychiatry. 1994;28(1):106-13. |
| Ribeiro SN, Jennen-Steinmetz C, Schmidt MH, Becker K. Nicotine and alcohol use in adolescent psychiatric inpatients: associations with diagnoses, psychosocial factors, gender and age. Nordic Journal of Psychiatry. 2008;62(4):315-21. |
| Rimal HS, Pokharel A. Prevalence of attention deficit hyperactivity disorder among school children and associated co-morbidities - a hospital based descriptive study. Kathmandu University Medical Journal. 2016;14(55):226-30. |
| Roberts N, Booij L, Axas N, Repetti L. Two-year prospective study of characteristics and outcome of adolescents referred to an adolescent urgent psychiatric clinic. International Journal of Adolescent Medicine and Health. 2018;30(1):1-5. |
| Roest S, Siebelink B, van Ewijk H, Vermeiren R, Middeldorp C, van der Lans R. Sociodemographic and clinical characteristics in child and youth mental health; comparison of routine outcome measurements of an Australian and Dutch outpatient cohort. Epidemiology and Psychiatric Sciences Vol 30 2021, ArtID e74. 2021;30. |
| Roncero C, Ortega L, Perez-Pazos J, Lligona A, Abad AC, Gual A, et al. Psychiatric Comorbidity in Treatment-Seeking Alcohol Dependence Patients With and Without ADHD. Journal of Attention Disorders. 2019;23(12):1497-504. |
| Ruiz-Ramos D, Martinez-Magana JJ, Garcia AR, Juarez-Rojop IE, Gonzalez-Castro TB, Tovilla-Zarate CA, et al. Psychiatric Comorbidity in Mexican Adolescents with a Diagnosis of Eating Disorders Its Relationship with the Body Mass Index. International Journal of Environmental Research & Public Health [Electronic Resource]. 2021;18(8):08. |
| Ryden E, Thase M, Straht D, Aberg-Wistedt A, Bejerot S, Landen M. A history of childhood attention-deficit hyperactivity disorder (ADHD) impacts clinical outcome in adult bipolar patients regardless of current ADHD. Acta Psychiatrica Scandinavica. 2009;120(3):239-46. |
| Saba L, Byrne A, Mulligan A. Child art psychotherapy in CAMHS: Which cases are referred and which cases drop out? Springerplus. 2016;5(1):1816. |
| Sadeghian Nadooshan MR, Shahrivar Z, Mahmoudi Gharaie J, Salehi L. ADHD in adults with major depressive or bipolar disorder: does it affect clinical features, comorbidity, quality of life, and global functioning? BMC Psychiatry. 2022;22(1). |
| Sagar R, Pattanayak RD, Mehta M. Clinical profile of mood disorders in children. Indian Pediatrics. 2012;49(1):21-3. |
| Sala L, Martinotti G, Carenti M, Romo L, Oumaya M, Pham-Scottez A, et al. Attention-deficit/hyperactivity disorder symptoms and psychological comorbidity in eating disorder patients. Eating and Weight Disorders. 2018;23(4):513-9. |
| Salous A, Al-Alem L, Omar HA. Trends in mental health of an adolescent medicine clinic patient population. International Journal of Adolescent Medicine & Health. 2009;21(1):9-14. |
| Samar SM, Moyano MB, Brana-Berrios M, Irazoqui G, Matos A, Kichic R, et al. Children and adolescents with Tourette's disorder in the USA versus Argentina: behavioral differences may reflect cultural factors. European Child & Adolescent Psychiatry. 2013;22(11):701-7. |
| Sasaki, H., Jono, T., Fukuhara, R., Honda, K., Ishikawa, T., Boku, S. and Takebayashi, M., 2022. Late-manifestation of attention-deficit/hyperactivity disorder in older adults: an observational study. BMC psychiatry, 22(1), p.354. |
| Sathyabama R. Clinical characteristics and demographic profile of children with Autism Spectrum Disorder (ASD) at child development clinic (CDC), Penang Hospital, Malaysia. Medical Journal of Malaysia. 2019;74(5):372-6. |
| Say GN, Tasdemir HA, Akbas S, Yuce M, Karabekiroglu K. Self-esteem and psychiatric features of Turkish adolescents with psychogenic non-epileptic seizures: a comparative study with epilepsy and healthy control groups. International Journal of Psychiatry in Medicine. 2014;47(1):41-53. |
| Say GN, Tasdemir HA, Ince H. Semiological and psychiatric characteristics of children with psychogenic nonepileptic seizures: Gender-related differences. Seizure. 2015;31:144-8. |
| Schachar RJ, Dupuis A, Anagnostou E, Georgiades S, Soreni N, Arnold PD, et al. Obsessive-compulsive disorder in children and youth: neurocognitive function in clinic and community samples. Journal of Child Psychology & Psychiatry & Allied Disciplines. 2022;63(8). |
| Schubiner H, Tzelepis A, Milberger S, Lockhart N, Kruger M, Kelley BJ, Schoener EP. Prevalence of attention-deficit/hyperactivity disorder and conduct disorder among substance abusers. Journal of Clinical Psychiatry. 2000;61(4):244-51. |
| Schulte-Ruther M, Kulvicius T, Stroth S, Wolff N, Roessner V, Marschik PB, et al. Using machine learning to improve diagnostic assessment of ASD in the light of specific differential and co-occurring diagnoses. Journal of Child Psychology & Psychiatry & Allied Disciplines. 2023;64(1). |
| Seitz J, Kahraman-Lanzerath B, Legenbauer T, Sarrar L, Herpertz S, Salbach-Andrae H, et al. The role of impulsivity, inattention and comorbid ADHD in patients with bulimia nervosa. PLoS ONE [Electronic Resource]. 2013;8(5):e63891. |
| Seleem MA, Amer RA, Romeh AH, Hamoda HM. Demographic and clinical characteristics of children seeking psychiatric services in the Nile Delta region: an observational retrospective study. International Journal of Mental Health Systems. 2019;13:66. |
| Seleem MA, Amer RA. Demographic & clinical correlates of admission into a specialized psychiatric inpatient service for children and adolescents in Egypt: An observational retrospective study. Journal of Psychiatric & Mental Health Nursing (John Wiley & Sons, Inc). 2021;28(6). |
| Selles RR, Storch EA, Lewin AB. Variations in symptom prevalence and clinical correlates in younger versus older youth with obsessive-compulsive disorder. Child Psychiatry & Human Development. 2014;45(6):666-74. |
| Sentissi O, Navarro JC, Oliveira HD, Gourion D, Bourdel MC, Bayle FJ, et al. Bipolar disorders and quality of life: The impact of attention deficit/hyperactivity disorder and substance abuse in euthymic patients. Psychiatry Research. 2008;161(1):36-42. |
| Shekunov J, Wozniak J, Conroy K, Pinsky E, Fitzgerald M, De Leon MF, et al. Prescribing patterns in a psychiatrically referred sample of youth with Autism spectrum disorder. Journal of Clinical Psychiatry. 2017;78(9):e1276-e83. |
| Siegel CE, Laska EM, Wanderling JA, Hernandez JC, Levenson RB. Prevalence and Diagnosis Rates of Childhood ADHD Among Racial-Ethnic Groups in a Public Mental Health System. Psychiatric Services. 2016;67(2):199-205. |
| Sitholey P, Agarwal V, Sharma S. An exploratory clinical study of adult attention deficit/hyperactivity disorder from India. Indian Journal of Medical Research. 2009;129(1):83-8. |
| Skokauskas N, Dunne M, Gallogly A, Clark C. Ethnic minority populations and child psychiatry services: An Irish study. Children and Youth Services Review. 2010;32(10):1242-5. |
| Soutullo CA, DelBello MP, Ochsner JE, McElroy SL, Taylor SA, Strakowski SM, Keck Jr PE. Severity of bipolarity in hospitalized manic adolescents with history of stimulant or antidepressant treatment. Journal of Affective Disorders. 2002;70(3):323-7. |
| Soutullo CA, Escamilla-Canales I, Wozniak J, Gamazo-Garran P, Figueroa-Quintana A, Biederman J. Pediatric bipolar disorder in a Spanish sample: Features before and at the time of diagnosis. Journal of Affective Disorders. 2009;118(1-3):39-47. |
| Specker SM, Carlson GA, Christenson GA, Marcotte M. Impulse control disorders and attention deficit disorder in pathological gamblers. Annals of Clinical Psychiatry. 1995;7(4):175-9. |
| Srivastava V, Girdhar R, Verma P, Arya S, Sethi S. Pattern of child and adolescent mental disorders at a tertiary care centre in North India. Journal of Indian Association for Child and Adolescent Mental Health. 2021;17(4):152-65. |
| Staller JA. Diagnostic profiles in outpatient child psychiatry. American Journal of Orthopsychiatry. 2006;76(1):98-102. |
| Sterling S, Weisner C. Chemical dependency and psychiatric services for adolescents in private managed care: implications for outcomes. Alcoholism: Clinical & Experimental Research. 2005;29(5):801-9. |
| Sternat T, Fotinos K, Fine A, Epstein I, Katzman MA. Low hedonic tone and attention-deficit hyperactivity disorder: risk factors for treatment resistance in depressed adults. Neuropsychiatric Disease & Treatment. 2018;14:2379-87. |
| Stickley A, Tachimori H, Inoue Y, Shinkai T, Yoshimura R, Nakamura J, et al. Attention-deficit/hyperactivity disorder symptoms and suicidal behavior in adult psychiatric outpatients. Psychiatry & Clinical Neurosciences. 2018;72(9):713-22. |
| Storch EA, Merlo LJ, Larson MJ, Geffken GR, Lehmkuhl HD, Jacob ML, et al. Impact of comorbidity on cognitive-behavioral therapy response in pediatric obsessive-compulsive disorder. Journal of the American Academy of Child & Adolescent Psychiatry. 2008;47(5):583-92. |
| Stralin P, Hetta J. First episode psychosis and comorbid ADHD, autism and intellectual disability. European Psychiatry: the Journal of the Association of European Psychiatrists. 2019;55:18-22. |
| Svedlund NE, Norring C, Ginsberg Y, von Hausswolff-Juhlin Y. Symptoms of Attention Deficit Hyperactivity Disorder (ADHD) among adult eating disorder patients. BMC Psychiatry. 2017;17(1):19. |
| Syed EU, Hussein SA, Yousafzai AW. Developing Services with Limited Resources: Establishing a CAMHS in Pakistan. Child & Adolescent Mental Health. 2007;12(3):121-4. |
| Syed H, Masaud TM, Nkire N, Iro C, Garland MR. Estimating the prevalence of adult ADHD in the psychiatric clinic: a cross-sectional study using the adult ADHD self-report scale (ASRS). Irish Journal of Psychological Medicine. 2010;27(4):195-7. |
| Taich A, Crowe S, Kosmorsky GS, Traboulsi EI. Prevalence of psychosocial disturbances in children with nonorganic visual loss. Journal of AAPOS. 2004;8(5):457-61. |
| Tamam L, Tuglu C, Karatas G, Ozcan S. Adult attention-deficit hyperactivity disorder in patients with bipolar I disorder in remission: preliminary study. Psychiatry & Clinical Neurosciences. 2006;60(4):480-5. |
| Tamburin S, Federico A, Morbioli L, Faccini M, Casari R, Zamboni L, et al. Screening for adult attention deficit/hyperactivity disorder in high-dose benzodiazepine dependent patients. American Journal on Addictions. 2017;26(6):610-4. |
| Testa G, Baenas I, Vintro-Alcaraz C, Granero R, Aguera Z, Sanchez I, et al. Does ADHD symptomatology influence treatment outcome and dropout risk in eating disorders? A longitudinal study. Journal of Clinical Medicine. 2020;9(7):1-11. |
| Thomson K, Randall E, Ibeziako P, Bujoreanu IS. Somatoform Disorders and Trauma in Medically-Admitted Children, Adolescents, and Young Adults: Prevalence Rates and Psychosocial Characteristics. Psychosomatics. 2014;55(6):630-9. |
| Tiwari R, Agarwal V, Arya A, Gupta PK, Mahour P. An exploratory clinical study of disruptive mood dysregulation disorder in children and adolescents from India. Asian Journal of Psychiatry. 2016;21:37-40. |
| Torres I, Gomez N, Colom F, Jimenez E, Bosch R, Bonnin C, et al. Bipolar disorder with comorbid attention-deficit and hyperactivity disorder. Main clinical features and clues for an accurate diagnosis. Acta Psychiatrica Scandinavica. 2015;132(5):389-99. |
| Tramontina S, Schmitz M, Polanczyk G, Rohde LA. Juvenile bipolar disorder in Brazil: clinical and treatment findings. Biological Psychiatry. 2003;53(11):1043-9. |
| Trivedi C, Nandakumar AL, Yousefzadehfard Y, Goriparthi TS, Chaudhari G, Vora D, et al. Suicide risk among adolescents with ADHD: An overview from the National Inpatient Sample data set. Journal of Nervous and Mental Disease. 2023;211(3). |
| Ubhi M, Achinivu K, Seri S, Cavanna AE. Motor stereotypies in adult patients with Tourette syndrome. Future Neurology. 2020;15(2) (no pagination). |
| Unver H, Karakaya I. The Assessment of the Relationship Between ADHD and Posttraumatic Stress Disorder in Child and Adolescent Patients. Journal of Attention Disorders. 2019;23(8):900-3. |
| Valsecchi P, Nibbio G, Rosa J, Tamussi E, Turrina C, Sacchetti E, Vita A. Adult ADHD: Prevalence and Clinical Correlates in a Sample of Italian Psychiatric Outpatients. Journal of Attention Disorders. 2018 Dec 20;25(4):530-9. |
| Van Ameringen M, Mancini C, Simpson W, Patterson B. Adult attention deficit hyperactivity disorder in an anxiety disorders population. CNS Neuroscience & Therapeutics. 2011;17(4):221-6. |
| van de Glind G, Koeter MW, Allsop S, Auriacombe M, Barta C, et al. Psychiatric comorbidity in treatment-seeking substance use disorder patients with and without attention deficit hyperactivity disorder: Results of the IASP study. Addiction. 2014;109(2):262-72. |
| Van Hout M, Foley M. Attention-deficit hyperactivity disorder (ADHD) in adults attending addiction treatment in Ireland: Preliminary international ADHD in substance-use disorders prevalence study (IASP) results. Irish Journal of Psychological Medicine. 2013;30(3):171-7. |
| Venkatesh C, Ravikumar T, Andal A, Virudhagirinathan BS. Attention-deficit/Hyperactivity Disorder in Children: Clinical Profile and Co-morbidity. Indian Journal of Psychological Medicine. 2012;34(1):34-8. |
| Vergara-Moragues E, Gonzalez-Saiz F, Lozano OM, Garcia AV. Psychiatric profile of three-month retention in cocaine-dependent patients treated in a therapeutic community. Journal of Studies on Alcohol and Drugs. 2013;74(3):452-9. |
| Vergara-Moragues E, Gonzalez-Saiz F, Lozano Rojas O, Bilbao Acedos I, Fernandez Calderon F, Betanzos Espinosa P, et al. Diagnosing adult attention deficit/hyperactivity disorder in patients with cocaine dependence: discriminant validity of Barkley executive dysfunction symptoms. European Addiction Research. 2011;17(6):279-84. |
| Vuijk PJ, Lee BA, Ditmars HL, Samkavitz AR, Lind HS, Doyle AE, et al. Characteristics of Child Psychiatric Outpatients at Highest Risk for Suicidal Thoughts and Behaviors. Child Psychiatry & Human Development. 2019;50(3):505-19. |
| Waluk O, Youssef G, Dowling N. The relationship between problem gambling and attention deficit hyperactivity disorder. Journal of Gambling Studies. 2016;32(2):591-604. |
| Wamulugwa J, Kakooza A, Kitaka SB, Nalugya J, Kaddumukasa M, Moore S, et al. Prevalence and associated factors of attention deficit hyperactivity disorder (ADHD) among Ugandan children; a cross-sectional study. Child & Adolescent Psychiatry & Mental Health [Electronic Resource]. 2017;11:18. |
| Watanabe-Galloway S, Rieke K, Corley B, Valleley R. Behavioral Health Problems Presented to Integrated Pediatric Behavioral Health Clinics: Differences in Urban and Rural Patients. Community Mental Health Journal. 2017;53(1):27-33. |
| Weber EB, Studeny J, Kavanaugh BC, Cook NE, Gaudet CE, McCurdy K, Holler KA. Pediatric depression symptoms, executive functioning weaknesses, and associated neuropsychological and psychiatric outcomes. Journal of Child and Family Studies. 2018;27(5):1661-70. |
| Weintraub MJ, Youngstrom EA, Marvin SE, Podell JL, Walshaw PD, Kim EY, et al. Diagnostic profiles and clinical characteristics of youth referred to a pediatric mood disorders clinic. Journal of Psychiatric Practice. 2014;20(2):154-62. |
| Welch E, Ghaderi A, Swenne I. A comparison of clinical characteristics between adolescent males and females with eating disorders. BMC Psychiatry. 2015;15:45. |
| Wentz E, Lacey JH, Waller G, Råstam M, Turk J, Gillberg C. Childhood onset neuropsychiatric disorders in adult eating disorder patients: a pilot study. European Child & Adolescent Psychiatry. 2005;14(8):431-7. |
| Wesemann D. Decreasing Rates of Pediatric Bipolar Within an Outpatient Practice. Journal of Child & Adolescent Psychiatric Nursing. 2016;29(4):188-95. |
| West SA, Strakowski SM, Sax KW, McElroy SL, Keck PE, McConville BJ. Phenomenology and comorbidity of adolescents hospitalized for the treatment of acute mania. Biological Psychiatry. 1996;39(6):458-60. |
| West SL, Mulsow M, Arredondo R. An examination of the psychometric properties of the attention deficit scales for adults with outpatient substance abusers. American Journal of Drug & Alcohol Abuse. 2007;33(5):755-64. |
| Wise BK, Cuffe SP, Fischer T. Dual diagnosis and successful participation of adolescents in substance abuse treatment. Journal of Substance Abuse Treatment. 2001;21(3):161-5. |
| Wodrich DL, Benjamin E, Lachar D. Tourette's syndrome and psychopathology in a child psychiatry setting. Journal of the American Academy of Child & Adolescent Psychiatry. 1997;36(11):1618-24. |
| Wu L-T, Gersing K, Burchett B, Woody GE, Blazer DG. Substance use disorders and comorbid axis I and II psychiatric disorders among young psychiatric patients: Findings from a large electronic health records database. Journal of Psychiatric Research. 2011;45(11):1453-62. |
| Yager J. Can trans-syndromal prototypes ("types") improve depiction of complex psychiatric cases?: An alternative way to consider concordant comorbid psychiatric disorders and their contexts as coherent units for research, assessment, and treatment planning. Journal of Nervous and Mental Disease. 2021;209(1):1-8. |
| Yamada R, Miyashita K, Hashimoto TM, Hironaka N, Takada K, Shigeta M, et al. Prevalence and Clinical Significance of Psychiatric Comorbidities With Gambling Disorder in 12 Clinical Settings in Japan. Journal of Addiction Medicine. 2023;17(2). |
| Yar A, Gundogdu OY, Tural U, Cakin Memik N. The prevalence of internet addiction in turkish adolescents with psychiatric disorders. Noropsikiyatri Arsivi. 2019;56(3):200-4. |
| Yates WR, Lund BC, Johnson C, Mitchell J, McKee P. Attention-deficit hyperactivity symptoms and disorder in eating disorder inpatients. International Journal of Eating Disorders. 2009;42(4):375-8. |
| Yellowlees PM, Hilty DM, Marks SL, Neufeld J, Bourgeois JA, Yellowlees PM, et al. A retrospective analysis of a child and adolescent eMental Health program. Journal of the American Academy of Child & Adolescent Psychiatry. 2008;47(1):103-7. |
| Yen J-Y, Liu T-L, Wang P-W, Chen C-S, Yen C-F, Ko C-H. Association between Internet gaming disorder and adult attention deficit and hyperactivity disorder and their correlates: Impulsivity and hostility. Addictive Behaviors. 2017;64:308-13. |
| Yildirim B, Fis NP, Akgul GY, Ayaz AB. Gender dysphoria and attention problems: Possible clue for biological underpinnings. Psychiatry and Clinical Psychopharmacology. 2017;27(3):289-96. |
| Yildiz D, Ciftci A, Yalcin O. Substance use patterns, psychosocial traumas, psychiatric comorbidities, and gender differences among adolescent inpatients at an addiction treatment center. Dusunen Adam. 2020;33(2):190-202. |
| Yoshida Y, Uchiyama T. The clinical necessity for assessing Attention Deficit/Hyperactivity Disorder (AD/HD) symptoms in children with high-functioning Pervasive Developmental Disorder (PDD). European Child & Adolescent Psychiatry. 2004;13(5):307-14. |
| Yoshimura A, Matsushita S, Kimura M, Yoneda J-i, Maesato H, Yokoyama A, et al. Influence of ADHD, especially attention-deficit characteristics, on the course of alcohol-dependent individuals. BMC Psychiatry Vol 22 2022, ArtID 803. 2022;22. |
| Zahid S, Bodicherla KP, Eskander N, Patel RS. Attention-Deficit/Hyperactivity Disorder and Suicidal Risk in Major Depression: Analysis of 141,530 Adolescent Hospitalizations. Cureus. 2020;12(5):e7949. |
| Zanarini MC, Athanasiadi A, Temes CM, Magni LR, Hein KE, Fitzmaurice GM, et al. Symptomatic Disorders in Adults and Adolescents With Borderline Personality Disorder. Journal of Personality Disorders. 2021;35(Supplement B):48-55. |
| Zimmerman M, Gorlin E, Dalrymple K, Chelminiski I. A clinically useful screen for attention-deficit/hyperactivity disorder in adult psychiatric outpatients. Annals of Clinical Psychiatry. 2017;29(3):160-6. |
| Zinna S, Luxton R, Papachristou E, Dima D, Kyriakopoulos M. Comorbid chronic tic disorder and Tourette syndrome in children requiring inpatient mental health treatment. Clinical child psychology and psychiatry. 2021;26(3):894-905. |

**Supplementary Figure 6: Pooled prevalence by comorbid conditions for pediatric and adult studies.**

| 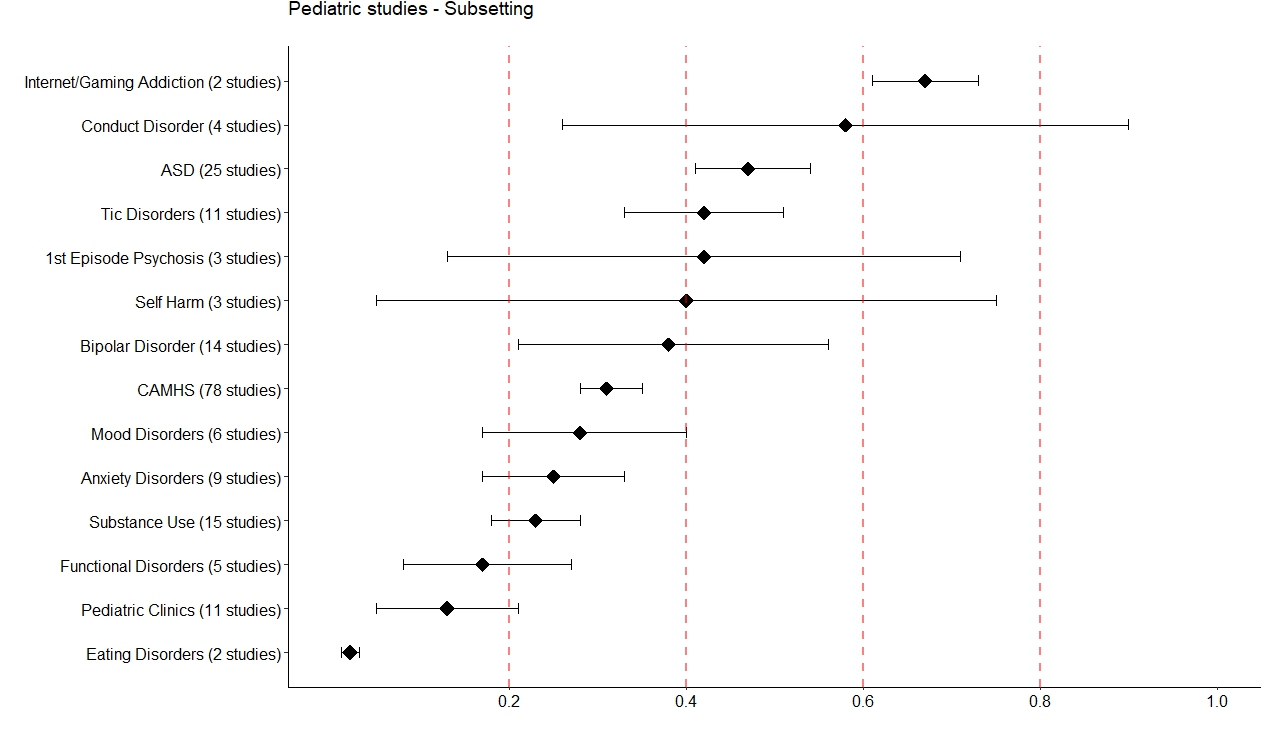 |
| --- |
| Figure 4a. Pooled prevalence of ADHD/HD by comorbid conditions in pediatric studies |
| 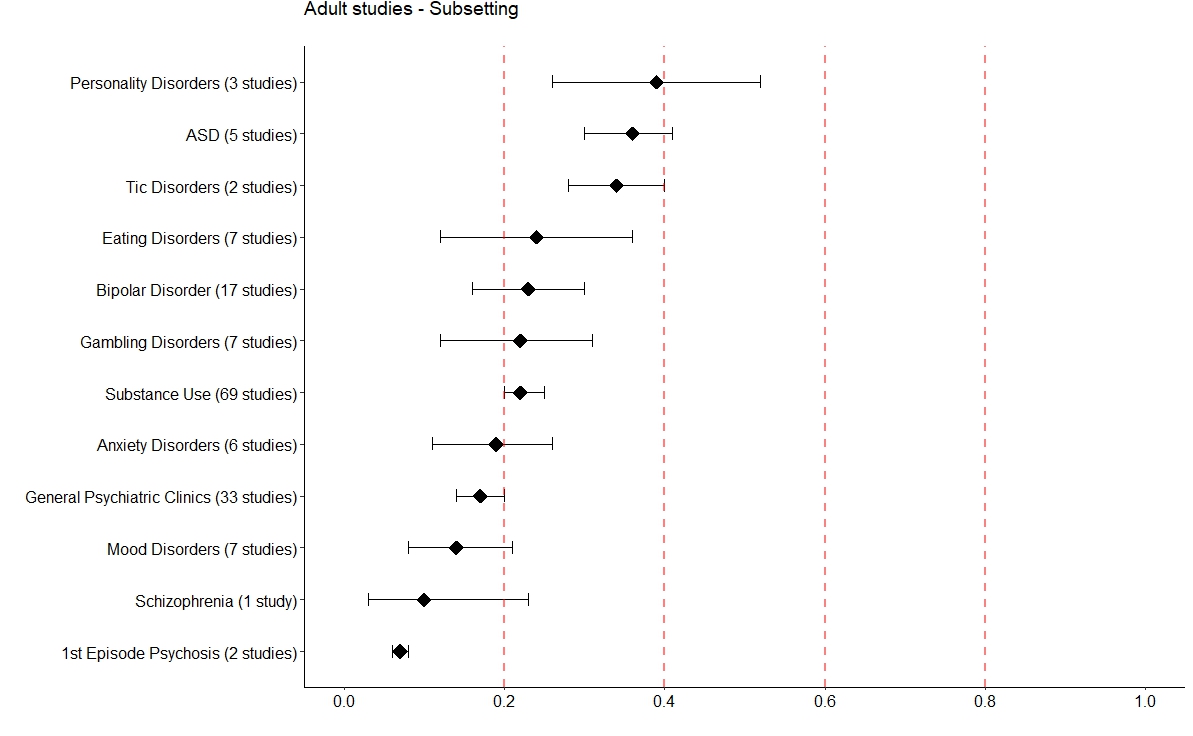 |
| Figure 4b. Pooled prevalence of ADHD/HD by comorbid conditions in adult studies |

Footnote: ASD – Autism Spectrum Disorder; CAMHS – Child and Adolescent Mental Health Services

**Supplementary Figure 7: Pooled prevalence by geographic region for pediatric and adult studies**

| 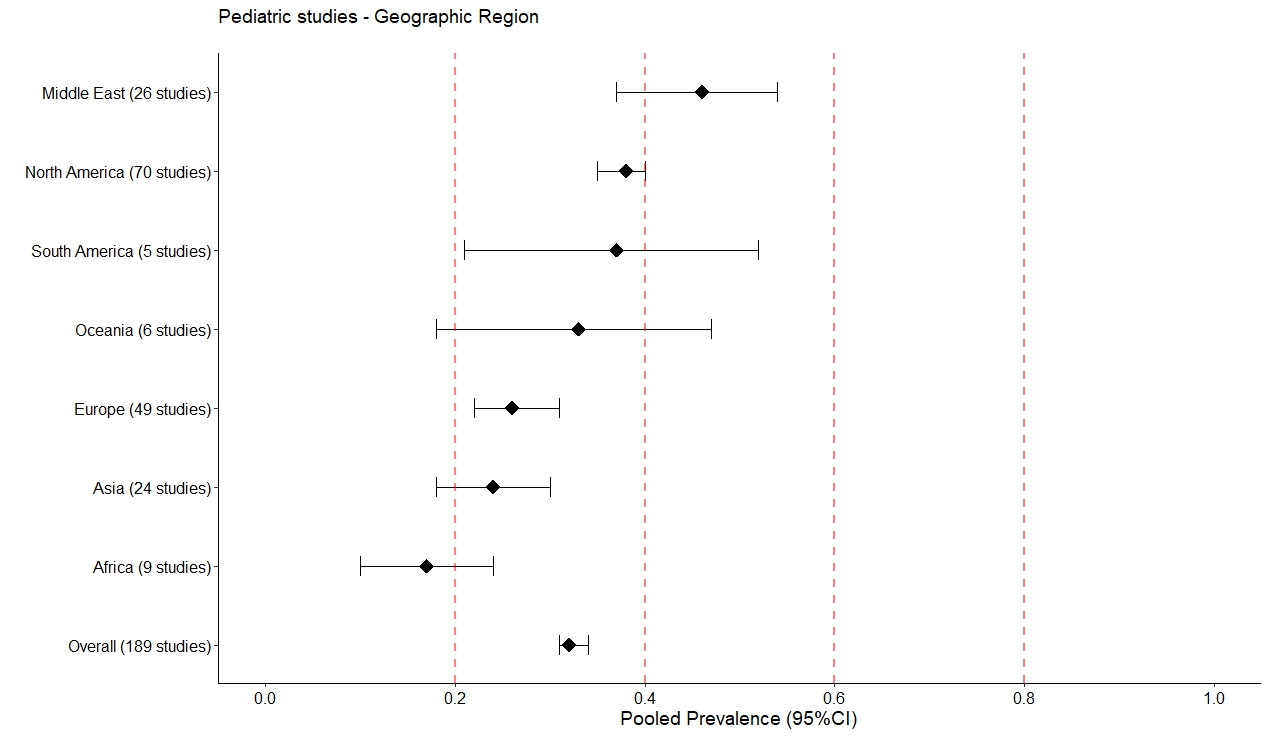 |
| --- |
| Figure 5a. Pooled prevalence of ADHD/HD by geographic regions in pediatric studies |
| 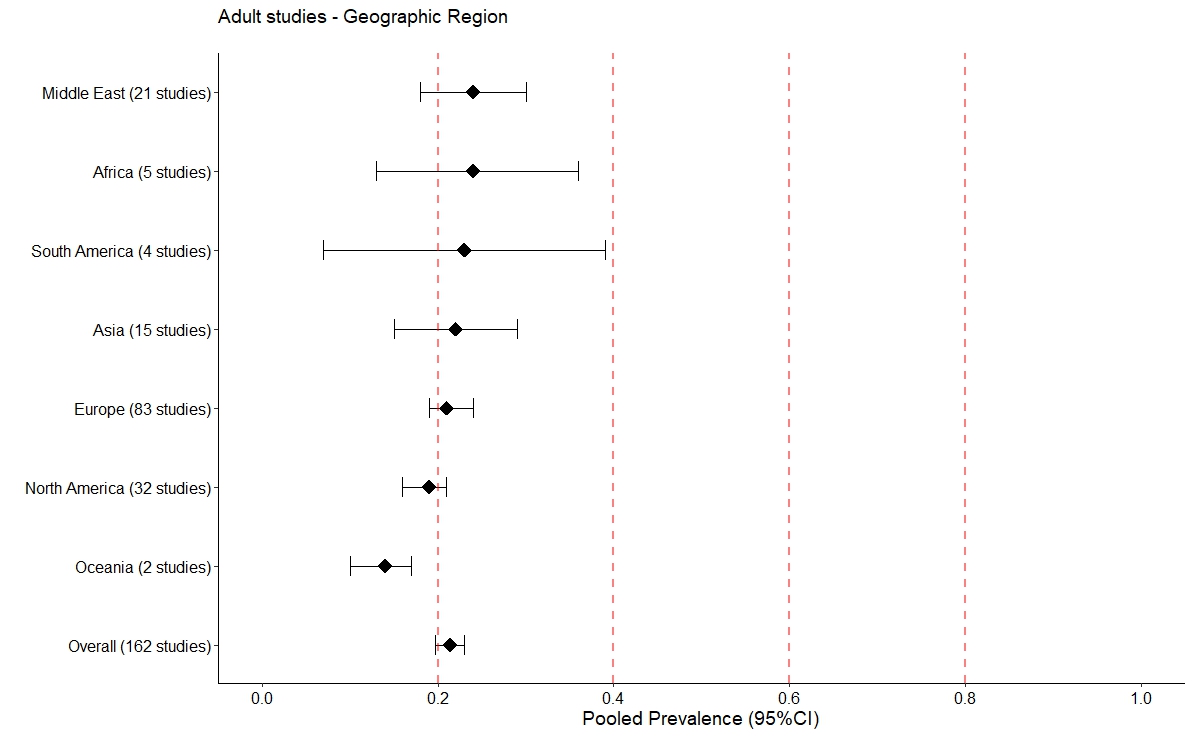 |
| Figure 5b. Pooled prevalence of ADHD/HD by geographic regions in adult studies |

**Supplementary Figure 8: Funnel Plots for pediatric and adult studies**


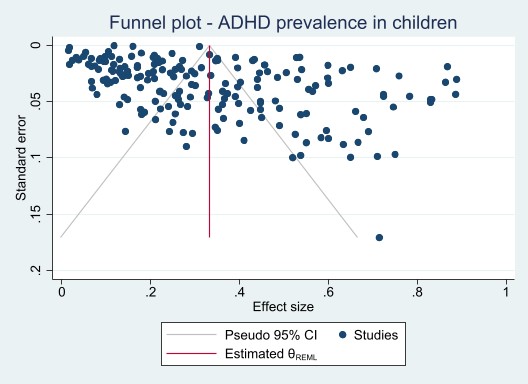


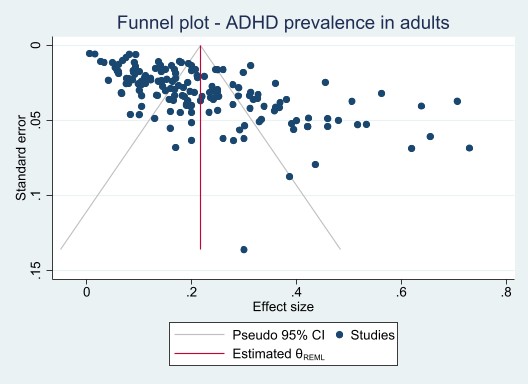

Supplement: Supplementary file 1 — Supplementary Material: Johnson et al. 2025 [file 41380_2025_3178_MOESM1_ESM.docx]
